# *Selaginella moellendorffii* has a reduced and highly conserved expansin superfamily with genes more closely related to angiosperms than to bryophytes

**DOI:** 10.1186/1471-2229-13-4

**Published:** 2013-01-03

**Authors:** Robert E Carey, Nathan K Hepler, Daniel J Cosgrove

**Affiliations:** 1 Department of Biology, Lebanon Valley College, 101 N. College Ave., Annville, PA, 17003, USA; 2 Department of Biology, The Pennsylvania State University, 208 Mueller Lab, University Park, PA, 16802, USA; 3 Program in Biochemistry and Molecular Biology, Lebanon Valley College, 101 N. College Ave., Annville, PA, 17003, USA

**Keywords:** Expansin, *Selaginella moellendorffii*, Cell wall loosening, Gene family evolution, Plant phylogenetics

## Abstract

**Background:**

Expansins are plant cell wall loosening proteins encoded by a large superfamily of genes, consisting of four families named EXPA, EXPB, EXLA, and EXLB. The evolution of the expansin superfamily is well understood in angiosperms, thanks to synteny-based evolutionary studies of the gene superfamily in *Arabidopsis*, rice, and *Populus*. Analysis of the expansin superfamily in the moss *Physcomitrella patens* revealed a superfamily without EXLA or EXLB genes that has evolved considerably and independently of angiosperm expansins. The sequencing of the *Selaginella moellendorffii* genome has allowed us to extend these analyses into an early diverging vascular plant.

**Results:**

The expansin superfamily in *Selaginella moellendorffii* has now been assembled from genomic scaffolds. A smaller (and less diverse) superfamily is revealed, consistent with studies of other gene families in *Selaginella*. *Selaginella* has an expansin superfamily, which, like *Physcomitrella*, lacks EXLA or EXLB genes, but does contain two EXPA genes that are related to a particular *Arabidopsis*-rice clade involved in root hair development.

**Conclusions:**

From sequence-based phylogenetic analysis, most *Selaginella* expansins lie outside the *Arabidopsis*-rice clades, leading us to estimate the minimum number of expansins present in the last common ancestor of *Selaginella* and angiosperms at 2 EXPA genes and 1 EXPB gene. These results confirm *Selaginella* as an important intermediary between bryophytes and angiosperms.

## Background

Expansins are plant proteins discovered via their involvement in pH-dependent wall extension [1]. In land plants these proteins are encoded by a large superfamily of genes. Expansins act non-enzymatically in the cell wall to disrupt the interactions between cellulose microfibrils and hemicelluloses [2,3]. This is thought to contribute to turgor-driven cell wall expansion during cell growth [3-5]. The original proteins characterized in this way are now known as the EXPA family of expansins. A group of grass pollen allergens was later discovered that was also capable of causing cell wall creep and became the founding members of the group now known as the EXPB family of expansins [6]. The expansin superfamily in plants has four constituent families named EXPA, EXPB, EXLA, and EXLB. While members of the EXPA and EXPB families have been shown to have characteristic expansin activity, the functions of the EXLA and EXLB (expansin-like) families, discovered via their similarity to other expansin sequences, have not yet been characterized.

Expansins are usually expressed in a tissue -specific pattern and are involved in many processes where cell wall loosening is crucial, such as growth [7-9], fruit ripening [10], pollen tube penetration of the stigma [11], root hair elongation [12], and others [13]. The proteins encoded by these genes share certain characteristic features including a signal peptide for secretion and a two-domain structure [14,15]. Expansins have been identified in all land plants that have been examined and several related but highly divergent sequences exist in unicellular green algae [16].

Previous work has demonstrated that expansin family sizes remain relatively constant among species even when the individual genes have a distinct evolutionary history [17,18]. This suggests that there is some selective advantage to having a relatively large superfamily of expansins. The evolutionary relationships between the members of this large superfamily are complicated and have proved difficult to elucidate [19], but understanding of the superfamily in angiosperms (specifically *Arabidopsis*, rice, and *Populus*) has improved through the use of genomic history to complement phylogenetic analysis [18,20]. The analysis by Sampedro *et al.*[20] indicated 17 orthologous expansin gene clades between *Arabidopsis* and rice, and revealed a dynamic gene superfamily with large numbers of gene births (due to polyploidy and segmental duplications) and deaths shaping the distribution of sequences within these clades.

An additional study elucidated the composition of the expansin superfamily in *Physcomitrella patens* and compared these sequences with angiosperm expansins [17]. Although these *Physcomitrella* expansins do not show a clear relationship to specific *Arabidopsis*-rice clades defined by the work of Sampedro *et al.*[20], they do show a gene superfamily of similar size and complexity arising from a minimum of 2 EXPA and 1 EXPB genes in the common ancestor of *Physcomitrella* and angiosperms [17]. The genome sequencing of *Selaginella moellendorffii*, an early diverging vascular plant [21] offers an opportunity to extend our understanding of this large gene superfamily into the lycophytes, a key intermediate between bryophytes and seed plants. *Selaginella,* a vascular plant with true roots and shoots has a far greater morphological similarity to angiosperm species than mosses like *Physcomitrella*. Thus, the likelihood of relating expansins of an early diverging lineage to the expansin genes of angiosperms seems greater in a study of lycophytes than bryophytes.

## Results

### Expansin superfamily in S*elaginella moellendorffii*

The expansin sequences revealed via searches of the *Selaginella* genome comprise a superfamily whose composition is similar to what has been observed in angiosperm genomes with a few notable exceptions.

Table 1 shows a comparison of the relative sizes of the families that make up the expansin superfamily (EXPA, EXPB, EXLA, and EXLB) in two fully sequenced angiosperms [20] as well as in *Populus*[18]*, Selaginella*, and *Physcomitrella*[17]*.* As is the case in *Arabidopsis*, rice and *Populus*, the EXPA family is the largest expansin family in *Selaginella*, but it is half the size found in the other species. The EXPB family of *Selaginella* is of a size (relative to the EXPA family) more consistent with that seen in *Arabidopsis*, poplar, and *Physcomitrella* and appears not to have expanded as found in the rice genome. The overall size of the *Selaginella* expansin superfamily is smaller than that of the other plants mentioned here, most likely related to its much smaller genome size of 110 Mb [21]. As was the case for *Physcomitrella*, it was not possible to identify any sequence in the trace archive for *Selaginella* that corresponds to the EXLA or EXLB family. Members of both of these families are present in pine [18], but a tBLASTx search of the available fern sequences on GenBank did not yield any results.

**Table 1:** Expansin and other selected plant gene family compositions

	***Arabidopsis thaliana***	***Oryza sativa***	***Populus trichocarpa***	***Selaginella moellendorffii***	***Physcomitrella patens***
EXPA	26	33	27	15	28
EXPB	6	18	3	2	7
EXLA	3	4	2	0	0
EXLB	1	1	4	0	0
MIP	35	33	55	19	23
XTH	33	30	41	19	31
Callose synthase	12	10	19	7	12
sequence available	complete genome	complete genome	473.1 Mbp^†^	212.5 Mbp	480 Mbp^Δ^
			22, 136 scaffolds	759 scaffolds	2,106 scaffolds
			7.5X coverage	7.0X coverage	8.1X coverage

Numbers and type of genes found in a sampling of plant species with completed or nearly completed genome sequences [17,18,25-28].

^†^ at time of search (Sampedro, Carey, & Cosgrove, 2006).

^Δ^ at time of search (Carey & Cosgrove, 2007).

**Figure 1:**
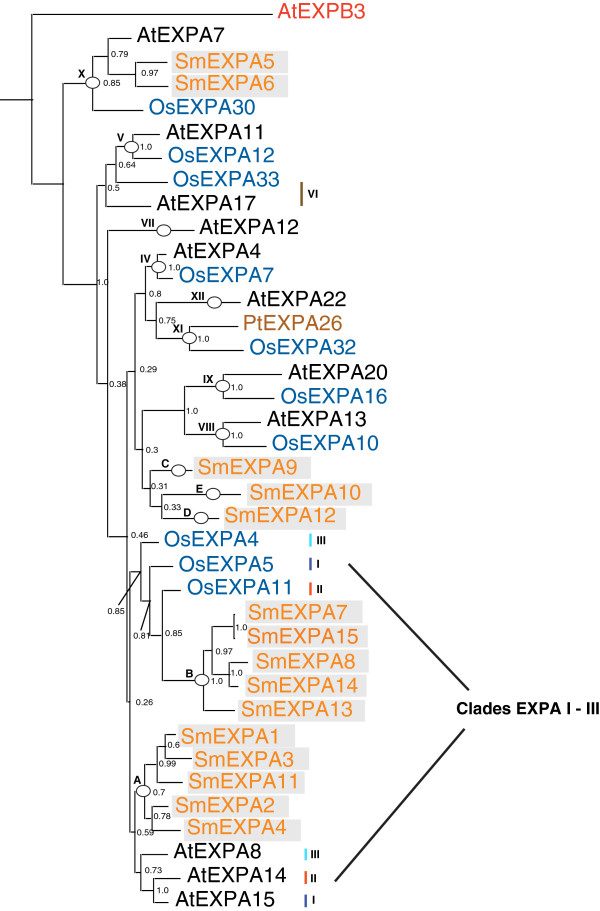
**Bayesian likelihood tree for *Selaginella *EXPA genes with selected rice, *Arabidopsis*, and *Populus *sequences.** Burnin was set to 1,000,000. Tree was manually rooted at *AtEXPB3*. Clade and groupings are marked with circles (or bars when they are poorly resolved as in the case of clades EXPA – I, EXPA – II, EXPA – III and EXPA – VI). *Selaginella* sequences are labeled in orange and boxed, rice in blue, *Arabidopsis* in black*,* and a *Populus* in brown.

It should be noted that there was a duplicate and a partial expansin sequence found in the genome search. The duplicate sequence (provisionally called *SmEXPA15* [XM_002994463.1]), the only gene on its scaffold, is identical to *SmEXPA7* [XM_002994463.1] in both haplotypes. A small portion of the coding sequence is annotated as an intron on the JGI *Selaginella moellendorffii* v1.0 genome site. We believe this to be a misannotation (see highlighted region in Additional file 1). The partial sequence, which was determined to be a pseudogene, appears to be a duplicated *SmEXPA3* [XM_002974112.1] that has since acquired numerous mutations and only contains a few regions of conserved expansin sequence. *SmEXPA15* [XM_002994463.1] branches with *SmEXPA7* [XM_002994463.1] with a posterior probability of 1 on Figure 1. *SmEXPA15* is not included in the other phylogenetic or distance analyses.

### Phylogenetic analysis of *Selaginella* expansins

**Figure 2:**
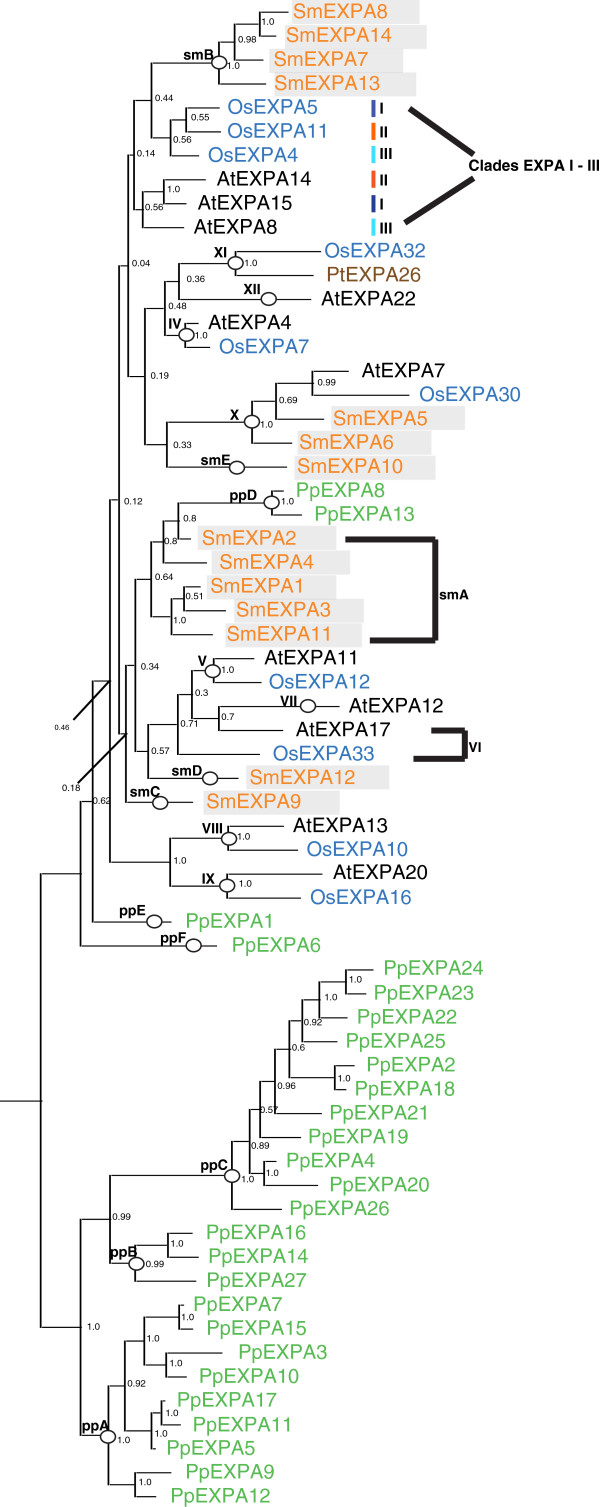
**Bayesian likelihood tree for *Selaginella *and *Physcomitrella *EXPA genes with selected rice, *Arabidopsis*, and *Populus *sequences.** Burnin was set to 500,000. Tree was rooted manually at *Physcomitrella patens* groups A – C. Clade and groupings are marked with circles (or bars when they are poorly resolved as in the case of clades EXPA – I, EXPA – II, EXPA – III, and EXPA – VI). *Selaginella* sequences are labeled in orange and boxed, rice in blue, *Arabidopsis* in black*, Physcomitrella patens* in green and a *Populus* in brown.

The 15 EXPA genes isolated from the JGI *Selaginella moellendorffii* v1.0 genome were translated into amino acid sequence and aligned with a selection of *Arabidopsis*, rice, and a single *Populus* sequence (to clarify clade EXPA – XI) representing the angiosperm clades described by Sampedro *et al.*[18,20]. This alignment (see Additional file 2) was then used to produce Bayesian, parsimony, and neighbor-joining phylogenetic trees. Only one haplotype version of each gene was used. Including both versions did not affect the topology of any tree (data not shown). A second alignment also including *Physcomitrella* sequences (see Additional file 3) was used to build Bayesian, parsimony, and neighbor-joining trees. Figure 2 shows a Bayesian likelihood phylogenetic tree based on this alignment.

Two *Selaginella* EXPA genes, *SmEXPA5* [XM_002961012.1] and *SmEXPA6* [XM_002980135.1], appear to be a sister group to the *Arabidopsis*-rice clade EXPA-X (*AtEXPA7* [NM_101127.3] and *OsEXPA30* [AC092697.6]). This grouping is corroborated by the two alternate tree-building methods (see Additional files 4 and 5). The remaining *Selaginella* EXPA sequences can be divided into five groups that have been named A-E (Figures 1, 2 and Additional files 4 and 5).

Group A contains *Selaginella* sequences *SmEXPA1* [XM_002974852.1]*, SmEXPA2* [XM_002981819.1]*, SmEXPA3* [XM_002974112.1]*, SmEXPA4* [XM_002988923.1]*, and SmEXPA11* [XM_002973901.1]. This group of *Selaginella* expansins, while not grouping consistently with a specific *Arabidopsis*-rice clade, does have the smallest pairwise distances to an angiosperm expansin of any *Selaginella* gene group (Additional file 6).

These low distances are always to the members of *Arabidopsis*-rice clades I-IV, the most conserved of all *Arabidopsis*-rice clades (indicating that they are under strong purifying selection). This group also branches (although with weak support) on all trees with *Physcomitrella* group D (Figure 2 and Additional files 7 and 8). In previous work it was observed that this *Physcomitrella* group branched with the members of *Arabidopsis*-rice clades EXPA I-III in the Bayesian trees [17]. Although it is still very poorly resolved phylogenetically, it is possible that *Selaginella* group A, *Physcomitrella* group D, and angiosperm clades EXPA I-III are orthologous groups based on the low distances and phylogenetic results described here. It is certain, however, that the genes of *Selaginella* group A are more closely related to angiosperm EXPA genes and *Physcomitrella* groups D-F than to *Physcomitrella* groups A-C.

Group B consists of five *Selaginella* EXPA genes (*SmEXPA7* [XM_002994463.1], *SmEXPA8* [XM_002968976.1], *SmEXPA13* [XM_002980028.1], *SmEXPA14* [XM_002990586.1], and *SmEXPA15* [XM_002994463.1]). While it is not possible to state with any confidence that this group of *Selaginella* expansins is a sister to a specific *Arabidopsis*-rice clade, it does seem clear that these genes group more closely with angiosperm expansins and not, for example, with the genes of *Physcomitrella* groups A-C (Figure 2 and Additional files 7 and 8).

The placement of *SmEXPA9* [XM_002963656.1], *SmEXPA10* [XM_002981332.1]*,* and *SmEXPA12* [XM_002966496.1] is poorly resolved in all phylogenetic trees. They do not clearly branch with any known rice, *Arabidopsis* or *Populus* clade. *SmEXPA10* [XM_002981332.1] does consistently branch (Figure 2 and Additional files 7 and 8) with *AtEXPA12* [XM_002882892.1], but with uniformly poor support. These *Selaginella* expansins also do not group consistently with a known pine specific group [18] or with each other. *SmEXPA9* [XM_002963656.1], *SmEXPA10* [XM_002981332.1]*,* and *SmEXPA12* [XM_002966496.1] do not consistently branch with any known *Selaginella* or *Physcomitrella* expansin either, regardless of the tree-building method employed (Figures 1, 2 and Additional files 4, 5, 7 and 8).

Thus, *Selaginella* EXPA sequences can be divided into 6 groups ranging from 1 to 5 sequences. One of these is clearly orthologous to a clade seen in *Arabidopsis*, rice, and *Populus* (EXPA-X). The five remaining groups (A-E) seem to be more closely related to angiosperm expansins than to bryophyte specific groups, but do not group consistently with any specific *Arabidopsis*-rice clade.

**Figure 3:**
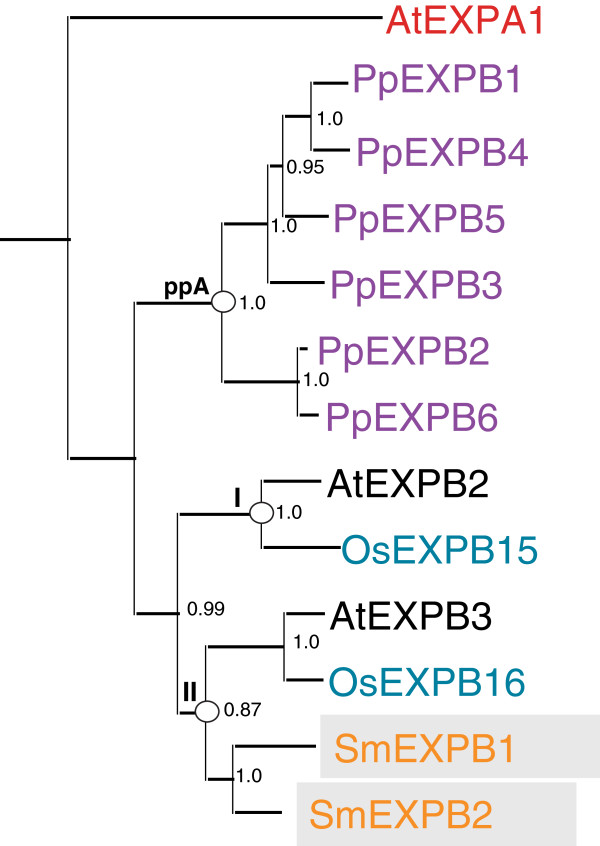
**Bayesian likelihood tree for *Selaginella *and *Physcomitrella *EXPB genes with selected rice and *Arabidopsis *sequences.** Burnin was set to 150,000. Tree was rooted manually at *AtEXPA1*. Clade and groupings are marked with circles. *Selaginella* sequences are labeled in orange and boxed, rice in blue, *Arabidopsis* in black, and *Physcomitrella patens* in purple.

The two EXPB genes isolated from the genome were translated into amino acid sequence and aligned with a selection of *Arabidopsis* and rice EXPB sequences representing the clades described by Sampedro *et al.*[20] as well as all the *Physcomitrella* EXPB sequences described in previous work [17]. This alignment (Additional file 9) was then used to produce Bayesian, parsimony, and Neighbor-joining phylogenetic trees. Figure 3 shows a Bayesian likelihood phylogenetic tree based on this alignment.

The two *Selaginella* EXPB genes (*SmEXPB1* [XM_002970263.1] and *SmEXPB2* [XM_002983273.1]) branch as a sister group to the representatives of *Arabidopsis*-rice EXPB clade II (*AtEXPB3* [NM_118965.3] and *OsEXPB16* [AK240809.1]) in the Bayesian and Neighbor-joining (but not the maximum parsimony) trees with relatively good support (Figure 3 and Additional files 10 and 11).

As was noted for the *Physcomitrella* expansin superfamily [17], the *Selaginella* expansin superfamily seems to be evolving quite independently and yet a large multigene family is maintained. This may indicate that the size of the expansin gene families is somehow critical, with the advantage of a large family becoming redundant at some maximum number.

Without including substantial gymnosperm and other intermediary expansin sequence (such as fern sequences), phylogenetic analyses comparing taxa as distantly related as *Selaginella* and angiosperms inevitably become inaccurate. Although there are some EST sequences available for *Pinus taeda*, including this limited set of gymnosperm expansins in these phylogenetic analyses does not help to resolve the placement of *Selaginella* EXPA groups (Additional files 12 and 13). Adding the very few fern expansins available in GenBank also does not improve these phylogenies (data not shown). As it becomes available, extensive gymnosperm and fern sequence will need to be included in these analyses in order to improve the reliability of the phylogenies. At present, there is substantial EST data available for loblolly pine, but no whole-genome data from any gymnosperm or fern. It should also be noted that even within the angiosperms, the difficulty in using traditional phylogenetic methods to elucidate relationships between members of the expansin families is well known [20]. This is not surprising as the expansin superfamily shows evidence of rapid diversification with many gene births and deaths [20].

Notwithstanding these difficulties, sequence-based phylogenies still offer some insight into the evolutionary relationships between the expansin sequences of *Selaginella* and angiosperms, especially when used in the light of the well-supported classifications proposed in previous work [18,20]. The classification of Sampedro *et al.*[20] will be used here to discuss the relationship of *Selaginella* expansins to their angiosperm counterparts.

### Distances of *Selaginella* expansins to angiosperm expansins

**Figure 4:**
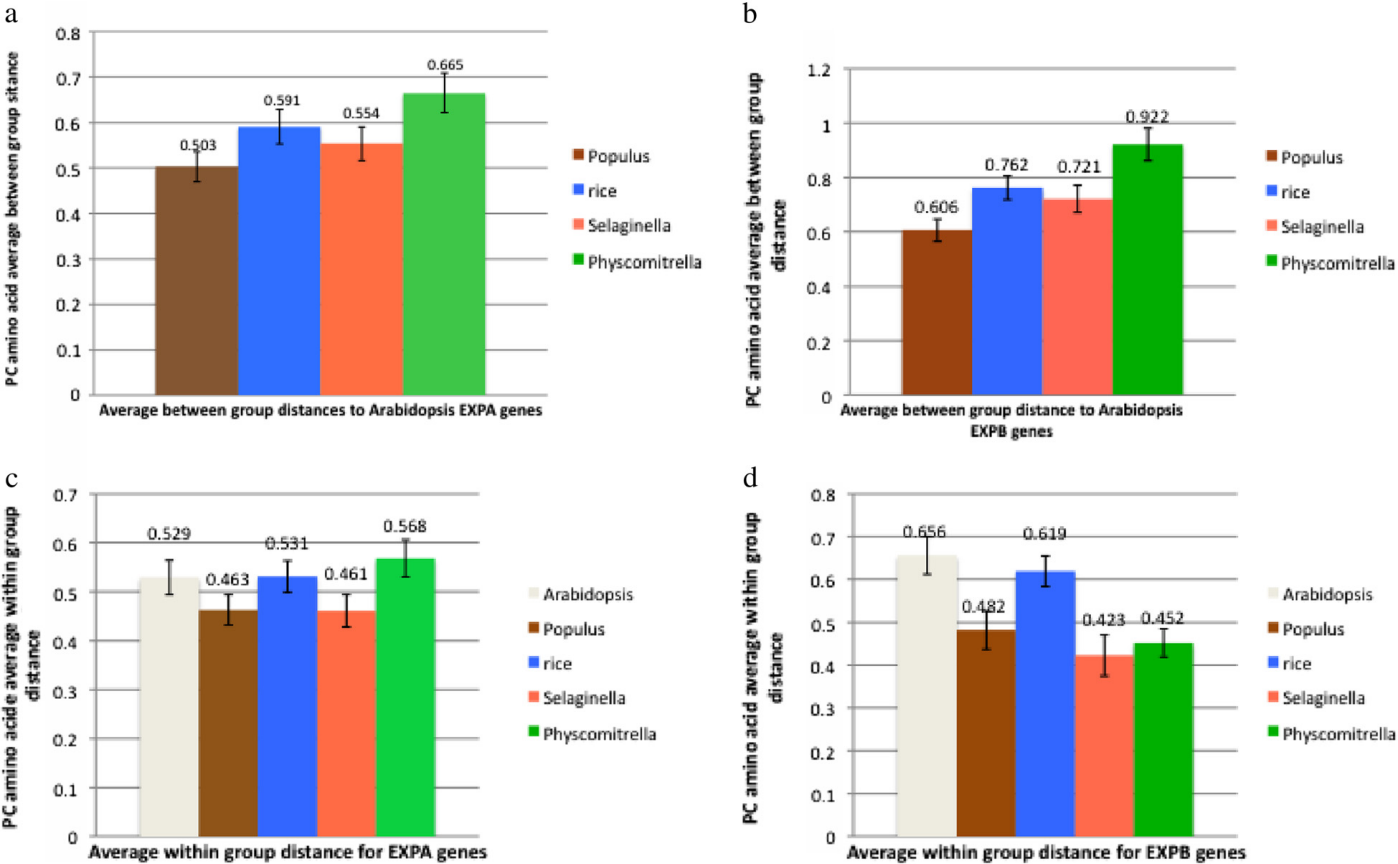
**a. Between group mean distances for the EXPA gene family.** Values indicated are the mean Poisson-corrected amino acid distance to the *Arabidopsis* EXPA family. Error bars are standard error based on 500 bootstrap replicates. **b.** Between and within group mean distances for the EXPB gene family. Values indicated are the mean Poisson-corrected amino acid distance to the *Arabidopsis* EXPB family. Error bars are standard error based on 500 bootstrap replicates. **c.** Within group mean distances for the EXPA gene family. Values indicated are the mean Poisson-corrected amino acid distance for the EXPA genes from each species. Error bars are standard error based on 500 bootstrap replicates. **d.** Within group mean distances for the EXPB gene family. Values indicated are the mean Poisson-correct amino acid distance for the EXPB genes from each species. Error bars are standard error based on 500 bootstrap replicates.

Poisson-corrected amino acid distances were calculated for each *Selaginella* expansin to each *Arabidopsis* and rice expansin (data summarized using shortest distances in Additional file 6). The average between and within group Poisson-corrected amino acid distances for both the EXPA and EXPB families were also calculated (Figures 4a,b,c,d). Figures 4a and b show that, as would be expected, the *Physcomitrella* EXPA and EXPB families have the greatest average distance to the *Arabidopsis* families. The *Selaginella* average distances are smaller than those for *Physcomitrella* and even than those of rice. Figures 4c and 4d show *Selaginella* as having the lowest within-group distances, closely followed by *Populus*, possibly indicating a greater degree of sequence conservation. This distance estimate could be influenced by codon usage bias in rice, which [18] has been confirmed as a factor in expansin amino acid usage in rice [20]. Interestingly, *Selaginella* also seems to have an elevated GC content (Additional file 14), although not to the same extent as rice. It is possible that altered amino acid composition resulting from codon bias is adding to the difficulty in resolving the relationship of *Selaginella* genes to angiosperm clades in the phylogenetic analyses presented here. It could also be that *Selaginella* expansins are under a high degree of purifying selection (slowing their rate of change), which would likely be the case if *Selaginella* only contains the most essential expansins for survival. Although the average distances of *Selaginella* expansins to their angiosperm counterparts seem fairly small, it is still not possible to place the majority of these genes as a sister group to any known angiosperm clade.

### Intron-based analysis of *Selaginella* expansins

In addition to having conserved amino acid sequence, expansins have been shown to have a fairly conserved intron pattern (see Additional file 15). Sampedro *et al.*[20] hypothesized the ancestral intron patterns for the angiosperm expansin families. Based on what was known of the intron patterns seen in *Arabidopsis* and rice, the intron pattern for ancestral EXPA and EXPB sequences was estimated using parsimony as a basis for determining the pattern (the number of gains/losses was maximized). In this way, it was hypothesized that the ancestral EXPA intron pattern likely consisted of introns ‘A’ and ‘B.’ The likely ancestral EXPB intron pattern was hypothesized to consist of introns ‘A,’ ‘B,’ ‘C’ and ‘F.’ The intron patterns of *Selaginella* lend support to the hypothesized ancestral EXPA and EXPB patterns [20] and indicates these patterns pre-date the divergence of lycophytes and angiosperms.

The fifteen *Selaginella* EXPA genes all contain introns ‘A’ and ‘B,’ which obviously supports the idea of an ‘AB’ ancestral intron pattern. Four EXPAs also contain additional novel introns: both *SmEXPA2* [XM_002981819.1, XM_002994136.1] haplotypes contain an intron in the 5^′^-untranslated region, *SmEXPA11b* [XM_002973901.1] contains an intron in the 5^′^-untranslated region and *SmEXPA1b* [XM_002988863.1] contains an intron in the 3^′^-untranslated region.

All three EXPB genes discovered in *Selaginella* have introns ‘A,’ ‘C,’ ‘B’ and ‘F.’ This data suggests that in the last common ancestor between *Arabidopsis*, rice and *Selaginella*, the intron pattern for EXPB genes may well have been ACBF, which is congruent with the findings of Sampedro *et al.*[20]. This is in contrast to the more variable intron patterns seen in *Physcomitrella*[17], and is further evidence of *Selaginella*’s value as an intermediary taxon between bryophytes and angiosperms.

*Selaginella* expansins appear to have reduced intron sizes when compared to their *Physcomitrella* and *Arabidopsis* counterparts. For example, the average size of intron A in *Selaginella* EXPA genes is 85 bp while it is longer in both *Physcomitrella* (387 bp) and *Arabidopsis* (158 bp). Intron B shows a similar pattern, with *Selaginella* having the shortest introns (although *Arabidopsis* has longer average length than *Physcomitrella* for intron B). This reduction of intron size is consistent with previous observations that reduced genome size correlates with a decreased size of non-coding regions [22].

## Discussion

The sequencing of the *Selaginella moellendorffii* genome allows us to fill in some of the gaps in our knowledge of early land plant expansin evolution. Using phylogenetic analyses, it has been possible to predict some of the types of expansins found in the last common ancestor of *Selaginella* and *Arabidopsis*. The pattern of introns seen in *Selaginella* is also useful for determining the pattern of intron evolution in the EXPA and EXPB families, with *Selaginella* having a pattern consistent with previous predictions about expansin intron evolution.

### Superfamily composition

As seen in Table 1 there are some differences between the compositions of the expansin superfamily in *Selaginella* compared to what is seen in angiosperms or *Physcomitrella*. One of the most obvious differences is the apparent lack of members of the EXLA and EXLB family in *Selaginella* and *Physcomitrella*. This likely indicates that these families arose after the divergence of *Physcomitrella* and *Selaginella*, as the presence of these genes was not detected by a tBLASTX search of the JGI *Selaginella moellendorffii* v1.0 genome. It is doubtful that these families are ancestral to all land plants, as they would have to have been lost in multiple independent lineages. It is more likely that EXLA and EXLB gene families arose after the divergence of lycophytes and bryophytes. The sequencing of basal vascular plants, ferns, and gymnosperms will help clarify this issue. It will be interesting to see at what point these gene families first appeared as more sequence becomes available.

We also see in Table 1 that the proportion of genes belonging to the EXPA and EXPB families in *Selaginella* is similar to what is found in *Arabidopsis*, *Populus*, and *Physcomitrella* but not in rice [18,20]. The diversity of cell wall composition among land plants is likely important in shaping the diversity of the expansin superfamily, and it may be that the expansion of the EXPB family in rice is related to the unique cell walls of grasses [23,24]. The overall size of the *Selaginella* superfamily is smaller than is seen in the other plants studied here. Reduced gene family size is not limited to expansins in *Selaginella* but has also been observed in the non cell wall-related gene family of major intrinsic proteins [25] and the cell wall-related gene families of callose synthase [26,27] and xyloglucan endo-transglycosylase/hydrolase (XTH) [28]. Current research also shows no evidence that *Selaginella* has undergone whole genome duplication or a polyploidy event [21], which would limit the number of expansin genes as compared to other plant species as polyploidy is known to be an important driving force in expansin evolution [20]. This may mean that the smaller expansin superfamily of *Selaginella* has changed much more slowly and may represent a more “essential set” of expansins.

### Phylogenetic analysis of the *Selaginella* expansin superfamily

From the phylogenetic trees for the *Selaginella* EXPA genes with selected EXPA sequences from rice, *Arabidopsis*, and *Populus* (Figure 1), we see that one group of *Selaginella* EXPA genes branches clearly as a sister group to the *Arabidopsis*-rice clade EXPA – X. *SmEXPA5* [XM_002961012.1] and *SmEXPA6* [XM_002980135.1] clearly branch sister to *Arabidopsis*-rice clade EXPA – X, a clade whose *Arabidopsis* genes have a well-characterized expression pattern [12] that is root hair specific. *Selaginella* does have root hairs, [29] and it would be a confirmation of the functional orthology of these genes if they were expressed there. It is possible that this particular type of expansin may have evolved from the need to regulate root hair development once these organs arose in land plant lineages (*Selaginella* has true roots while *Physcomitrella* does not).

The members of group A have the smallest pairwise distances of any group to the three most conserved *Arabidopsis*-rice clades (EXPA I – III). These *Arabidopsis*-rice clades along with *Arabidopsis*-rice clade EXPA IV are what were initially characterized as Subgroup A [30] and may function in vasculature tissue, specifically xylem [31]. The members of *Arabidopsis*-rice clades I, II, III, and IV are consistently the genes with the smallest pairwise distances to *Selaginella* EXPA sequences (the exceptions being *SmEXPA5* [XM_002961012.1] and *SmEXPA6* [XM_002980135.1]). They also have the smallest within and between group mean distances when compared with the other *Arabidopsis*-rice clades. These data suggest that the members of these *Arabidopsis*-rice clades are under strong purifying selection. Despite this overall similarity to many of the basal land plant EXPA genes seen in *Selaginella*, the members of group A have noticeably smaller distances to the members of *Arabidopsis*-rice clades EXPA I – III (Additional file 6). If members of this *Selaginella* EXPA group were shown to be expressed in vascular tissue, it might indicate that these genes are orthologous to the members of *Arabidopsis*-rice clades I – IV and raise the possibility that these genes have developed a function important in xylem development in tracheophytes. Group A seems to also group consistently, with relatively good support, with *Physcomitrella* group D. This may support an association of group A and angiosperm clades EXPA 1 – III as *Physcomitrella* group D shows weak branching with *Arabidopsis*-rice clades EXPA I – III on some trees [17].

The remaining *Selaginella* EXPA groups are not clearly sister groups to any particular angiosperm clade or *Physcomitrella* grouping, but do seem to be more closely related to angiosperm expansins and *Physcomitrella* groups D – F than to *Physcomitrella* groups A – C (Figures 1, 2 and Additional files 4, 5, 7 and 8). When all *Selaginella* EXPA genes are constrained as a monophyletic group and a parsimony analysis is performed, maximum parsimony trees of length 1939 (38 steps longer than the tree in Additional file 4) are obtained. When all of the *Selaginella* EXPA genes except for *SmEXPA5* [XM_002961012.1] and *SmEXPA6* [XM_002980135.1] are constrained as a monophyletic group, maximum parsimony trees of length 1910 are obtained. This would seem to indicate that there are relationships amongst these *Selaginella* groups and angiosperm clades that phylogenetic analyses do not yet clearly resolve, and it also is consistent with the idea that *SmEXPA5* [XM_002961012.1] and *SmEXPA6* [XM_002980135.1] are sister to clade EXPA-X.

Thus, although it is likely an underestimation, we conclude that the last common ancestor of *Selaginella* and angiosperms had two EXPA genes, one that gave rise to *SmEXPA5* [XM_002961012.1] and *SmEXPA6* [XM_002980135.1] and one that gave rise to the rest of the *Selaginella* EXPA gene family.

The two *Selaginella* EXPB genes group with *Arabidopsis*-rice clade EXPB – II in Bayesian and Neighbor Joining trees, indicating at least one EXPB in the common ancestor of *Selaginella* and angiosperms that is more similar to the vegetative EXPBs of angiosperms than to *Physcomitrella* EXPBs.

### *Selaginella* expansin distance analysis

Additional file 6 shows that nearly all *Selaginella* EXPA genes have their lowest pairwise distance to a member of *Arabidopsis*-rice clades I – IV, again potentially suggesting that they are under greater purifying selection. The genes of group A have particularly small distances to the members of these clades, perhaps suggesting some relationship that is not yet apparent in phylogenetic analyses. It is also interesting to note that nearly the only exceptions to this pattern are *SmEXPA5* [XM_002961012.1] and *SmEXPA6* [XM_002980135.1] whose smallest pairwise distances are to members of clade EXPA – X, the one with which they branch as a sister group to in phylogenetic analyses.

*Selaginella* EXPA and EXPB genes have surprisingly small average distances to their angiosperm counterparts (Figure 4a,b). These rather small evolutionary distances do not alleviate the difficulty of phylogenetic analysis mentioned previously, however.

### Intron analysis of the *Selaginella* expansin superfamily

All *Selaginella* EXPA genes show an ‘AB’ intron pattern, with four haplotypes showing additional introns. Both haplotypes for *SmEXPA2* [XM_002981819.1, XM_002994136.1] and one haplotype for *SmEXPA11* (designated *SmEXPA11b* [XM_002973901.1]) contain an intron in the 5^′^ – untranslated region. The *SmEXPA2* [XM_002981819.1] introns and *SmEXPA11b* [XM_002973901.1] intron are relatively the same length, located in the same area of the 5^′^ - UTR and are nearly a 45% match on the nucleotide level, so we’ve decided that they are probably the same. They have been designated novel intron prime, n’. *Arabidopsis*-rice EXPA clades I – II do contain an intron in the 5^′^ – UTR [20], so it is possible that n’ is that same intron. However, since none of the other *Selaginella* EXPA genes contain n’, that is not likely the case. More likely n’ is a novel intron that arose in a subset of *Selaginella* group A and has been lost in one *SmEXPA11* [XM_002973901.1] haplotype. Also, one haplotype of *SmEXPA1* (designated *SmEXPA1a* [XM_002974852.1]) contains an intron in the 3^′^ – untranslated region. This intron has been designated novel intron, n.

Both *Selaginella* EXPB genes show an ‘ACBF’ intron pattern, which is the ancestral intron pattern predicted in Sampedro *et al.*[20] for these families based on a parsimony model of intron gain and loss in angiosperms. The ‘AB’ intron pattern seen in all the *Selaginella* EXPA genes is also the predicted ancestral intron pattern [20]. These data therefore support this predicted ancestral intron pattern at least as far back as the last common ancestor of *Selaginella* and *Arabidopsis*.

### Conservation of amino acid sequence

As was seen for the EXPA gene family in *Physcomitrella*[17], *Selaginella* also shows conservation at all normally conserved expansin amino acid residues. In contrast to the EXPB family in *Physcomitrella*[17], the *Selaginella* EXPB gene family also shows conservation at these sites. This would seem to imply that the biochemical function of *Selaginella* EXPA and EXPB genes is not altered from the biochemical function of these gene families in angiosperms. It is worth noting that recent work has demonstrated the importance of xyloglucan in both acid growth and expansin activity assays [32] and that lycophytes have a very different xyloglucan composition than eudicots, gymnosperms, and some ferns [33]. It is possible that these differences in xyloglucan composition are not important for expansin function in lycophytes, or that there is some subtle systematic difference in lycophyte expansins that is not immediately obvious.

## Conclusions

With the extensive analysis of rice, *Arabidopsis*, and *Populus* as a guide, the classification of *Selaginella* expansins into groups and the inference of the relationship of these groups to known orthologous groups in *Arabidopsis* and rice, and homologous groups of genes observed in *Physcomitrella* has been attempted. What is seen is an expansin superfamily in *Selaginella* that is somewhat more easily related than *Physcomitrella* expansins to the groups of expansin genes seen in higher plants. Indeed, *Selaginella* expansins seem to have much more in common with their *Arabidopsis* and rice counterparts than they do with *Physcomitrella*. Evidence indicates that some *Selaginella* genes are sister groups to *Arabidopsis*-rice clades. In addition, all *Selaginella* expansins seem to be more closely related to angiosperm expansins and *Physcomitrella* groups D – F than to the bryophyte – specific groups described previously [17]. Thus a picture emerges of morphological similarity potentially reflecting expansin superfamily development, with morphologically similar plants having more similarities in their expansin families. This makes sense given the closer evolutionary relationship of morphologically similar plants and the importance of expansins in growth and developmental processes. The smaller and less diverse *Selaginella* expansin superfamily may prove useful as a vehicle for understanding the “essential set” of expansins needed for plant growth and development. As more and more plant species are sequenced in the genomics age, what are now mere outposts of data will be interconnected, hopefully with the result of elucidating the dynamic evolutionary past of gene superfamilies such as expansins.

## Methods

### Trace archive searches

Trace archives for *Selaginella moellendorffii* (1,814,554 traces on 10/08/2005) were searched using the “Cross-species Mega BLAST” on the NCBI Trace Archive Nucleotide BLAST website [34]. All *Arabidopsis*, rice, and known *Physcomitrella* sequences were used as BLAST queries under default parameters. The traces identified by these searched were downloaded in .scr trace format for assembly into contigs. All *Selaginella* expansins isolated in this way were then used to search the archive. An additional tBLASTX search of the archives was done using EXLA and EXLB genes from *Arabidopsis*, rice, and pine as search queries (thanks to K. Wall).

### Assembly of contigs

Trace files were assembled into contigs with the SeqMan application in the DNASTAR software package. The ends of the traces were trimmed on the ‘high’ quality setting (quality score = 16). The alignments were created with a minimum match percentage of 90% over 50 base pairs. Assembly was performed after the completion of all searches.

### Genome search

The *Selaginella* genes originally assembled from the trace archive were used to search the *Selaginella moellendorffii* v1.0 genome [35]. A tBLASTX search was also conducted using all *Arabidopsis*, rice, and *Physcomitrella* expansin sequences. The traces identified by these searches were downloaded in .fasta format and cross checked to eliminate duplicate results. The *Selaginella* genome (both haplotypes) was analyzed using the resulting sequences to identify expansin genes. Sequences that did not encode genes were discarded. Sequences that correctly encoded expansin genes were downloaded in .fasta format, compared to previously isolated *Selaginella* expansins and named accordingly (see Additional file 16). All expansin annotations were inspected for intron patterns. Sequences were then trimmed for alignment.

### Phylogenetic tree construction

*Selaginella* sequences (Additional file 1) were aligned with selected *Arabidopsis*, rice, and sometimes *Physcomitrella* sequences [17]. Alignments were generated via the Clustal W function of the MegAlign application of the DNASTAR 9 software package with default alignment parameters (Gonnet Series protein weight matrix, gap penalty of 15, gap length penalty of 6.66, delay Divergent Seqs 30%). These alignments (Additional files 2, 3 and 15) were then used as the input to generate Bayesian, parsimony, and neighbor-joining phylogenies trees.

MrBayes version 3.1.2p [36,37] was utilized using the POOCH software application [38] to generate the Bayesian trees (Jones amino acid model, gamma rates, 2 runs, 4 Markov chains – number of generations and burnin as indicated in figure legends) from an alignment trimmed from a conserved tryptophan following the signal peptide to a conserved phenylalanine at the carboxyl terminus of the expansin genes. MCMC convergence was assessed graphically using the AWTY web service [39]. The consensus trees were then visualized using the Tree Graph 2 software application [40] and manually rooted.

Protein parsimony trees were made using the same alignment with the Phylogenetic Analysis Using Parsimony software package (PAUP* version 4.0) [41]. Maximum parsimony trees were generated by a heuristic search with 100 random sequence additions. A bootstrap analysis with 500 replicates was then performed with 10 search replicates with random additions per bootstrap replicate. The Tree Graph 2 software application [40] was then used to visualize the consensus trees and manually root them. If the bootstrap consensus contained adequate information it is used in the figure. If many branches in the consensus tree were poorly resolved then one of the maximum parsimony trees was used with bootstrap values manually added to nodes with good support in the bootstrap consensus tree.

Neighbor-Joining trees were constructed using the MEGA Phylogeny software version 5.05 [42]. The alignments were trimmed as described previously. Poisson-corrected amino acid distance with complete deletion of gaps was the distance method employed in the trees constructed. Confidence values given are bootstrap values based on 1000 bootstrap replicates. The trees were manually rooted.

### Calculation of between and within group average distances

Amino acid alignments of all *Populus*, *Arabidopsis*, rice, *Selaginella*, and *Physcomitrella* EXPA and EXPB sequences were used to determine the between group and within group mean Poisson-corrected amino acid distances using MEGA 5.05. Standard error was also calculated for these values using 500 bootstrap replicates.

## Competing interests

The authors declare no competing interests of any kind.

## Author’s contributions

REC participated in the design of the study, performed the original trace archive searches, generated alignments, built phylogenetic trees, and drafted the manuscript. NKH performed genome searches, generated alignments and phylogenies, generated the between and within group distance analysis, and helped draft the manuscript. DJC conceived the study, and participated in its design and coordination and helped to draft the manuscript. All authors read and approved the final manuscript.

## Supplementary Material

Additional file 1 ***Selaginella*expansin sequences acquired from JGI *Selaginella moellendorffii v*1.0 genome.** Named genes and their intron patterns are followed by their nucleotide and amino acid sequence. Introns in nucleotide sequence are black, coding region is in red, and untranslated regions in blue. Click here for file

Additional file 2 **Alignment for Figure **1**.**
Alignment of *Selaginella* EXPA sequences with selected *Arabidopsis*, rice, and a *Populus* EXPA gene. Click here for file

Additional file 3 **Alignment for Figure **2**.** Alignment of *Selaginella* and *Physcomitrella* EXPA sequences with selected *Arabidopsis*, rice and a *Populus* EXPA gene. Click here for file

Additional file 4 **One of four maximum parsimony *Selaginella* EXPA trees of length 1901. **Significant bootstrap values from bootstrap consensus tree are indicated. Tree was rooted manually at *AtEXPB3*. Clade and groupings are marked with circles (or bars when they are poorly resolved as in the case of clades EXPA – I, EXPA – II, EXPA – III, and EXPA – VI). *Selaginella* sequences are labeled in orange and boxed, rice sequences in blue, *Arabidopsis* in black, and a *Populus* sequence in brown. Click here for file

Additional file 5 **Neighbor joining tree for the *Selaginella* EXPA family.** Tree was rooted manually at *AtEXPB3*. Clade and groupings are marked with circles (or bars when they are poorly resolved as in the case of clades EXPA – I, EXPA – II, EXPA – III, and EXPA – VI). *Selaginella* sequences are labeled in orange and boxed, rice sequences in blue, *Arabidopsis* in black, and a *Populus* sequence in brown. Click here for file

Additional file 6 **The lowest Poisson-corrected amino acid distance of each *Selaginella* EXPA gene to an *Arabidopsis* and rice expansin.** The clades from which each of these *Arabidopsis* and rice genes come from is also given. Clades highlighted in yellow are those other than EXPA I-IV. Note that *SmEXPA5* and *SmEXPA6* have their lowest distance to members of clade EXPA – X. Click here for file

Additional file 7 **One of thirty-four maximum parsimony *Selaginella* and *Physcomitrella *EXPA trees of length 2871.** Significant bootstrap values from bootstrap consensus tree are indicated. Tree was rooted manually at *Physcomitrella patens* groups A-C. Clades and groupings are marked with circles (or bars when they are poorly resolved as in the case of clades EXPA – I, EXPA – II, EXPA – III and EXPA – VI or the tree has become cluttered as in the case of clades smD and smE). *Selaginella* sequences are labeled in orange and boxed, *Physcomitrella patens* in green, rice sequences in blue, *Arabidopsis* in black, and a *Populus* sequence in brown. Click here for file

Additional file 8 **Neighbor joining tree for *Selaginella* and *Physcomitrella* EXPA family.** Tree was rooted manually at *Physcomitrella patens* groups A-C. Clade and groupings are marked with circles (or bars when they are poorly resolved as in the case of clades EXPA – I, EXPA – II, EXPA – III, and EXPA – VI). *Selaginella* sequences are labeled in orange and boxed, *Physcomitrella patens* in green, rice sequences in blue, *Arabidopsis* in black, and a *Populus* sequence in brown. Click here for file

Additional file 9 **Alignment for Figure **3**.** Alignment of *Selaginella* and *Physcomitrella* EXPB sequences with selected *Arabidopsis* and rice EXPB genes. Click here for file

Additional file 10 **Neighbor joining tree for EXPB family.** Tree was rooted manually at *AtEXPA1*. Clade and groupings are marked with circles. *Selaginella* sequences are labeled in orange and boxed, *Physcomitrella patens* in purple, rice in blue, and *Arabidopsis* in black. Click here for file

Additional file 11 **Bootstrap consensus parsimony tree obtained for the EXPB family.** Tree was rooted manually at *AtEXPA1*. The original analysis recovered one most parsimonious tree of length 796. Clade and groupings are marked with circles. *Selaginella* sequences are labeled in orange and boxed, *Physcomitrella patens* in purple, rice in blue, and *Arabidopsis* in black. Click here for file

Additional file 12 **Bayesian likelihood tree for *Selaginella* and *Physcomitrella* EXPB genes with selected rice, *Arabidopsis, Populus*, and gymnosperm sequences.** 40,000 trees were collected. Burnin was set to 10,000. Clade and groupings are marked with circles. *Selaginella* sequences are orange and boxed, *Physcomitrella* in purple, rice in blue, and *Arabidopsis* in black. Gymnosperm sequences are green. ‘TC’ numbers are TIGR numbers for Pinus ESTs. Wmi is *Welwitschia mirabilis* from the Floral Genome Project (http://www.floralgenome.org/). Click here for file

Additional file 13 **Bayesian likelihood tree for *Selaginella* and *Physcomitrella*EXPA genes with selected rice, *Arabidopsis***, ***Populus*, and gymnosperm sequences.** Clade and groupings are marked with circles. *Selaginella* sequences are orange and boxed, *Physcomitrella* in green, rice in blue, and *Arabidopsis* in black and *Populus* in brown. Gymnosperm sequences are in red. ‘TC’ numbers are TIGR numbers for Pinus ESTs. Wmi and zfi are *Welwitschia mirabilis* and *Zamia fisheri* cDNA from the Floral Genome Project (http://www.floralgenome.org/). Click here for file

Additional file 14 **Average nucleotide composition of *Arabidopsis*, rice, *Selaginella*, and *Physcomitrella* EXPA genes. **Values given are a percentage of all nucleotides in a dataset trimmed as was done for the phylogenies presented here. Click here for file

Additional file 15 **Location of expansin introns. Diagram showing the location of expansin introns.** The relative locations of G and n’ is ambiguous. Figure adapted from Sampedro and Cosgrove [11]. Click here for file

Additional file 16 **GenBank Accession Numbers.** Accession numbers for Arabidopsis, rice, *Selaginella*, *Physcomitrella*, and *Populus* sequences used for alignments and phylogeny building. Note that not all *Physcomitrella* sequences have GenBank entries. Please consult the *Physcomitrella* genome v1.1 (http://genome.jgi-psf.org/physcomitrella/physcomitrella.info.html) for missing sequences. Click here for file
