# Supplementary material for: Selaginella moellendorffii has a reduced and highly conserved expansin superfamily with genes more closely related to angiosperms than to bryophytes
Source: BMC Plant Biol. 2013 Jan 3;13:4. doi: 10.1186/1471-2229-13-4 (PMC3680112; doi:10.1186/1471-2229-13-4)
Supplement: Additional file 1 — Selaginellaexpansin sequences acquired from JGI Selaginella moellendorffii v1.0 genome. Named genes and their intron patterns are followed by their nucleotide and amino acid sequence. Introns in nucleotide sequence are black, coding region is in red, and untranslated regions in blue. [file 1471-2229-13-4-S1.docx]

Nucleotide Sequence:

AAAA – Coding region

AAAA – Intron

AAAA – Untranslated regions

SmEXPA1a [ABn]

ATGAATTCATGGTATTCCAGGTCGCCAGTTCTCGCTGCTCTTGGATTTCTTCTCCACTGTACGCTGTTCCAGTTCGCTCAGGGACAAGGGTGGACAAGCGCTCATGCAACTTTCTATGGGGGAAGCGATGCAGCAGGGACAATGGGTACGTAATGCGTGAAGTTTTCTACAGTGAAGAGTTATAATATATGTGTGATGTTCTTCTTTTGTTTGTAGGTGGAGCTTGTGGTTATGGTAATTTGTACAGCCAAGGATACGGCAACAACAATGCCGCACTGAGCACTGCTTTGTTCAACTCTGGATTGAGCTGTGGTGCGTGCTTTGAGATCCGGTGTGACAGTGCTGCCGATCCAAGATGGTGCATTGCCGGGACTTCCGTTGTTGTCACTGCAACCAACTTCTGCCCTCCAAACTACGCTCTCGCAAACAACAACGGAGGATGGTGCAACCCTCCACTGGAACACTTCGACATGGCGCAGCCTGCGTGGGAGCAAATTGGCATCTACAGGGGAGGCATTGTTCCCGTCCAATACAGAAGGTAAAGTCGAAAGCCTTTAAAAGCCGCTCATAACTTTTTCTCGTTTGGTGGATAACAGAGTCAGCTGCGTCAAGAAGGGAGGAATCCACTTCACAATGAACGGCCACACGTATTTCAATCTGGTGCTCATCAGCAACGTTGGCGGAGCAGGCGACGTGCACGCCGTGTCCATCAAAGGCTCCGGCACCGGTTGGCAAGACATGAGCCGGAACTGGGGACAAAACTGGCAGAGCAATGGTCAGTTCCAGGGGCAGAGCCTCTCGTTCAGAGTCACCACGAGCGATGGCAAAAGCGTGGTCTCCATGGACGTGGCTCCCGCCGACTGGCAGTATGGACAAACCTTCGAAGGGTCCCAGTTTGATTGAAAAGTACGTTGTTTTTCTCTGGAGCTTTTCTGGTTCTCGTCCTGTGCTTATGGCTGGCTGTGTTGCAGGGATCGAAGGAAAGAGATGGAAGAGATGGAAGACATGCTGGAAGATGGAAGAGGAGGTAAAAGTTTCATTACATCCATGGAGGAAACTAAGAGCGTAGAGGGTCTAGAAAGGATGGGTTTTTAGCGCAGATTTGAGGATGGGGATGAGATGAAGAAACGACGGCCAACCTCCCCGCCGCTGGAAGAGATTTTAGAGATTCTAGAGAAGCTCGAGGAACAGTTTGCAAAGTCCAAATCGAGCAGACGAGCTCCATCACGCTGTCGGGAGTTTGCAAAGAGTCACGGGGAAAAATAGAAGATAAAAGGAAACGAGGCGAGCGTTATGAAGCTCTTGTGCACTGCATCGAAACCAAAAGAGAAAAGCTTTCTT

MNSWYSRSPVLAALGFLLHCTLFQFAQGQGWTSAHATFYGGSDAAGTMGGACGYGNLYSQGYGNNNAALSTALFNSGLSCGACFEIRCDSAADPRWCIAGTSVVVTATNFCPPNYALANNNGGWCNPPLEHFDMAQPAWEQIGIYRGGIVPVQYRRVSCVKKGGIHFTMNGHTYFNLVLISNVGGAGDVHAVSIKGSGTGWQDMSRNWGQNWQSNGQFQGQSLSFRVTTSDGKSVVSMDVAPADWQYGQTFEGSQFD

SmEXPA1b [AB]

ATGAATTCATGGTATTCCAGGTCGCCAGTTCTCGCTGCTCTTGGTTTTCTTCTCCACTGTACGCTGTTCCAGTTCGCTCAGGGACAAGGGTGGACAAGCGCTCATGCAACTTTCTATGGGGGAAGCGATGCAGCAGGGACAATGGGTACGTAATGCGTGAAGTTTTCTACAGTGAAGAGTTATAATATATGTGTGATGCTCTTCTTTTGTTTGTAGGTGGAGCTTGTGGTTATGGTAATTTGTACAGCCAGGGATACGGCAACAACAATGCCGCACTGAGCACTGCTTTGTTCAACTCTGGATTGAGCTGTGGTGCGTGCTTTGAGATCCGGTGTGACAGTGCTGCCGATCCAAGATGGTGCATTGCCGGGACTTCCGTTGTTGTCACTGCAACCAACTTCTGCCCTCCAAACTACGCTCTCGCAAACAACAACGGAGGATGGTGCAACCCTCCACTGGAACACTTCGACATGGCGCAGCCTGCGTGGGAGCAAATTGGCATCTACAGGGGAGGCATTGTTCCCGTCCAATACAGAAGGTAAAGTCGAAAGCCTTTAAAAGCCGCTCATAACTTTTTCTCGTTTGGTGGATAACAGAGTCAGCTGCGTCAAGAAGGGAGGAATCCACTTCACAATGAACGGCCACACGTATTTCAATCTGGTGCTCATCAGCAACGTTGGCGGAGCCGGCGACGTGCACGCCGTGTCCATCAAAGGCTCCGGCACCGGTTGGCAAGACATGAGCCGGAACTGGGGACAAAACTGGCAGAGCAATGGCCAGTTCCAGGGGCAGAGCCTCTCGTTCAAAGTCACCACGAGCGATGGCAAAAGCGTGGTCTCCATGGACGTGGCTCCCGCCGACTGGCAGTATGGACAAACCTTCGAAGGGTCCCAGTTTGATTGA

MNSWYSRSPVLAALGFLLHCTLFQFAQGQGWTSAHATFYGGSDAAGTMGGACGYGNLYSQGYGNNNAALSTALFNSGLSCGACFEIRCDSAADPRWCIAGTSVVVTATNFCPPNYALANNNGGWCNPPLEHFDMAQPAWEQIGIYRGGIVPVQYRRVSCVKKGGIHFTMNGHTYFNLVLISNVGGAGDVHAVSIKGSGTGWQDMSRNWGQNWQSNGQFQGQSLSFKVTTSDGKSVVSMDVAPADWQYGQTFEGSQFD

SmEXPA2a [n’AB]

GAGCTCACGATCAATCAAGCGTTCTTGGCGAGCGCGTCTCTCTCGCCTCCTTCGATCGATTTGGTATCTTTCTCTCCATTCTCGCATATAATCCTCTCTCGCCGCGGCTAATCTGTGTGAAATCTCTCGAAACATCGCAGGCCATGGCTTCCAGGGCGCCTCTGCTGCTCGCGATCGTTTCTTGGATCGCATTTCTCCACCAGGCCGCGGCGACTGGCCACCACGGCGTGGCGCGCGGCCATCACCAGCATCGCGGAGGCGGCCACGGCGGCGGCGGTGGCGGATCGTCCGGAGGAGGCTATGAATCCGGCTCCTGGACCGGCGCGCATGCTACATTCTACGGCGGCAGCGATGCATCTGGAACCATGGGTGAGGCATCGCGTTTCTTGCGAAATATTTCGCTCGATCTTGGTTTCTTGTTCTAACTGGAGCCCTCGCCTTGGATCCTCTCTACCAGGAGGAGCTTGCGGCTATGGCAATCTCTACAGCCAGGGCTACGGCACCAACACCGCGGCATTGAGCACCGCGCTCTTCCAGAGCGGCCTCTCCTGCGGCGCTTGCTTCGAGGTGAAATGCAATGGTGATCCCGAGTGGTGCCTCCCGGGCTCCTCTGTTCTCGTCACGGCCACGAATTTCTGCCCGCCCAACGACGCGCTCCCGAACAACAATGGCGGATGGTGCAACACGCCGCTCCAGCATTTCGACATGGCGCAGCCAGCGTTCGAGCAGATCGCCAAGTACAGGGGAGGCATTGTGCCTGTTCTTTATAGAAGGTACAAGCACTACATTCCCATTTTCCATTTTTTCTCTTTGCTCTTAGATCAAACGCTTGGTTGTTTTGTGTTGCTTTGCTTCTTCTATGTGCCAGTCCCATCTAGATCAAACTTTTGGTTGCTTTGCTTTGCGAAACTTTTGTTTGCTTGGCTTTGCTTGGCTTCTTCCCTGTTCATCGAGATGAGATCCTTTAGAGAAAGTCATAGCACCCACATTGCTCTGCTTTGCTTTGCTTTTATTTACGTCAGGCTTTTCTTCGTTTCAGGGTTCCATGCCAGAGGAAAGGCGGCATCCGATTCACCATGAACGGCCACAACTACTTCAACCTGGTGCTCGTCACCAACGTTGGCGGCGCCGGGGACGTCCACTCGGTTTCGATCAAGGGCTCCAACACCGACTGGCTGCCAATGTCGCGCAACTGGGGCCAAAACTGGCAGAGCAACGCCATCCTCAGCGGCCAGTCGCTCTCCTTCAAGGTCACCACCAGCGACGGCCGGACGGTGGTGTCGTACGACGCTGCCCCGCCCAATTGGCAGTACGGGCAGACTTACAGCGGCGACCAGTTCTGAGCTCTCTGATCCATCGACTCGACGCCGCTGGCTTTGGATTCCCTGGGTGGTGTTTTTTTTTTTTTCCCCCCTCCTTTCCTCGTTCTTCGCTCTTTTCTCTTTCGGTGGATTTTGGATTTTGGTGGTGGCAGCAGCGCAGCAGCAGATTTGAGAGCTGGGATGTGGGATCTCACAAACAGCCCGTGTCTGCGGCAGCAATTACGCCACCCCGTCCGATCTTGGATGCTGCGGTTTAGTTTTTTAAGGCAAAGTAGCCACCCGCATTCGAGGCATTCCTTGGTTCTCTTCCACAAGATTTTGTTTTCTCTTTTTTACACCCTTTTCTTCGACCAGCTCCTCTGGTTACTTGATTTTGTTCTTGATTCTTGGTGGCTGCCGAGGTGGTGGTGGAACACGACTACCCGCACCCACGAGAACAAAATATACTGGTTTTCTAGTACGTATCGCAGCCAAGGGAACGTGATCTCCTTCCTGTCACAAAGGATCACAGGACTTTTGGCGTTTGTTTTTGTTTCACAAAGCTTGATATGAATATAACTTACTAGGTTATAGTTATGGG

MASRAPLLLAIVSWIAFLHQAAATGHHGVARGHHQHRGGGHGGGGGGSSGGGYESGSWTGAHATFYGGSDASGTMGGACGYGNLYSQGYGTNTAALSTALFQSGLSCGACFEVKCNGDPEWCLPGSSVLVTATNFCPPNDALPNNNGGWCNTPLQHFDMAQPAFEQIAKYRGGIVPVLYRRVPCQRKGGIRFTMNGHNYFNLVLVTNVGGAGDVHSVSIKGSNTDWLPMSRNWGQNWQSNAILSGQSLSFKVTTSDGRTVVSYDAAPPNWQYGQTYSGDQF

SmEXPA2b [n’AB]

CTCACGATCAATCAAGCGTTCTTGGCGAGCGCGTCCCTCTCGCCTCCTTCGATCGATTTGGTATCTTTCTCTCCATTCTCGCATATAATCCTCTCTCGCCGCGGCTAATCTGTGTGAAATCTCTCGAAACATCGCAGGCCATGGCTTCCAGGGCGCCTCTGCTGCTCGCGATCGTTTCTTGGATCGCATTTCTCCACCAGGCCGCGGCGACTGGCCACCACGGCGTGGCGCGCGGCCATCACCAGCATCGCGGAGGCGGCCATGGCGGCGGCGGTGGCGGATCGTCCGGAGGAGGCTACGAATCCGGCTCCTGGACCGGCGCGCATGCTACATTCTACGGCGGCAGCGATGCATCTGGAACCATGGGTGAGACATCGCGTTTCTTGCGAAATATTTCGCTCGATCTTGGTTTCTTGTTCTAACGGGAGCCCTCGTCTTGGATTCTCTCTACCAGGAGGAGCTTGCGGCTATGGCAATCTCTACAGCCAGGGCTACGGCACCAACACCGCGGCATTGAGCACCGCGCTCTTCCAGAGCGGCCTCTCCTGCGGCGCTTGCTTCGAGGTGAAATGCAATGGTGATCCCGAGTGGTGCCTCCCGGGCTCCTCTGTTCTCGTCACGGCCACGAATTTCTGCCCGCCCAACGATGCGCTCCCGAACAACAATGGCGGATGGTGCAACACGCCGCTCCAGCATTTCGACATGGCGCAGCCAGCGTTCGAGCAGATCGCCAAGTACAGGGGAGGCATTGTGCCTGTTCTTTACAGAAGGTACAAGCACTACATTCCCATTTTCCATTTTTTTCTTTGCTCTTGGGTTGGGTTTTCTTTGTCCTAGTTCCATCTGGATCAGACTTTTGCTTGCTTTGCGTTGCGTTGCTTTGCTTCTATGTGCCAGTCCCATCTAGATCAAACTTTTGGTTGCTTTGCTTCTATGTAACTAGATGAAACTTTTGTTTGCTTTGCTTTGCTTCCCTGTTCATCGAGATGAGATCCTTTAGAGAAAGTCATAGCACCCACATTGCTCTGCTTTGCTTTGCTTTTATTTACGTCAGGCTTTTCTTCGTTTCAGGGTTCCATGCCAGAGGAAAGGCGGCATCCGATTCACCATGAATGGCCACAACTACTTCAACCTGGTGCTCGTCACCAACGTCGGCGGCGCCGGGGACGTCCACTCGGTTTCGATCAAGGGCTCCAACACCGACTGGCTGCCAATGTCGCGCAACTGGGGCCAAAACTGGCAGAGCAACGCCATCCTCAGCGGCCAGTCGCTCTCCTTCAAGGTCACCACCAGCGACGGCCGGACGGTGGTGTCGTACGACGCTGCCCCGCCCAATTGGCAGTACGGGCAGACTTACAGCGGCGACCAGTTCTGA

MASRAPLLLAIVSWIAFLHQAAATGHHGVARGHHQHRGGGHGGGGGGSSGGGYESGSWTGAHATFYGGSDASGTMGGACGYGNLYSQGYGTNTAALSTALFQSGLSCGACFEVKCNGDPEWCLPGSSVLVTATNFCPPNDALPNNNGGWCNTPLQHFDMAQPAFEQIAKYRGGIVPVLYRRVPCQRKGGIRFTMNGHNYFNLVLVTNVGGAGDVHSVSIKGSNTDWLPMSRNWGQNWQSNAILSGQSLSFKVTTSDGRTVVSYDAAPPNWQYGQTYSGDQF

SmEXPA3a [AB]

ATGGTGATCGCAGTTCACACTGGAAAAGCAATCGCTCAGCAATGGACGGATGCACATGCAACTTTCTACGGTGGCAGCGATGCTTCAGGCACAATGGGTATGCACCTTGGGTTTTCCAAGTTTGTAAAACCTCTCATCCCTTTTCTAGCTTTATCTTTTAAAAACAATCTTCGTTACGACTTTGCAGGAGGTGCTTGCGGGTATGGCAACCTTTACAGCCAAGGATACGGCACCAACACGGCGGCATTGAGTACCGTGCTGTTCAATTCGGGCCTGAGCTGCGGTGCGTGCTTCGAGATTAAGTGCAACGCTGCAAAAGACCCGCAGTGGTGCCGAGCTGGAGCCTCCGTCACGGTGACAGCCACGAACTTCTGTCCTCCAAACTACGCTCAGGCCAACGATAATGGCGGCTGGTGCAATCCTCCCTTGGAGCACTTCGACATGGCCCAGCCAGCATGGGAACAGATTGGCATCTACCGAGGAGGAATTGTTCCCGTCCAATACAGAAGGTACTCTTCGCTTCTTTCATCCTTTTCTCGCTTTTCTTCTTTCTCTTTTTTACTTGGCTGTGCTATGCACGCACGTAGATTCTATTCTTTACTGATTTCTCTTTCTTTTCTGTATTGCAGGGTGAGCTGCGTGAAGAAGGGAGGGATCCACTTCACGCTCAACGGGAACAAATACTTCATGCTGGTGCTCGTGAGCAACGTTGGAGGGGCCGGGGACGTGCGAGCGGTGTCGATCAAGGGGCCGAGCGGCGACTGGCAGCCAATGTCGAGGAACTGGGGCCAAAACTGGCAGAGCGATAGCAGGCTCATCGGGCAGAGCCTCTCGTTCCGCGTCGTGACGAGTGACAATCGAGCGGTCACGTCGCTCAACGTCGCACCGGCGGGGTGGAGCTTTGGCCAGACTTTCAGTGGAGAACAGTTTTGA

MVIAVHTGKAIAQQWTDAHATFYGGSDASGTMGGACGYGNLYSQGYGTNTAALSTVLFNSGLSCGACFEIKCNAAKDPQWCRAGASVTVTATNFCPPNYAQANDNGGWCNPPLEHFDMAQPAWEQIGIYRGGIVPVQYRRVSCVKKGGIHFTLNGNKYFMLVLVSNVGGAGDVRAVSIKGPSGDWQPMSRNWGQNWQSDSRLIGQSLSFRVVTSDNRAVTSLNVAPAGWSFGQTFSGEQF

SmEXPA3b [AB]

ATGGTGATCGCAGTTCACGCTGGAAAAGCACTCGCTCAGCAATGGACGGATGCACATGCAACTTTCTACGGTGGCAGCGATGCTTCAGGCACAATGGGTATGCACCTTGGGTTTTCCAAGTTTGTAAAACCTTTCATCCATTTTCTAGCTTATCTTTTAAAAACAATCTTCGTTACGACTTTGCAGGAGGTGCTTGCGGGTATGGCAACCTTTACAGCCAAGGATACGGCACCAACACGGCGGCATTGAGTACCGTGCTGTTCAATTCGGGCCTGAGCTGCGGTGCGTGCTTCGAGATTAAGTGCAACGCTGCAAAAGACCCGCAGTGGTGCCGAGCTGGAGCCTCCGTCACGGTGACAGCCACGAACTTCTGTCCTCCAAACTACGCTCAGGCCAACGATAATGGCGGCTGGTGCAATCCTCCCTTGGAGCACTTCGACATGGCCCAGCCAGCATGGGAACAGATTGGCATCTACCGAGGAGGAATTGTTCCCGTCCAATACAGAAGGTACTCTTCGCTTCTTTCTCTCTTTTCTTCTTTCTCTTTTTTACTTGGCTGTGCTATGCACGCACGTAGATTCTATTCTTTACTGATTTCTCTTTCTTTTCTATATTGCAGGGTGAGCTGCGTGAAGAAGGGAGGGATCCACTTCACGCTCAACGGGAACAAATACTTCATGCTGGTGCTCGTGAGCAACGTTGGAGGGGCCGGGGACGTGCGAGCCGTGTCGATCAAGGGGCCGAGCGGCGACTGGCAGCCATTGTCGAGGAACTGGGGCCAAAACTGGCAGAGCGATAGCAGGCTCATCGGGCAGAGCCTCTCGTTCCGCGTCGTGACGAGTGACAATCGAGCGGTCACGTCGCTCAACGTCGCACCGGCGGGGTGGAGCTTTGGCCAGACTTTCAGTGGAGAACAGTTTTGA

MVIAVHAGKALAQQWTDAHATFYGGSDASGTMGGACGYGNLYSQGYGTNTAALSTVLFNSGLSCGACFEIKCNAAKDPQWCRAGASVTVTATNFCPPNYAQANDNGGWCNPPLEHFDMAQPAWEQIGIYRGGIVPVQYRRVSCVKKGGIHFTLNGNKYFMLVLVSNVGGAGDVRAVSIKGPSGDWQPLSRNWGQNWQSDSRLIGQSLSFRVVTSDNRAVTSLNVAPAGWSFGQTFSGEQF

SmEXPA4a [AB]

ATGGCCAGCCTTTCTGCCTCTTTCAAGATCTTCATGGCACTCTTGCTAAGCAGAGTAGTCCATGGTGGTGGCTATGGAAGTGGTTGGACTGATGCCCATGCGACCTTTTATGGTGGATCCAATGCAGCAGGCACAATGGGTAAAAATCTTCAAATCCTTTCCCTCCAACATTTTAACATATTGGGTTTCTATTTGTTCCAGGTGGTGCCTGTGGCTATGGAAACTTGGTGAGTGCTGGATATGGCACCAACACAGCAGCATTGAGCACTGCTCTGTTTCAAGATGGGCTGAGCTGCGGAGCCTGCTTTGAAGTCAAGTGTGCAAGTGGCAGTGATCCCAAGTGGTGCCTCCCAGGCTCTGTGGTTGTCACAGCCACAAACTTCTGCCCTCCAAGTTCCCAACCCAGCAACGATGGGGGCTGGTGCAACTCCCCCCTCCAGCACTTTGACATGGCCCAGCCTGCATTTCTCAAGATTGCACAATACTCTGCTGGGATTGTCCCCATCTCCTACAGAAGGTAGAAATCCAGTCACCTCTGTTCCCATTTTGAGTATGTCGTCTGAACTTTGACACCCCATCCCCCTCACTGTCCGACATCTTGTCCCCTGTTCTTTCAGAGCGTTTCCTGGAGTTTTTCTGAGACCCACCTTCACTTTCTGTCTGAACTCGGGAGCTGCTCACGTTTGGTGTCCAGAAAGCTTTTCTACCTTACTGTTTCTCTTGCCAGTCTCTTTAGAGTTTAGATTCTTTCAAACTGACTTGACTTCATCCATCTTCCTCTCTCTTGGATCTCTTCTTTTTCCAAACTAAGAAAAGTTTCCTGGTTTTCTTGCAGAGTCTCTTGCTCTAGGAGTGGTGGCATCCGATTCACCATGAACGGCCACGCCTACTTCAACCTGGTGCTAATCACCAACGTTGGCGGTGCCGGGGACGTGCACGCCGTCTCCATCAAAGGCTCGGGCACCGACTGGATCCCCATGAGCCGCAACTGGGGCCAAAACTGGCAGAGCAACGCTCTCCTGGGCGGCCAGGCCCTCTCCTTCAAGGTCACAACCAGCGATGGCAAAACCACCATCGCCTACAACGTCGCCGGGGCAAACTGGGCGTATGGGCAGACGTTCGAGGGCGAGCAGTTCTAG

MASLSASFKIFMALLLSRVVHGGGYGSGWTDAHATFYGGSNAAGTMGGACGYGNLVSAGYGTNTAALSTALFQDGLSCGACFEVKCASGSDPKWCLPGSVVVTATNFCPPSSQPSNDGGWCNSPLQHFDMAQPAFLKIAQYSAGIVPISYRRVSCSRSGGIRFTMNGHAYFNLVLITNVGGAGDVHAVSIKGSGTDWIPMSRNWGQNWQSNALLGGQALSFKVTTSDGKTTIAYNVAGANWAYGQTFEGEQF

SmEXPA4b [AB]

ATGGCCAGCCTTTTTGCCTCTTTCAAGATCTTCATGGCACTCTTGCTAGGCAGAGTAGTCCATGGTGGTGGCTATGGAGGTGGTTGGACTGATGCCCATGCGACCTTTTATGGTGGATCCAATGCAGCAGGCACAATGGGTAAAAATCTTCAAATCCTTTCGCTCCAACATTTTAACATATTGGGTTTCTATTTGTTCCAGGTGGTGCCTGTGGCTATGGAAACTTGGTGAGTGCTGGATATGGCACCAACACAGCAGCATTGAGCACTGCTCTGTTTCAAGATGGGCTGAGCTGCGGAGCCTGCTTTGAAGTCAAGTGTGCAAGTGGCAGTGATCCCAAGTGGTGCCTCCCAGGCTCTGTGGTTGTCACAGCCACAAACTTCTGCCCTCCAAGTTCCCAACCCAGCAACGATGGGGGCTGGTGCAACTCCCCCCTCCAGCACTTTGACATGGCCCAGCCTGCATTTCTCAAGATTGCACAATACTCTGCTGGGATTGTCCCCATCTCCTACAGAAGGTAGAAATCCAGTCACCTCTGTTCCCATTCTGAGTATGTCGTCTGAACTTTGACACCCCATCCCCCTCACTGTCCAACATCTTGTCCACTGTTCTTTCAGAGCGTTTCCTGGAGCTTTTCTGAGACCCACCTTCACTTTCTGTCTGAACTCGGGAGCTGCTCACGTTTGGTGTCCAGAAAGCTTTTCTACCTTACTGTTTCTCTTGCCAGTCTCTTTAGAGTTTAGATTCTTTCAAACTGACTTGACTTCATCCATCTTTCTCTCTCTTGGATCTCTTCATTTTCCAAACTAAGAAAAGTTTCCTGGTTTTCTTGCAGAGTCTCTTGCTCTAGGAGTGGTGGCATCCGATTCACCATGAACGGCCACGCCTACTTCAACCTGGTGCTAATCACCAACGTTGGCGGTGCCGGGGACGTGCACGCCGTCTCCATCAAAGGCTCGGGCACCGACTGGATCCCCATGAGCCGCAACTGGGGCCAAAACTGGCAGAGCAACGCTCTCCTGGGCGGCCAGGCCCTCTCCTTCAAGGTCACAACCAGCGATGGCAAAACCACCATCGCCTACAACGTCGCCGGGGCAAACTGGGCGTATGGGCAGACGTTCGAGGGCGAGCAGTTCTAG

MASLSASFKIFMALLLSRVVHGGGYGSGWTDAHATFYGGSNAAGTMGGACGYGNLVSAGYGTNTAALSTALFQDGLSCGACFEVKCASGSDPKWCLPGSVVVTATNFCPPSSQPSNDGGWCNSPLQHFDMAQPAFLKIAQYSAGIVPISYRRVSCSRSGGIRFTMNGHAYFNLVLITNVGGAGDVHAVSIKGSGTDWIPMSRNWGQNWQSNALLGGQALSFKVTTSDGKTTIAYNVAGANWAYGQTFEGEQF.

SmEXPA5a [AB]

ATGAGGAGTGTGATCGCGTGTCTTCTTCTCTGCGCCACTCTTCTCGTGTCTGTGGATCATGGGAGAGCGGCATCGCCCAAAAGCTTGAAGAAGCGGGCCACGAACGCCGCGAAGGAGATCTACAACGCCGGCGGGTGGAAGCAGGCATTCGCGACGTTCTATGGCGATGAAACAGCGAGGGAGACAATGGGTAAAGAAGAAACTTTCGAGATCATATTTAAATTTGCGCAGGTGGAGCTTGCGGCTATGGCAATTTGTACCAATCCGGCTATGGATTGATGACGGCGGCGCTGAGCAGTACGCTCTTCAACAGCGGCTATGGCTGCGGGCAGTGCTACGAGATCACTTGCACGCTATCGAAGCACTGCTACTTCGGAAAATCCGTGGTGGTGACCGCCACAAATCTTTGCCCGCCCAATTGGAGCAAGCCCTCGAACAATGGCGGCTGGTGCAATCCGCCGCGCGTCCATTTCGACATGTCCAAGCCGGCATTCATGAAAATCGCGTTTTGGCGGGCGGGAATCATCCCAGTCTCCTATCGAAGGTGAGAGAAGAAAAACGCGCGAGATTTTAGGGTTCTTCAGCTAAAAGTTTGAAAACTTTCCTGTCAGGGTGCCATGCGTGCGATCCGGCGGCATGAATTTCAAGCTGGGCGGCAATCGCTGGTGGCTCATGGTGTTCATCACAAATGTGGGCGGCTCCGGTGATATCAAGGCGGTGTCGGTGAAGGGATCGAGGACGGGATGGATCGCCATGACGCGGAATTGGGGCGTAGGGTTCCAAGTTTTCAAGCAGCTCCAGGGCCAAAGTCTCTCGTTCATGGTCACTTGCTACAGTACCGGAAAGACTACTGTGCACAACAATGTGGCGCCCGCGAATTGGCAGCTCGGCAGCACCTATTCCGCCAAGCAATTGTGA

MRSVIACLLLCATLLVSVDHGRAASPKSLKKRATNAAKEIYNAGGWKQAFATFYGDETARETMGGACGYGNLYQSGYGLMTAALSSTLFNSGYGCGQCYEITCTLSKHCYFGKSVVVTATNLCPPNWSKPSNNGGWCNPPRVHFDMSKPAFMKIAFWRAGIIPVSYRRVPCVRSGGMNFKLGGNRWWLMVFITNVGGSGDIKAVSVKGSRTGWIAMTRNWGVGFQVFKQLQGQSLSFMVTCYSTGKTTVHNNVAPANWQLGSTYSAKQL

SmEXPA5b [AB]

ATGAGGAGTGTGATCGCGTGTCTTCTTCTCTGCGCCACTCTTCTCGTGTCTGTGGATCATGGGAGAGCGGCATCGCCCAAAAGCTTGAAGAAGCGGGCCACGAACGCCGCGAAGGATATCTACAACGCCGGCGGGTGGAAGCAGGCGTTCGCGACGTTCTATGGCGATGAAACAGCGAGGGAGACAATGGGTAAAGAAGAAACTTTCGAGATCATATTTAAATTTGCGCAGGTGGAGCTTGCGGCTATGGCAATTTGTACCAATCCGGCTATGGATTGATGACGGCGGCGCTGAGCAGTACGCTCTTCAACAGCGGCTATGGCTGCGGGCAGTGCTACGAGATCACTTGCACGCTATCGAAGCACTGCTACTTCGGAAAATCCGTGGTGGTGACCGCCACAAATCTTTGCCCGCCCAATTGGAGCAAGCCCTCGAACAATGGCGGCTGGTGCAATCCACCGCGCGTCCATTTCGACATGTCCAAGCCGGCATTCATGAAAATCGCGTTTTGGCGGGCGGGAATCATCCCAGTCTCCTATCGAAGGTGAGAGAAGAAAAACGCGCGAGATTTTAGGGTTCTTCAGCTAAAAGTTTGAAAACTTTCCTGCCAGGGTGCCATGCGCGCGATCCGGCGGCATGAATTTCAAGCTGAGCGGCAATCGCTGGTGGCTCATGGTGTTCATCACAAATGTGGGCGGCTCCGGCGATATCAAGGCGGTGTCGGTGAAGGGATCGAGGACGGGATGGATCGCCATGACGCGGAATTGGGGCGTAGGGTTCCAAGTTTTCAAGCAGCTCCAGGGCCAAAGTCTCTCGTTCATGGTCACTTGCTACAGTACCGGCAAGACCACCGTGCACAACAATGTGGCGCCCGCGAATTGGCAGCTCGGCAGCACCTATTCCGCCAAGCAATTGTGA

MRSVIACLLLCATLLVSVDHGRAASPKSLKKRATNAAKDIYNAGGWKQAFATFYGDETARETMGGACGYGNLYQSGYGLMTAALSSTLFNSGYGCGQCYEITCTLSKHCYFGKSVVVTATNLCPPNWSKPSNNGGWCNPPRVHFDMSKPAFMKIAFWRAGIIPVSYRRVPCARSGGMNFKLSGNRWWLMVFITNVGGSGDIKAVSVKGSRTGWIAMTRNWGVGFQVFKQLQGQSLSFMVTCYSTGKTTVHNNVAPANWQLGSTYSAKQL

SmEXPA6a [AB]

ATGATCTTAGCACTGGTTGTAAGCATTCTTCTGGCGATCCAAGCACTCGCAGTAGCAGCAGTTTATAGTTCTTCTTACTCCACTCCATCCAGATACAAAGGAGGCCTCTGGAGATATGCTCACGCGACCTTTTACGGAGAATACGACGCTCTCGAAACCATGGGCAAGTCACCAGATAGAACTCCTCCTCCTCGTCCCTGCTTTGTTTGTTCCTTCGAGCCTTTAGCACCATGATCCTGTTCTAGCTTCCATTTGAACTTGGCTAAACTTCACGTCTTTTCTTCCAGGAGGAGCTTGTGGATACGGGAACTTGTATTCGCAAGGATATGGCACTGATACCACTGCACTGAGCACCGTCCTCTTCAACAGCGGCTATGGTTGTGGAGGCTGCTACGAGATATCTTGTACACAGTCCAAGCATTGCTATCCCGGCTCCACCATCGTCACTGCCACCAATCTCTGTCCGCCAAACTGGTATAAACCATCCAACAATGGTGGCTGGTGTAATCCCCCGCGAATCCACTTTGACATGTCCAAGCCTGCCTTCTCGAAGATTGCCTACTGGAGAGCTGGAATAGTTCCTGTTCGCTACAGAAGGTAGGCGGCCCTGTGGAGTGAATCGACTGTCCCAAGCTCGAAGACTAATCTTGTCTGGCAGGGTGCCTTGTCGAAGGAAAGGTGGAATCAAGTTCGAGCTCAAAGGCAATCGCTGGTGGCTCATTGTCTTCGTGAGCAACGTTGGAGGGCCTGGCGACATCAAAAGAATGGCTGTTAAAGGATCCAAAACTGGCTGGTTACCAATGTCACGCAACTGGGGAGTTGGATTCCAGGTTTTCAAATCGCTCCATGGCCAAAGCCTTTCTTTCATGGTCACCTCCTTCACGACTGGAAAAACTGTGACGGCATACGACGTTGTCCCTGCTAACTGGAGAATCGGCCAAGCGTATTCTGGTGGTCAGATGGTATGA

MILALVVSILLAIQALAVAAVYSSSYSTPSRYKGGLWRYAHATFYGEYDALETMGKSPDRTPPPRGACGYGNLYSQGYGTDTTALSTVLFNSGYGCGGCYEISCTQSKHCYPGSTIVTATNLCPPNWYKPSNNGGWCNPPRIHFDMSKPAFSKIAYWRAGIVPVRYRRVPCRRKGGIKFELKGNRWWLIVFVSNVGGPGDIKRMAVKGSKTGWLPMSRNWGVGFQVFKSLHGQSLSFMVTSFTTGKTVTAYDVVPANWRIGQAYSGGQMV

SmEXPA6b [AB]

ATGATCTTAGCGCTGGTTGCAAGCATTCTTCTGGCGATCCAAGCACTCGCAGTAGCAGCAGTTTATAGTTCTTCTTACTCCACTCCATCCAGATACAAAGGAGGCCTCTGGAGATATGCTCACGCGACCTTTTACGGAGAATACGACGCTCTCGAAACCATGGGCAAGTCACCAGATAGAACTCCTCCTCCTCGTCCCTGCTTTGTTTGTTCCTTCGTTTAGCACCATGATCCTGTTCTAGCTTCCATTTGAACTTGGCTAAACTTCACGTCTTTTCTTCCAGGAGGAGCTTGTGGATACGGGAACCTGTACTCGCAAGGATACGGCACTGATACCACTGCACTGAGCACCGTCCTCTTCAACAGCGGCTATGGTTGTGGAGGCTGCTACGAGATATCTTGTACACAGTCCAAGCATTGCTATCCCGGCTCCACCATCGTCACTGCCACCAATCTCTGTCCGCCAAACTGGTATAAACCATCCAACAATGGTGGCTGGTGTAATCCCCCGCGAATCCACTTTGACATGTCCAAGCCTGCCTTCTCGAAGATTGCCTACTGGAGAGCCGGAATAGTTCCTGTTCGCTACAGAAGGTACGCGGCCCTGTGGAGTGAATCGACTGTCCCAAGCTCGAAGACTAACCTTGTCTTGCAGGGTGCCTTGTCGGAGGAAAGGTGGGATCAAGTTCGAGCTCAAAGGCAATCGCTGGTGGCTCATTGTCTTCGTGAGCAACGTTGGAGGGCCTGGCGACATCAAAAGAATGGCTGTTAAAGGATCCAAAACTGGCTGGTTACCAATGTCACGCAACTGGGGAGTTGGATTCCAGGTTTTCAAATCGCTCCATGGCCAAAGCCTTTCTTTCATGGTCACCTCCTTCACGACTGGAAAAACTGTGACGGCATACGACGTTGTCCCTGCTAACTGGAGAATCGGCCAAGCGTATTCTGGTGGTCAGATGGTATGA

MILALVVSILLAIQALAVAAVYSSSYSTPSRYKGGLWRYAHATFYGEYDALETMGKSPDRTPPPRGACGYGNLYSQGYGTDTTALSTVLFNSGYGCGGCYEISCTQSKHCYPGSTIVTATNLCPPNWYKPSNNGGWCNPPRIHFDMSKPAFSKIAYWRAGIVPVRYRRVPCRRKGGIKFELKGNRWWLIVFVSNVGGPGDIKRMAVKGSKTGWLPMSRNWGVGFQVFKSLHGQSLSFMVTSFTTGKTVTAYDVVPANWRIGQAYSGGQMV

SmEXPA7a [AB]

ATGGACTCCAAGCCGCTGCTCACTGCTCTCTCGATCTTCTTCCTGGTTTCTACAGCACTCCTTGCAAATGCCGATGCCAAGAAGCCGGGTGGCCACCACAAATATGGCAGAGGCGGGAGCCAAGGATCATGGCAATGGGGAGCCCACGCGACTTACTACGGTGGGAGCGACGCATCTGGGACAAACAGTACGATCCAATTCTCTCCACTCCTTGATCTTGATCTTATTCACGAGCTTATATACTCCCTCTCGACAAAATCTGGTTTTGCAGATGGAGCATGCGGCTATGGAAACCAGCTGAGCGCCGGCTATGGAACCATCACCACTGCTCTAAGCACCCCTCTCTTCCGCGGGGGCAATGTGTGTGGAGCGTGCTACCAAGTCCGGTGCTGGGGCGATCCAGCGTGCCTCCCGGGAAATCCCTCCGTCGTCGTCACCGCCACCAATCTCTGCCCGCCGGGGAGCAATGGCGGCTGGTGTGATCCGCCCAAGCCCCACTTTGATCTCTCGCAGCCGGCATTTTCTCGCATCGCCAGGATCCCCAACGGCCACGCCCAGATCCAGTACCGAAGGTAAACCAACTTTCTTCCTCGATCGCTTGGAGGAGCGATCTTGGTTTGATCGGATGGATTCTGATCCAATGCTCCATCGATCCAGGGTCAAGTGCCAGCGGCAGGGAGGGATTCGCTTCACGATCAATGGCCACACTTACTTCAATCTGGTGCTCGTCACGAACGTGGGCGGCATGGGCGACGTCGTGGGCGTGTCGATCAAGGGATCTAGCAGCGGCTGGCGATCCATGAGCCGGAACTGGGGCCAGAACTGGGAGGAGGGAAGCAATCTCAATGGCCAGGCGCTCTCCTTCCGCGTCACCACCAGCGATGGCAGGACCGTCACCGCCTACAATGTCGCGCCCGGGGACTGGCAATTCGGGAGAACTTACACTGGCAACACCGCCTCGCAGTACTACTGA

MDSKPLLTALSIFFLVSTALLANADAKKPGGHHKYGRGGSQGSWQWGAHATYYGGSDASGTNNGACGYGNQLSAGYGTITTALSTPLFRGGNVCGACYQVRCWGDPACLPGNPSVVVTATNLCPPGSNGGWCDPPKPHFDLSQPAFSRIARIPNGHAQIQYRRVKCQRQGGIRFTINGHTYFNLVLVTNVGGMGDVVGVSIKGSSSGWRSMSRNWGQNWEEGSNLNGQALSFRVTTSDGRTVTAYNVAPGDWQFGRTYTGNTASQYY

SmEXPA7b [AB]

ATGGACTCCAAGCCGCTGCTCACTGCTCTCTCGATCTTCTTCCTGGTTTCTACAGCACTCCTTGCAAATGCCGATGCCAAGAAGCCGGGTGGCCACCACAAATATGGCAGAGGCGGGAGCCAAGGATCATGGCAATGGGGAGCCCACGCGACTTACTACGGTGGGAGCGACGCATCTGGGACAAACAGTACGATCCAATTCTCTCCACTCCTTGATCTTGATCTTATTCACGAGCTTATATACTCCCTCTCGACAAAATCTGGTTTTGCAGATGGAGCTTGCGGCTATGGAAACCAGCTGAGCGCCGGCTATGGAACCATCACCACTGCTCTAAGCACCCCTCTCTTCCGCGGGGGCAATGTGTGTGGAGCCTGCTACCAAGTCCGGTGCTGGGGCGATCCAGCGTGCCTCCCGGGAAATCCCTCCGTCGTCGTCACCGCCACCAATCTCTGCCCACCGGGGAGCAATGGCGGCTGGTGTGATCCGCCCAAGCCCCACTTTGATCTCTCGCAGCCGGCATTTTCTCGCATCGCCAGGATCCCCAACGGCCACGCCCAGATCCAGTACCGAAGGTAAACCAACTTTCTTCCTCGATCGCTTGCAGGAGCGATCTTGGTTTGATCGGATGGATTCTGATCCAATGCTCCATCGATCCAGGGTCAAGTGCCAGCGGCAGGGAGGGATTCGCTTCACGATCAATGGCCACACTTACTTCAATCTGGTGCTCGTCACGAACGTGGGCGGCATGGGCGACGTCGTGGGCGTGTCGATCAAGGGATCTAGCAGCGGCTGGCGATCCATGAGCCGGAACTGGGGCCAGAACTGGGAGGAGGGAAGCAATCTCAATGGCCAGGCGCTCTCCTTCCGCGTCACCACCAGCGATGGCAGGACCGTCACCGCCTACAATGTCGCGCCCGGGGACTGGCAATTCGGGAGAACTTACACTGGCAACACCGCCTCGCAGTACTACTGA

MDSKPLLTALSIFFLVSTALLANADAKKPGGHHKYGRGGSQGSWQWGAHATYYGGSDASGTNNGACGYGNQLSAGYGTITTALSTPLFRGGNVCGACYQVRCWGDPACLPGNPSVVVTATNLCPPGSNGGWCDPPKPHFDLSQPAFSRIARIPNGHAQIQYRRVKCQRQGGIRFTINGHTYFNLVLVTNVGGMGDVVGVSIKGSSSGWRSMSRNWGQNWEEGSNLNGQALSFRVTTSDGRTVTAYNVAPGDWQFGRTYTGNTASQYY

SmEXPA8a [AB]

ATGCCAGGAGCTCGCGGCGATGGCGGATGGCTAGATGCTCACGCGACGTACTATGGAGGAAGCGATGCCTCGGGAACTAACAGTAAGCGATAAAAGAAACAGAGAATTCAATTCCCCTGTTCTTACCACGCCCCCGGTTTCATTCCTCTCCAGATGGAGCTTGCGGCTATGGAAACCAGCTGAGCGCCGGCTATGGCTACATCACCACTGCCCTCAGCACCCCGCTCTTCGAAAACGGCGACATTTGCGGCGCTTGCTATGAGATTCGCTGCGCGGGCACGGGATGCCTCCCGAGAAATCCTTCCACCGTCGTCACCGCTACCAATCTCTGTCCTCCGGGGAGCAATGGCGGCTGGTGCGATCCGCCCAAGCAGCACTTTGATCTCTCGCAGCCGGCCTTCTCCCAGATCGCCAGCATTCCCTATGGTCACGTCCTCCTCCAGTACCGCAGGTAAGCGAGACAGATAATCGATCCACCGCAAGAGCCCCTCCTAAAATCGCCTGTGATGTGCTGTGATGATCTAGAGTGCCGTGCCAAAGGCAAGGTGCCATCCACTACACGATCAATGGGCACACCTTCTTCAATCTGGTGCTGATCGAGAATGTAGGCGGCAGCGGCGATGTCGTGGGCGTGGAAATCAAAGGCTCAAACACCAATTGGATGCCCATGGCAAGGAATTGGGGCCAGAACTGGATGATCGGGGGCAACCTTGGCGGTCAGAGCCTCTCGTTTAGGGTCACCGGCAGCGATGGCCGCAAGGTCACCTCGCTCAACGTCGCGCCGGCGAACTGGCAATTTGGTAGAGCCTACAGTGGTGGCCAGTTCTAA

MPGARGDGGWLDAHATYYGGSDASGTNNGACGYGNQLSAGYGYITTALSTPLFENGDICGACYEIRCAGTGCLPRNPSTVVTATNLCPPGSNGGWCDPPKQHFDLSQPAFSQIASIPYGHVLLQYRRVPCQRQGAIHYTINGHTFFNLVLIENVGGSGDVVGVEIKGSNTNWMPMARNWGQNWMIGGNLGGQSLSFRVTGSDGRKVTSLNVAPANWQFGRAYSGGQF

SmEXPA8b [AB]

ATGCCAGGAGCTCGCGGCGATGGAGGATGGCTAGATGCTCACGCGACGTACTATGGAGGAAGCGATGCCTCGGGAACTAACAGTAAGCGATAAAAGAAACAGAGAATTCAATTCCCCTGTTCTTACCACGCCCCCGGTTTCATTCCTCTCCAGATGGAGCTTGCGGCTATGGAAACCAGCTGAGCGCCGGCTATGGCTACATCACCACTGCCCTCAGCACCCCGCTCTTCGAGGGCGGCGACATTTGCGGCGCTTGCTATGAGATTCGCTGCGCGGGCACGGGATGTCTCCCGAGAAATCCTTCCACCGTCGTCACCGCTACCAATCTCTGTCCTCCGGGGAGCAATGGCGGCTGGTGCGATCCGCCCAAGCAGCACTTTGATCTCTCCCAGCCGGCCTTCTCCCAGATCGCCAGCATTCCCTATGGTCACGTCCTCCTCCAGTACCGCAGGTAAGCGAGACAGATAATCGATCCATCGCAAGAAGAGCCCCTCCTAAAATCGCGTGTGATGTGCTGTGATGATCTAGAGTGCCGTGCCAAAGGCAAGGTGCCATCCACTACACGATCAATGGGCATACCTTCTTCAATCTGGTGCTGATCGAGAATGTAGGCGGCAGCGGCGATGTCGTGGGCGTGGAAATCAAAGGCTCAAACACCAATTGGATGCCCATGGCAAGGAATTGGGGCCAGAACTGGATGATCGGGGGCAACCTTGGCGGTCAGAGCCTCTCGTTTAGGGTCACCGGCAGCGATGGCCGCAAGGTCACCTCGCTCAACGTCGCGCCGGCGAACTGGCAATTTGGTAGAGCCTACAGTGGTGGCCAGTTCTAA

MPGARGDGGWLDAHATYYGGSDASGTNNGACGYGNQLSAGYGYITTALSTPLFEGGDICGACYEIRCAGTGCLPRNPSTVVTATNLCPPGSNGGWCDPPKQHFDLSQPAFSQIASIPYGHVLLQYRRVPCQRQGAIHYTINGHTFFNLVLIENVGGSGDVVGVEIKGSNTNWMPMARNWGQNWMIGGNLGGQSLSFRVTGSDGRKVTSLNVAPANWQFGRAYSGGQF

SmEXPA9a [AB]

ATGGATCTCCCACTTCTTTTTGCAGCAACCGTTGTGGTGCTGTTTTTGAGCCCTGAAGTTGGAGCTCAAGATTATGGATCATGGCAAGATGCTCACGCAACGTTTTATGGAGGAAGTGATGCCTCTGGAACCATGGGTACAATCATTTAGCAAGAAAAAAAGAAAAACAACCCATTCTAACTGGCATGTTTGCTTCAATGCAATGATCTTGTAAATTCAGGAGGAGCTTGTGGATATGGGAACCTCTACCTCCAAGGCTATGGAGTAAGCACAGCAGCACTGAGCACTGCCCTGTTCAATGAAGGCTGGTCGTGCGGCTCTTGCTTCGAATTGAAATGCAATGCCGAGGCCGACCCGGAATGGTGCTTGCCCGGCAACCCATCCATCGTGGTCACGGCCACAAACTTTTGCCCTCCAAACTTTGCTCTCCCAAGCGACAACGGAGGCTGGTGTAATCCCCCGCGCGAGCACTTTGATCTCTCACAGCCTGCATTTGAGCTGATTGCCAAGTACAGAGGTGGCATTGTTCCTGTTCAGTACAGAAGGTGAGCTCTCCTTCTCTAGAGAATGTTTTCTATAGTTTTCTACAAATTCATTCACTTCGTGTTTGCATTTGTTGTGCTACAGGGTACCTTGTGAAAGGGAAGGAGGCATTCATTTCTCTATCAATGGCCATGCCTATTTCATGCTGGTGCTGGTGTGGAATGTGGGTGGAGCCGGGGATGTCCACGCTGTGGCTGTCATGGGATCGAGAACCAGGAGGTGGCAGCCTTTGGTGAGAAACTGGGGGCAGAATTGGCAATCGCCAGATGTTCTTCTTGGCCAGTCCCTCTCTTTCATGGTAACCACGAGCAACGGTGACACTGTTACCGATTATGATGTTGCGCCTCAAGATTGGAAGTTTGGGCAAACGTTTGTAGGAAACAAAAATTGA

MDLPLLFAATVVVLFLSPEVGAQDYGSWQDAHATFYGGSDASGTMGGACGYGNLYLQGYGVSTAALSTALFNEGWSCGSCFELKCNAEADPEWCLPGNPSIVVTATNFCPPNFALPSDNGGWCNPPREHFDLSQPAFELIAKYRGGIVPVQYRRVPCEREGGIHFSINGHAYFMLVLVWNVGGAGDVHAVAVMGSRTRRWQPLVRNWGQNWQSPDVLLGQSLSFMVTTSNGDTVTDYDVAPQDWKFGQTFVGNKN

SmEXPA9b [AB]

ATGGATCTCCCATTTCTTTTTGCAGCAACCGTTGTGGTGCTGTTTTTGAGTCCTGAAGTTGGAGCTCAAGATTATGGATCATGGCAAGATGCTCACGCAACCTTTTATGGAGGAAGTGATGCCTCTGGAACCATGGGTACAATCATTTAGCAAGAAAAAAAGAAAAACAACCCATTCTAACTGGCATGTTTGCCTCAATGCAATGATCTTGTAAATTCAGGAGGAGCTTGTGGATATGGGAACCTCTACCTCCAAGGCTATGGGGTAAGCACTGCAGCACTGAGTACTCCCCTGTTCAACGAAGGCTGGTCGTGCGGCTCTTGCTTCGAATTGAAATGCAATGCCGAGGCCGACCCGGAATGGTGCTTGCCCGGCAACCCATCCATCGTGGTCACGGCCACAAACTTTTGCCCTCCAAACTTTGCTCTCCCAAGCGACGATGGAGGCTGGTGTAATCCCCCGCGCGAGCACTTTGATCTCTCACAGCCTGCATTTGAGCTGATTGCCAAGTACAGAGGCGGCATTGTTCCTGTTCAGTACAGAAGGTGAGCTCTCCTTCTCTAAAGATTGTTTTCTATAGTTTTCTACAAATTCATTCACTTCGTGTTTGCATTTGTTGTGCTACAGGGTACCTTGTGAAAAGGAAGGAGGCATTCATTTCTCTATCAATGGCCATGCCTATTTCATGCTGGTGCTGGTGTGGAATGTGGGTGGAGCCGGGGATGTCCACGCTGTGGCTGTCATGGGATCGAGAACCAGGAGGTGGCAGCCTTTGGTGAGAAACTGGGGGCAGAATTGGCAATCGCCAGAAGTTCTTCTTGGCCAGTCCCTCTCTTTCATGGTAACCACGAGCAACGGTGACACTATTACCGATTATGATGTTGCGCCTCAAGATTGGAAGTTTGGGCAAACGTTTGTAGGAAACAAAAATTGA

MDLPFLFAATVVVLFLSPEVGAQDYGSWQDAHATFYGGSDASGTMGGACGYGNLYLQGYGVSTAALSTPLFNEGWSCGSCFELKCNAEADPEWCLPGNPSIVVTATNFCPPNFALPSDDGGWCNPPREHFDLSQPAFELIAKYRGGIVPVQYRRVPCEKEGGIHFSINGHAYFMLVLVWNVGGAGDVHAVAVMGSRTRRWQPLVRNWGQNWQSPEVLLGQSLSFMVTTSNGDTITDYDVAPQDWKFGQTFVGNKN

SmEXPA10a [AB]

ATGGCTTCTTGTGTTCTTCTTCCTCTCTTCTTCCTCTCTCTCGCGGCAGCTCAAGAACGCGAGAAGCAAGCTACTCCAGAGGGCTGGACTTTAGCTCACGCAACGTACTATGGCGGCTCGGACGCGTCGGGAACAATGGGTAAAGTTTCGCTTCGTCTTTCCTGCCAAAGAGCCATCGATTCGTAAAGACTTCCTCCACAAGATCTCTGGGAAAGAAAGAACGAAAACAAAGGAGAGCGAAGAGAAGAACTGACCGTGATGTGCTTGTTTTGCGTGTGTCTTCTTGGCTCGATCTTCTCTCGCAGGAGGAGCTTGCGGCTATGGCAACATGTACCACGAAGGCTTTGGAGTGGAGACCACCGCTCTGAGCACCGTCTTGTTCCAGAACGGAGCTTCCTGTGGAGCTTGCTACGAGCTCAAGTGCCATCAGGATCCAAAGTGGTGCCGTCCCGGCAATCTCTCCATCACCGTCACCGCCACGAACTTCTGCCCTCCAAATCCCGCTCGCAAGAGCTACCGAGGAGGATGGTGCAACTACCCACAGCAACACTTCGATCTTTCCATGCCGGCCTTCGTTCACCTCGCGAACAGGACCGCAGGAATCATTCCAGTCATCTACACAAGGTAGCACACCATAAGTCATGAAATCACACTGACTTACGATGATCTTGTGTAGAGTCGAGTGCAAGAGGCAGGGTGGCATTCGTTTCACGATGCGTGGCAACAAATGGTTCATCTTGGTGATGATCTCCAACGTTGGTGGAGCGGGAGATGTCCGGAGCGTGGTTGTCAAAGGATCAAGATCTTGGACGCCGGCGACTCGCGCTTGGGGCCAGAACTGGCATATTTCCAACCGGTCGATGCTAGAGCAAGGCCTGTCTTTCGTTGTGAGTACCAGCGATGGCGAGAGCAGGATAGCTCTCGACGTAGTGCCGCGAAATTGGAAATTCGGACAAACCTTCACGACCGGCGCACAGTTCTAG

MASCVLLPLFFLSLAAAQEREKQATPEGWTLAHATYYGGSDASGTMGGACGYGNMYHEGFGVETTALSTVLFQNGASCGACYELKCHQDPKWCRPGNLSITVTATNFCPPNPARKSYRGGWCNYPQQHFDLSMPAFVHLANRTAGIIPVIYTRVECKRQGGIRFTMRGNKWFILVMISNVGGAGDVRSVVVKGSRSWTPATRAWGQNWHISNRSMLEQGLSFVVSTSDGESRIALDVVPRNWKFGQTFTTGAQF

SmEXPA10b [AB]

ATGGAGATGGCTTCTTGTGTTCTTCTTCCTCTCTTCTTCCTCTCTCTCGCAGTAGCGCAAGAACGCGAGAAGCAAGCTATTCCAGAGGGCTGGACTTTAGCTCACGCAACGTACTATGGCGGCTCGGACGCGTCGGGAACAATGGGTAAACTTTCGCTTCGTCTTTCCTGCCAAGAGCCATCGATTCGTAAAGACTTCCTCCACAAGATCTCTGGGAAAGAAAGAACGAAAACAAAGGAGAGCGAAGAGAAGAACTGACCGTGATGCGTGTGTCTTCTTGGCTCGATCTTCTCTCGCAGGAGGAGCTTGCGGCTATGGCAACATGTACCACGAAGGCTTTGGAGTGGAGACCACCGCTCTGAGCACCGTCTTGTTCCAGAACGGAGCTTCCTGTGGAGCTTGCTACGAGCTCAAGTGCCATCAGGATCCAAAGTGGTGCCGTCCCGGCAATCTCTCCATCACCGTCACCGCCACGAACTTCTGCCCTCCAAATCCCGCTCGCAAGAGCTACCGAGGAGGATGGTGCAACTATCCACAGCAACACTTCGATCTTTCCATGCCGGCCTTCGTTCACCTCGCGAACAGGACCGCAGGAATCATCCCAGTCATCTACACAAGGTAGCACACCATAAGTCATGAAATCACACTGACTTACGATGATCTTGTGTAGAGTCGAGTGCAAGAGGCAGGGTGGCATTCGTTTCACGATGCGTGGGAACAAATGGTTCATCTTGGTGATGATCTCCAACGTAGGTGGAGCTGGAGATGTCCGGAGCGTGGTTGTCAAAGGATCACGATCTCCGTGGACGCCGGCGACTCGCGCTTGGGGCCAGAACTGGCATATTTCCAACCGGTCGATGCTAGAGCAAGGCCTGTCTTTCGTTGTGAGTACCAGCGATGGCGAGAGTAGGATAGCTCTCGACGCAGTGCCGCAAAATTGGAAATTCGGACAAACCTTCACGACCGCTGCACAGTTCTAG

MASCVLLPLFFLSLAAAQEREKQATPEGWTLAHATYYGGSDASGTMGGACGYGNMYHEGFGVETTALSTVLFQNGASCGACYELKCHQDPKWCRPGNLSITVTATNFCPPNPARKSYRGGWCNYPQQHFDLSMPAFVHLANRTAGIIPVIYTRVECKRQGGIRFTMRGNKWFILVMISNVGGAGDVRSVVVKGSRSWTPATRAWGQNWHISNRSMLEQGLSFVVSTSDGESRIALDVVPRNWKFGQTFTTGAQF

SmEXPA11a [AB]

ATGGGACTGCTGGTGCTTTTTCTCGCCACCGGCGTCAGCTCCGCGCTGGCGCAGTGGGAGAGCGGCCACGCCACATTCTACGGAGGCAGCGACGCCGCTGGAACAATGGGTAAGAAGAGAGATCACACTACTCCCTCTCGGATCATTTCCATTCCAGCAACCCCACTCAGCAAAGATCTAGCATACAATCCACAAGAATTCTTTCTCGTGATCTAAAACGAATGCCATCTTGCCATGTCCAGGTGGAGCTTGCGGCTATGGCAATCTCTACAGCCAGGGCTACGGCACCAACAATGCGGCACTGAGCTCGGCGCTCTACAACAACGGGCTGAGCTGCGGCGCGTGCTTCGAGGTGAAATGTGACGCGGCGGCGGACCCGCAGTGGTGCATCCCGGGCCGATCGGTGACCGTCACCGCCACCAACTTCTGCCCGCCCGGCTCCTGGTGCAACGAGCCCCTCAAGCACTTCGACATGTCCCAGCCCGCCTGGGAGGAGATTGGAATCTACCGCGGTGGAATCATCCCCGTCTACTTCCGCAGGTATGTCGCTGCCTCTTCGTTCTATGTTCTTCCGAAGTTCTTGGAGCTCAACTCATCAGGATCTTCCAAGTTTTGCTGCTTTCTTCCGGGGTTTCTTCCGCGATCTCATGGGCGATTGCCTCTCTCGCAGGGTGAGCTGCGTGAGGAAAGGAGGGATCCACTTCACGGTAAACGGCCACGCCTACTTCAACCTCATCCTCATCACGAATGTGGGCGGTGCCGGGGACGTGCATGCCGTGTCCGTCAAGGGCTCCGGGACGGGATGGATCCCCATGAGCCGCAACTGGGGCCAGAACTGGCAGACCAACGCGCAGCTCGGCGGCCAGAGCCTCTCCTTCATGGTGACGGACAGTAGCGGCAAGACGGTCATCTCCAACAATGCCGCGCCTTCCAACTGGCAATACGGGCAGACCTTCGAGGGAGAACAGTTCTGA

MGLLVLFLATGVSSALAQWESGHATFYGGSDAAGTMGGACGYGNLYSQGYGTNNAALSSALYNNGLSCGACFEVKCDAAADPQWCIPGRSVTVTATNFCPPGSWCNEPLKHFDMSQPAWEEIGIYRGGIIPVYFRRVSCVRKGGIHFTVNGHAYFNLILITNVGGAGDVHAVSVKGSGTGWIPMSRNWGQNWQTNAQLGGQSLSFMVTDSSGKTVISNNAAPSNWQYGQTFEGEQF

SmEXPA11b [n’AB]

CAATCCTCTTCCCTCATTCATCTCCCCCTCCTCGGCCTTCTTTTCTCTTCTTTATCCTTGTCAATCAGGCTACGGTATACTCTTCCTTTGATTATAATTCCTCTAGATCTCTGGCCAGCGATCTTGATTGAGCGATCCGGCCGGGACAGAGAGCGAGAAAGATACAGAGGGCTTCCTCGCATCTATTTAGCCGTCAACCATCATCTGCTTCCACCGCTCCATCAAAAGAAATCTTGTTCCAAGAATGTAGGTGCTCTGTTTGTGACGCTCTGTTCTTGGCACTGTCATGTTCTTGGGCTGATCGATTCACTGGGGTTTCTCACCAACAGGGAGACGATGGGACTGCTGGTGCTTTTCCTCGCCACTGGCGTCAGCTCCGCGCTGGCGCAGTGGGAGAGCGGCCACGCCACATTCTACGGAGGCAGCGACGCCGCTGGAACAATGGGTAAGAAGAGAGATCACACTACTCCCTCTCGGATCATTTCCATTCCAGCAACCCCACTCAGCAAAGATCTAGCATGCAATCCACAAGAATTCTTTCTCGTGATCTAAAACGAATGCCATCTTGCCATGTCCAGGTGGAGCTTGCGGCTATGGCAATCTCTACAGCCAGGGCTACGGCACCAACAATGCGGCACTGAGCTCGGCGCTCTACAACAACGGGCTGAGCTGCGGCGCGTGCTTCGAGGTGAAATGTGACGCGGCGGCGGACCCGCAGTGGTGCATCCCAGGCCGATCCGTGACCGTCACCGCCACCAACTTCTGCCCGCCCGGCTCCTGGTGCAACGAGCCCCTCAAGCACTTCGACATGTCCCAGCCCGCCTGGGAGGAGATTGGAATCTACCGCGGTGGAATCATCCCCGTCTACTTCCGCAGGTATGCCGCTGCTTCTTCGCTCTATGTTCTTCCGAAGTTCTTGGAGCTCAACTCATCAAGATCTTCCAAGTTTTGCTGCTTTCTTCTGGGGTTTCTTCCGCGATCTCATGGGCGATTTGACTCTCTCGCAGGGTGAGCTGCGTGAGGAAAGGAGGGATCCATTTCACGGTAAACGGCCACGCCTACTTCAACCTCATCCTCATCACGAATGTGGGCGGTGCCGGGGACGTGCATGCCGTGTCCGTCAAGGGCTCCGGGACGGGATGGATCCCCATGAGCCGCAACTGGGGCCAGAACTGGCAGACCAACGCGCAGCTCGGCGGCCAGAGCCTCTCCTTCATGGTGACGGACAGTAGCGGCAAGACGGTCATCTCCAACAATGCCGCGCCTTCCAACTGGCAATACGGGCAGACCTTCGAGGGAGAACAGTTCTGAGGATGATCGACTCTACCGAAAAAGAGGAGGCGAATTGCGCTAGGGAATGGAATGAGCTGCTAGATGTTCATCGCCCACGTCGCCGTCTCGTCTCCCTCGTCGATCACGGTGGTGTTCTTCCGGTATTTTTTCTTTTCATGGTTAATTTTTTTCGTTTTTATTTTTCCTTCTTCTTGCTAAGAAGAGAGATAGAGATCTGAGAGCTAAGGTGGTAAACTTGAGGGGATGAGATGAGCAGGCGCTATGTAGAGCCACCCTCCCCGTTCATTGGTACAAGAACATGAGAATAAAGAGTTCCCGAAGCTGGCGATCTATAAGCCGGCTTCTATGAGAAACTT

MGLLVLFLATGVSSALAQWESGHATFYGGSDAAGTMGGACGYGNLYSQGYGTNNAALSSALYNNGLSCGACFEVKCDAAADPQWCIPGRSVTVTATNFCPPGSWCNEPLKHFDMSQPAWEEIGIYRGGIIPVYFRRVSCVRKGGIHFTVNGHAYFNLILITNVGGAGDVHAVSVKGSGTGWIPMSRNWGQNWQTNAQLGGQSLSFMVTDSSGKTVISNNAAPSNWQYGQTFEGEQF

SmEXPA12a [AB]

GGCAGTGGCAGCAGTAGCAGCAAAGGAAGGAAGATGGACTTCTTTTCGGCAGTTTTTCTTCCGGCTGCGCTGCTATTGTTCGTGGCTCTCAATCTCGCAGCTGCGCAACAATGGAGCAACGCTCATGCTACATTCTACGGGGGCAGCGATGCCTCGGGAACAATGGGTATGGCGAAGTGAAGTTCCTCTTGCGATTAGTTTTTGCTGACAAAAGTGGTCTTTTGTTTCTTGAAGGTGGTGCTTGCGGCTATGGAAACGTGCTTAGTGCTGGCTATGGAGTCAACACCGCGGCGTTGAGCACCGCCTTGTTCAATGGGGGAGCTACCTGCGGAGCTTGCTTCCAGATGCAGTGCGTCAACTCGAGATGGTGTCGTCCCGGAAAGTCGGTCACCGTCACCGCCACGAACTTTTGCCCCCCGAACAACGCCCTCCCAAGCGACAATGGGGGCTGGTGCAACACACCCAGGGAGCACTTCGATCTCTCCCAGCCAGTCTGGGAGCAAATGGCCATTTACCAAGGAGGGATCGTCCCAGTTCAGTACAGAAGGTAGACGCAAAAGCTTCCTTTTTCCTGTTTGTTTTTTCTCCAGGACTTTGATTTTGATGACGGAATTGATGATGTGGTGATGGTCTATCAGGGTCAAGTGCTACAAGCAAGGTGGGATCATCTTCACCATGAACGGCAACCCAAACTTCAACTTGGTTCTCATAAAAAACGTTGCCGGATGGGGGGATTTGAGAGCCGTGTCGATCAAAGGCTCCAACACCGGCTGGCTCCCAATGAAGAGGAACTGGGGATCGAATTGGGAATACCATGGAGTTCTAGTCGGCCAGTCCCTCTCCTTTCTGCTCACACCCAGCATGGGAGGATCTCTCATCTCGTACGATGTTTTTCCTCGCAACTGGCAGTTTGGACAGTCCTACTCGGGCCGGCAATTCAGCTAAAGCGAAGAGCCTTGCAGAACAAACGAAGAATAAGCGAGGACTTTTCTTAGTTGTGGGAGCGCTGCTCGAACTTTTCTCACGCGGAAAGAGTCTTCCAAACTCTCAGCTTTGCTGCGGTGCTTCAGGGGTACCCGCCACTGAGACTCCATGTTGATAGCAGTTGGATTTGATAAAAAAAAAAAAAACAATGGCC

MDFFSAVFLPAALLLFVALNLAAAQQWSNAHATFYGGSDASGTMGGACGYGNVLSAGYGVNTAALSTALFNGGATCGACFQMQCVNSRWCRPGKSVTVTATNFCPPNNALPSDNGGWCNTPREHFDLSQPVWEQMAIYQGGIVPVQYRRVKCYKQGGIIFTMNGNPNFNLVLIKNVAGWGDLRAVSIKGSNTGWLPMKRNWGSNWEYHGVLVGQSLSFLLTPSMGGSLISYDVFPRNWQFGQSYSGRQFS

SmEXPA12b [AB]

ATGGACTTCTTTTCGGCAGTTTTTCTTCCGGCTGCGCTGCTATTGTTCGTGGCTCTCAATCTCGCAGCTGCGCAACAATGGAGCAATGCTCATGCTACATTCTACGGGGGCAGCGATGCCTCGGGAACAATGGGTATGGCGAAGTGAAGTTCCTTTTGCGATTAGTTTTTGCTGACAAAAGTGGTCTTTTGTTTCTTGAAGGTGGTGCTTGCGGCTATGGAAACGTGCTTAGTGCTGGCTATGGAGTCAACACCGCGGCGTTGAGCACCGCCTTGTTCAATGGGGGAGCTACCTGCGGAGCTTGCTTCCAGATGCAGTGCGTCAACTCGAGATGGTGTCGTCCCGGAAAGTCGGTCACCGTCACCGCCACGAACTTTTGCCCCCCGAACAACGCCCTCTCAAGCGACAATGGAGGCTGGTGCAACACACCCAGGGAGCACTTCGATCTCTCCCAGCCAGTCTGGGAGCAAATGGCCATTTACCAAGGAGGGATCGTCCCAGTTCAGTACAGAAGGTACACGCAAAAGCTTCCTTTTCCCTGTTTGTTTTTTCTCCAGGACTTTGATTTTGATGACGGAATTGATGATGTGGTGATGGTCTATCAGGGTCAAGTGCTACAAGCAAGGTGGGATCATCTTCACCATGAACGGTAACCCAAACTTCAACTTGGTTCTCATAAAAAACGTTGCCGGATGGGGGGATTTGAGAGCCGTGTCGATCAAAGGCTCCAACACCGGCTGGCTCCCAATGAAGAGGAACTGGGGATCGAATTGGGAATACCATGGAGTTCTAGTCGGCCAGTCCCTCTCCTTTCTGCTCACACCCAGCATGGGAGGATCTCTCATCTCGTACGATGTTTTTCCTCGCAACTGGCAGTTTGGACAGTCCTACTCGGGCCGGCAATTCAGCTAA

MDFFSAVFLPAALLLFVALNLAAAQQWSNAHATFYGGSDASGTMGGACGYGNVLSAGYGVNTAALSTALFNGGATCGACFQMQCVNSRWCRPGKSVTVTATNFCPPNNALSSDNGGWCNTPREHFDLSQPVWEQMAIYQGGIVPVQYRRVKCYKQGGIIFTMNGNPNFNLVLIKNVAGWGDLRAVSIKGSNTGWLPMKRNWGSNWEYHGVLVGQSLSFLLTPSMGGSLISYDVFPRNWQFGQSYSGRQFS

SmEXPA13a [AB]

ATGGAAATTTTTTCCCGGGTCACTGGCCTTCTTCTCTCCATTTTCTTCCTTGGAGCTCGCCAAATCCGTGGCGATGAAGGATGGACCGATGGAGCTCACGCAACTTACTATGGAGGAAGTGATGCATCAGGAACAAATAGTAAGTTCTTACGTGAAGTCTCAACAGTAAGTTCCAATGATTTTGATCTCTCGTTTTTTTCTTCCGTGATAGATGGCGCGTGTGGCTATGGAAACCAGCTGAGCGCTGGCTATGGAGTTCTCACCACTGCTTTGAGCGCTCCACTGTTTAACGATGGTCACGTTTGCGGAGCTTGCTTCGAAGTGAAGTGTTCTTGGGGCGACTCTGGCTGCCTCGCCGGGAATCCTTCCATTGTTGTCACTGCCACGAATCTGTGCCCGCAAGGAAGCAATGGAGGATGGTGTGATTCTCCCAAGCAACACTTTGATCTCGCGCAGCCGGCGTTCGCTCTCATCGCCGTGACTCTCAATGGTCACGTCCCAATCCAATACCGGAGGTAGTCAATCCATCCTCTCCATGGTTCTTGCCAGATCTCTCTCGAATTTGTCACTGACTTCTCTCGATTTCTTCCAGAGTGAGCTGCAAGCGCGATGGAGGCCTTCGATTCACGATCAATGGTCACGTCTACTTCAATCTGGTGCTCATCGAGAACGTTGGCGGCACCGGGGACGTGAGCGCGGTGTCGATCAAGGGATCCAAGACCGGGTGGCGGCCGATGACGAGGAACTGGGGCCAAAACTGGCAAGATGGCGGGGATCTCACTGGCCAGAGCTTGTCCTTCGAGGTCACCACCAGCGATGGAAGCAAGATCACGGCCTACGATGTCGCTCCAGACTACTGGCAGTTTGGACAGACCTTTAGCGGCGGCCAGTTCTAG

MEIFSRVTGLLLSIFFLGARQIRGDEGWTDGAHATYYGGSDASGTNNGACGYGNQLSAGYGVLTTALSAPLFNDGHVCGACFEVKCSWGDSGCLAGNPSIVVTATNLCPQGSNGGWCDSPKQHFDLAQPAFALIAVTLNGHVPIQYRRVSCKRDGGLRFTINGHVYFNLVLIENVGGTGDVSAVSIKGSKTGWRPMTRNWGQNWQDGGDLTGQSLSFEVTTSDGSKITAYDVAPDYWQFGQTFSGGQF

SmEXPA13b [AB]

ATGGAAATTTTTTCCCGGGCCACTGGCCTTCTTCTCTCCATTTTCTTCCTTGGAGCTCGCCAAATCCGTGGCGATGAAGGATGGACCGACGGAGCTCACGCAACTTACTACGGAGGAAGTGATGCATCAGGAACAAACAGTAAGTTCTTACGAACAGTAGGTTCCAATGATTTTGATCTCTCGTTTTTTACTTCCGTGATAGATGGCGCGTGTGGCTATGGAAACCAGCTGAGCGCTGGCTATGGAGTTCTCACCACTGCTTTGAGCGCCCCACTGTTTAACGATGGTCACGTTTGCGGAGCTTGCTTCGAAGTGAGGTGTTCTTGGGGCGACTCTGGCTGCCTCGCCGGGAATCCTTCCATTGTTGTCACTGCCACGAATCTGTGCCCGCAAGGAAGCAATGGAGGATGGTGTGATTCTCCCAAGCAACACTTTGATCTCGCGCAGCCGGCATTCGCTCTCATCGCCGTGATTCTCAATGGTCACGTCCCAATCCAATACCGGAGGTAGTCAATCCATCCTCTCCATGATTCTTGCGAGATCTCTCTCGAATTTGTCACTGACTTGTCTCGATTTCTTCCAGAGTGAGCTGCAAGCGCGATGGAGGCCTTCGATTCACGATCAATGGTCACGTCTACTTCAATCTGGTGCTCATCGAGAACGTTGGCGGCACCGGGGACGTGAGCGCGGTGTCGATCAAGGGATCCAAGACCGGGTGGCGGCCGATGACGAGGAACTGGGGCCAAAACTGGCAAGATGGCGGGGATCTCACTGGCCAGAGCTTGTCCTTCGAGGTCACCACCAGCGATGGAAACAAGATCACGGCCTACGATGTCGCTCCAGACTACTGGCAGTTTGGACAGACCTTTAGCGGCGGCCAGTTCTAG

MEIFSRATGLLLSIFFLGARQIRGDEGWTDGAHATYYGGSDASGTNNGACGYGNQLSAGYGVLTTALSAPLFNDGHVCGACFEVRCSWGDSGCLAGNPSIVVTATNLCPQGSNGGWCDSPKQHFDLAQPAFALIAVILNGHVPIQYRRVSCKRDGGLRFTINGHVYFNLVLIENVGGTGDVSAVSIKGSKTGWRPMTRNWGQNWQDGGDLTGQSLSFEVTTSDGNKITAYDVAPDYWQFGQTFSGGQF

SmEXPA14a [AB]

ATGCTCCTGCGGCGAGTGCATGGAGATGGAGGCTGGCTGGACGGAGCGCACGCAACTTACTACGGAGGGAGCGATGCGTCCGGGACAAACAGTGAGATCCCTTGAAAACCTCCCCTGTTCTATTCTCTTCTGGTTTCTCATTCGCATTCTCTTTCCTCTCCTTGCCAGATGGAGCTTGCGGCTATGGAAACCAGCTGAGCGCCGGCTACGGCTACATCACCACCGCCCTGAGCACTCCGCTCTTCGAAAACGGCGACATCTGCGGAGCGTGCTTCGAGATCCGGTGCGCCGGTGGCGCGGGATGCCTGCCGGGCAATCCCTCCACCGTCGTCACCGCCACGAATCTCTGCCCCCCAGGGAGCAACGGTGGCTGGTGCGACCCGCCCAAGCCCCACTTCGACCTCTCGCAGCCCGCATTCAGCAGGATCGCGAGCATCCCGAATGGCCACGTTCAGCTCCAATACAGGAGGTAAGAGAGAGCGCTCACTCACTGCTTAACTCACTCACTCTCACTCAGCTGACTAAATGACTTGAGTCGTTGACTAGTTCACCTTTTGACTTGACGATGACTATCAATGCAGAGTTGCGTGCGATAGGCAAGGGGGGATTCGCTTCACGGTCAACGGCCACACCTTCTTCAACCTTGTCCTGGTGGAGAACGTTGGCGGTAGCGGCGACGTGGTGGCAGTGGAGGTGAAGGGCTCCGCCACCGGGTGGCGGCAAATGCAGAGGAACTGGGGCCAGAACTGGCAAGACATGGGCGACCTTAACGGCCAGGCGCTCTCCTTTCGGGTCACCGGGAGCGATGGGAAGGTGGTCACGTCCATGAATGTGGCTCCCGCGGACTGGCAGTTTGGGAGAACTTACAGCGGCGGCCAGTTCTGA

MLLRRVHGDGGWLDGAHATYYGGSDASGTNNGACGYGNQLSAGYGYITTALSTPLFENGDICGACFEIRCAGGAGCLPGNPSTVVTATNLCPPGSNGGWCDPPKPHFDLSQPAFSRIASIPNGHVQLQYRRVACDRQGGIRFTVNGHTFFNLVLVENVGGSGDVVAVEVKGSATGWRQMQRNWGQNWQDMGDLNGQALSFRVTGSDGKVVTSMNVAPADWQFGRTYSGGQF

SmEXPA14b [AB]

ATGACGAACAAGTGGGCTGCTATGATGAAAGTCTGTGCGGTGTTTGCCGCTGCCGCGCTCATGCTCGTGCGGCGAGTGCATGGAGATGGAGGCTGGCTGGACGGAGCGCACGCAACTTACTACGGAGGGAGCGATGCGTCCGGGACAAACAGTGAGATCCCTTGAAAACCTCCCCTGTTCTATTCTCTTCTGGTTTCTCATTCGCATTCTCTTTCCTCTCCTTGCCAGATGGAGCTTGCGGCTATGGAAACCAGCTGAGCGCCGGCTACGGCTACATCACCACCGCCCTGAGCACTCCGCTCTTCGAAAACGGCGACATCTGCGGAGCGTGCTTCGAGATCCGGTGCGCCGGTGGCGCGGGATGCCTGCCGGGCAATCCCTCCACCGTCGTCACCGCCACGAATCTCTGCCCCCCGGGGAGCAACGGCGGCTGGTGCGACCCGCCCAAGCCCCACTTCGACCTCTCCCAGCCCGCATTCAGCAGGATCGCGAGCATCCCAAATGGCCACGTTCAGCTCCAATACAGGAGGTAAGAGAGAGCGCTCACTCACTGCTTAACTCACTCACTCTCACTCAGTTGACTAAATGACTTGAGTCGTTGACTAGTTCACCTTTTGACTTGACGATGACTAAAAGCTTCAATGCAGAGTTGCGTGCGATAGGCAAGGAGGGATTCGCTTCACGGTCAACGGCCACACCTTCTTCAACCTTGTCCTAGTGGAGAACGTTGGCGGTAGCGGCGACGTGGTGGCAGTGGAGGTGAAGGGCTCCGCCACCGGGTGGCGGCAAATGCAGAGGAACTGGGGCCAGAACTGGCAAGACATGGGCGACCTTAACGGCCAGGCGCTCTCCTTCCGGGTCACCGGGACCGATGGGAAGGTGGTCACGTCCATGAATGTGGCTCCCGCGGACTGGCAGTTTGGCAGAACTTACAGCGGCGGCCAGTTCTGA

MTNKWAAMMKVCAVFAAAALMLVRRVHGDGGWLDGAHATYYGGSDASGTNNGACGYGNQLSAGYGYITTALSTPLFENGDICGACFEIRCAGGAGCLPGNPSTVVTATNLCPPGSNGGWCDPPKPHFDLSQPAFSRIASIPNGHVQLQYRRVACDRQGGIRFTVNGHTFFNLVLVENVGGSGDVVAVEVKGSATGWRQMQRNWGQNWQDMGDLNGQALSFRVTGTDGKVVTSMNVAPADWQFGRTYSGGQF

SmEXPA15 [AB]

ATGGACTCCAAGCCGCTGCTCACTGCTCTCTCGATCTTCTTCCTGGTTTCTACAGCACTCCTTGCAAATGCCGATGCCAAGAAGCCGGGTGGCCACCACAAATATGGCAGAGGCGGGAGCCAAGGATCATGGCAATGGGGAGCCCACGCGACTTACTACGGTGGGAGCGACGCATCTGGGACAAACAGTACGATCCAATTCTCTCCACTCCTTGATCTTGATCTTATTCACGAGCTTATATACTCCCTCTCGACAAAATCTGGTTTTGCAGATGGAGCTTGCGGCTATGGAAACCAGCTGAGCGCCGGCTATGGAACCATCACCACTGCTCTAAGCACCCCTCTCTTCCGCGGGGGCAATGTGTGTGGAGCCTGCTACCAAGTCCGGTGCTGGGGCGATCCAGCGTGCCTCCCGGGAAATCCCTCCGTCGTCGTCACCGCCACCAATCTCTGCCCACCGGGGAGCAATGGCGGCTGGTGTGATCCGCCCAAGCCCCACTTTGATCTCTCGCAGCCGGCATTTTCTCGCATCGCCAGGATCCCCAACGGCCACGCCCAGATCCAGTACCGAAGGTAAACCAACTTTCTTCCTCGATCGCTTGCAGGAGCGATCTTGGTTTGATCGGATGGATTCTGATCCAATGCTCCATCGATCCAGGGTCAAGTGCCAGCGGCAGGGAGGGATTCGCTTCACGATCAATGGCCACACTTACTTCAATCTGGTGCTCGTCACGAACGTGGGCGGCATGGGCGACGTCGTGGGCGTGTCGATCAAGGGATCTAGCAGCGGCTGGCGATCCATGAGCCGGAACTGGGGCCAGAACTGGGAGGAGGGAAGCAATCTCAATGGCCAGGCGCTCTCCTTCCGCGTCACCACCAGCGATGGCAGGACCGTCACCGCCTACAATGTCGCGCCCGGGGACTGGCAATTCGGGAGAACTTACACTGGCAACACCGCCTCGCAGTACTACTGA

MDSKPLLTALSIFFLVSTALLANADAKKPGGHHKYGRGGSQGSWQWGAHATYYGGSDASGTNNGACGYGNQLSAGYGTITTALSTPLFRGGNVCGACYQVRCWGDPACLPGNPSVVVTATNLCPPGSNGGWCDPPKPHFDLSQPAFSRIARIPNGHAQIQYRRVKCQRQGGIRFTINGHTYFNLVLVTNVGGMGDVVGVSIKGSSSGWRSMSRNWGQNWEEGSNLNGQALSFRVTTSDGRTVTAYNVAPGDWQFGRTYTGNTASQYY

Pseudogene

GCTAACGATAATAGCGGCTGGTGCAATCCTCCCTTGGAGCACTTCGACATGGCCCAGCCAGCATGGGAACAGATTGGCATCTACCAAGGAGGAATTGTTCCCATCCAATACAGAAGGTACTCTTCACTTCTTTCATACTTTTCTCTCTTTTCTTCTTTCTCTTTCTCACGCACTATTCTTTATTGATTTCTCTTTTCTTCTATATTGCAGGGTGAGCTAGTATGGATACAAACTGTGAAAGCATGGCCTATGAATGCTTGGCTGCCACAAGCCAGTTATCCTTGTGGTAACTTTTCTGACACCTCTAACTTCAAATTCGGAAACACTAAAGGATTGTATGGATTACCATTTTTTATCCTTGGCCCCAAACTAATATTCATCAATGGTTTGATACTGATGGCTTACCATGCCTTGCCTATCCTCTAAGGAGCATAATCTCTTCAATTACGCTCAACGGGAACAAATACTTCATGCTGGTGCTCATGAGCAACGTTGGAGGGGCTGGGGACGTGTGAGCGGTGTCGATCAAGGGGCTGAGCAGCGACTGACAGCCAATGTCAAGGAACTGGGGCCAAAACTGGCAGAGCGATAGCAGGCTCATCGGGCAGAGCCTCTCGTTCTGCGTCGTGACGAGTGACAATCGACTGGTCACGTCACTCAATGTCGCACAGGCGGGGTGGAGCTTTGGCCAGACTTTTAATGGAGAACAGTTTTGA

ANDNSGWCNPPLEHFDMAQPAWEQIGIYQGGIVPIQYRSIISSITLNGNKYFMLVLMSNVGGAGDPMSRNWGQNWQSDSRLIGQSLSFCVVTSDNRLVTSLNVAQAGWSFGQTFNGEQF

SmEXPB1a [ACBF]

ATGATCAAATGGGAACCAGCCACTGCAACATGGTATGGAAGTCCCAATGGAGCTGGAACTGATGGTACAATGCAATCACACCCAATAACAAATTTTTTGAGAATTGTACAATCATCAGGAAATGATTTTTAGGAAAGAACTAGAAGAGTGTGTCGAGTTAGAATGTTATAACTCGTGATTTTTCTCAATGCTTTCTTTCTTTTCTTTTTTTGAAGGTGGAGCTTGTGGATATGGAAGCCTGCCCAACACACCCTATGGCTCAGATGTAGGTGCTGGGAGCCCAATTCTTTTCATGAATGGAATTGGCTGTGGAACTTGTTTTGAGGTAATCTATCATGAGTTTCTTTGCTCTTCTTTCTTTCTCTGTTTTTTCAAGTTTCCCTGTAGTATCAATCTATTGAATGCATCAAATCAATCGATCATTCTCATGACAATGTGGCCATCCAAATCCATGTAGGTGAAGTGCGTCGACGGGCAGCTCTGCTCGCCTCAGCCCGTCAACGTGGTCATCACTGACGAGTGTCCCGGAGGCTACTGCTCGGGAGGAAGAACACACTTTGATCTCAGTGGCACTGCCTTTGGAAAGATGGCCAGCGGCAGCGCAAACATCCAGCACCTCCTCGCCGCGGGAGTTCTCAATGTTCTCTACAGACGGTAAGCCTGACTCTGAGATTATTCCTGGAATATTTGAGGAATCTCTTCCCAATTTCAGTGCTCCATGCATCTACAAGAGCCAGGGCGTCGTGTTCCAGGTCGCCGATGGCTCCACACCCTTTTGGTTTGAGACAGTTATCAGATATCTGGATGGTCCGGGAGATCTTGCAACAGTCGAGCTCCAACAGGTAATCACCTTTTGCAAAGAAATTCCAAAGTTTTTGGTGAAGAGATTGACAACTAATGGAACTTTTGTTACTATATAGTTTGGGTCTTCTGCTTGGCAGCCGATGAGCCAAGTTTGGGGGGCAAACTGGTGCCTGAACGCAGGTGGCGGCACGCCTTTGAGAGCTCCATTTTCGATTCGGCTCACGGCACTTCAAACCGGAGAGAAGATCATCGCCCACAATGTCATTCCTGCGAATTGGGCACCCCAACACAGCTATTCCACTGGTGTCAACTTTGACACCCGCAACTATTGA

MIKWEPATATWYGSPNGAGTDGGACGYGSLPNTPYGSDVGAGSPILFMNGIGCGTCFEVKCVDGQLCSPQPVNVVITDECPGGYCSGGRTHFDLSGTAFGKMASGSANIQHLLAAGVLNVLYRRAPCIYKSQGVVFQVADGSTPFWFETVIRYLDGPGDLATVELQQFGSSAWQPMSQVWGANWCLNAGGGTPLRAPFSIRLTALQTGEKIIAHNVIPANWAPQHSYSTGVNFDTRNY

SmEXPB1b [ABCF]

ATGATCAAATGGGAACCAGCCACTGCAACATGGTATGGAAGTCCCAATGGAGCTGGAACTGATGGTACAATGCAATCACACCCAATAACAAATTTATTGAGAATTGTACAATCATCAGGAAATGATTTCTAAGAAAGAACTAGAAGAGTGTGTCGAGTTAGAATGTTATAACTCGTGATTTTTCTCAATGCTTTCTTTCTTTTCTTTTTTTGAAGGTGGAGCTTGTGGATATGGAAGCCTGCCCAACACACCCTATGGCTCAGATGTAGGTGCTGGGAGCCCAATTCTTTTCATGAATGGAATTGGCTGTGGAACTTGTTTTGAGGTAATCTATCATGAGTTTCTTCGCTCTTCTTTCTTTCTCTGTTTTTTCAAGTTTCCCTGTAGTATCAATCTATTGAATGCATCAAATCAATCGATCATTCTCATGACAATGTGGCTATCCAAATCCATGTAGGTGAAGTGCGTCGACGGGCAGCTCTGCTCGCCTCAGCCCGTCAACGTGGTCATCACTGACGAGTGTCCCGGAGGCTACTGCTCGGGAGGAAGAACACACTTTGATCTCAGTGGCACTGCCTTTGGAAAGATGGCCAGCGGCAGCGCAAACATCCAGCACCTCCTCGCCGCGGGAGTTCTCAATGTTCTCTACAGACGGTAAGCCTGACTCTGAGATTATTCCTGGAATATTTGAGGAATCTCTTCCCAATTTCAGTGCTCCATGCATCTACAAGAGCCAGGGCGTCGTGTTCCAGGTCGCCGATGGCTCCACACCCTTTTGGTTTGAGACAGTTATCAGATATCTGGATGGTCCGGGAGATCTCGCGACAGTCGAGCTCCAACAGGTAATCACCTTTTGCAAAGAAATTCCAAAGTTTTTGGCAAAGAGATTGAGAACTAATGGAACTTTTGTAGTTTGGGTCTTCTGCTTGGCAGCCGATGAGCCAAGTTTGGGGGGCAAACTGGTGCCTGAACGCAGGTGGCGGCACGCCTTTGAGAGCTCCATTTTCGATTCGGCTCACGGCACTTCAAACCGGAGAGAAGATTATCGCCCACAATGTCATTCCTGCGAATTGGGCACCCCAACACAGCTATTCCACTGGTGTCAACTTTGACACGCGCAATTATTGACGAGAGTACTCAGGGGCTTCTGTAGTGTGTGAAAAAATGCCACACTCGGGCCAATGAAAATTTAAATAAAAGTTTGAAAAGAAGTGTC

MIKWEPATATWYGSPNGAGTDGGACGYGSLPNTPYGSDVGAGSPILFMNGIGCGTCFEVKCVDGQLCSPQPVNVVITDECPGGYCSGGRTHFDLSGTAFGKMASGSANIQHLLAAGVLNVLYRRAPCIYKSQGVVFQVADGSTPFWFETVIRYLDGPGDLATVELQQFGSSAWQPMSQVWGANWCLNAGGGTPLRAPFSIRLTALQTGEKIIAHNVIPANWAPQHSYSTGVNFDTRNY

SmEXPB2a [ACBF]

ATGGATTCAGCGAGTTTGTGGAGGCTCATCGTCTTGCTGCTCGCAGCAGTGGATGTTGCTCGTTGCAGATTCCATGCGTATGCACGAGGCTGGCAATTGGCAACCGCGACTTGGTATGGAAGTCCCAGCGGTGCTGGAACTGATGGTATGCGAGGAAATCTCTCTCCAATCCTTCTTCTTCTCTTTCTTGCAGTGATCAATGTCCTTTCTCTCTTGCATTTCCAGGAGGAGCTTGTGGCTATGGTGAACTCCCGAACACTCCCTACGGCCTCGATGTTGGTGCTGGGAGCCCCGTCCTGTTCAAGAATGGCAGAGGCTGTGGTGCATGCTACAAGGTATGAGAGATTTGGATCTCCTTCTCAAGCTCTTCCAAAAGAATTCATGTTCTTCTTTTTCGGCTTGGATTGCACAGGTGAGGTGCTTGCAGCAGCAGCTCTGCTCCGGCAAAGCCGTGACGGTGGTGATCACAGACGAGTGTCCCGGTGGCTACTGCGCGTTTGGGAGGACGCATTTCGACCTCAGTGGCACGGCATTTGGAAGAATGGCGATGGCCGGAAGAACAAACCAGCTGTTGGGATCAGGAGTTACGCAAGTCCTCTACAAGAGGTAATAGTTAAAAGGAGTTCATGGCCAACTTCTCCTCACCTTTAACACATTTTGCAGGGTTGATTGTAACTATGGCTCCAGGCCCATGGAATTCCAAGTCAACGAAGGATCCACACCATTTTGGTTGTCCATTCTCGTTCGCTACGCAGCGGGGCCTGGAGATTTGGGTCATGTCGAGCTCATGCAGGTAGATAAACATGCCTTTCTTCACTTCTTTTTCTTTTTCTCTCTCTCTTAGCTTACACTGACGAAACGAATTGTTTGTGCTCTCTCTCTCTCTCTCCACACACACACACACACACACAACAGGCTGGGTCTCGAGTTTGGCAGCCAATGACTCAGGTGTGGGGCGCAACATGGTGTTTCAATGGCGGTCCTCTGAGGGGCCCTTTCTCGTTTCGAGTCACGACTCTGTCAACCTCGGAAACAGTCGTGGCTCGAAACGTGATTCCAAGAAACTGGGCTTCCAACACTTGCTATCGCTCCAGGGTCAACTTTAGATGA

MDSASLWRLIVLLLAAVDVARCRFHAYARGWQLATATWYGSPSGAGTDGGACGYGELPNTPYGLDVGAGSPVLFKNGRGCGACYKVRCLQQQLCSGKAVTVVITDECPGGYCAFGRTHFDLSGTAFGRMAMAGRTNQLLGSGVTQVLYKRVDCNYGSRPMEFQVNEGSTPFWLSILVRYAAGPGDLGHVELMQAGSRVWQPMTQVWGATWCFNGGPLRGPFSFRVTTLSTSETVVARNVIPRNWASNTCYRSRVNFR

SmEXPB2b [ACBF]

ATGGATTCAGCGAGTTTGTGGAGGCTCATCGTCTTGCTGCTCGCAGCAGTGGATGTTGCTCGTTGCAGATTCCATGCGTATGCACGAGGCTGGCAATTGGCAACCGCGACTTGGTATGGAAGTCCCAGCGGTGCTGGAACTGATGGTATGCGAGGAAATCTCTCTCCAATCATTCTTCTTCTCTTTCTTGCAGTGATCAATGCCTTCGTTTTCCTTTGTCTCTTGCATTTCCAGGAGGAGCTTGTGGCTATGGTGAACTCCCGAACACTCCCTACGGCCTCGATGTTGGTGCTGGGAGCCCCGTCCTGTTCAAGAATGGCAGAGGCTGTGGTGCATGCTACAAGGTATGAGAGATTTGGATTTCCTTCCCAACCCCTTCCAAAAGAATTCATGTTCTTCGTTTTTGGCTTGGATTGCACAGGTGAGGTGCTTGCAGCAGCAGCTCTGCTCCGGCAAAGCCGTGACGGTGGTGATCACAGACGAGTGTCCCGGTGGCTACTGCGCGTTTGGGAGGACGCATTTCGACCTCAGTGGCACGGCATTTGGAAGAATGGCGATGGCCGGAAGAACAAACCAGCTGTTGGGATCAGGAGTTATGCAAGTCCTCTACAAGAGGTAATAGTTAAAAAGAGTTCATGGCCAACTTCTCCTGACCCTTTAACAAATTTTGCAGGGTCGATTGTAACTATGGCTCCAGGCCCATGGAATTCCAAGTCAACGAAGGATCCACACCATTTTGGTTGTCCATTCTCGTTCGCTACGCAGCGGGACCTGGAGATTTGGGTCATGTCGAGCTCATGCAGGTAGATAAACATGCCTTTCTTCACTTCTTTTTCTTTCTCTCTCTCTCTTAGCTTACACTGACGAAACGAATTGTTTGTGCTCTCTCTCTCTCTCTCTCTCTCTCTCTCTCTCTCTCGCCACACACACACACACACAACAGGCTGGGTCTCGAGTTTGGCAGCCAATGACTCAGGTGTGGGGCGCAACATGGTGTTTCAATGGCGGTCCTCTGAGGGGCCCTTTCTCGTTTCGAGTCACGACTCTGTCAACCTCGGAAACAGTCGTGGCTCGAAACGTGATTCCAAGGAACTGGGCTTCCAACACTTGCTATCGCTCCAGGGTCAACTTTAGATGA

MDSASLWRLIVLLLAAVDVARCRFHAYARGWQLATATWYGSPSGAGTDGGACGYGELPNTPYGLDVGAGSPVLFKNGRGCGACYKVRCLQQQLCSGKAVTVVITDECPGGYCAFGRTHFDLSGTAFGRMAMAGRTNQLLGSGVTQVLYKRVDCNYGSRPMEFQVNEGSTPFWLSILVRYAAGPGDLGHVELMQAGSRVWQPMTQVWGATWCFNGGPLRGPFSFRVTTLSTSETVVARNVIPRNWASNTCYRSRVNFR
