# Supplementary material for: Selaginella moellendorffii has a reduced and highly conserved expansin superfamily with genes more closely related to angiosperms than to bryophytes
Source: BMC Plant Biol. 2013 Jan 3;13:4. doi: 10.1186/1471-2229-13-4 (PMC3680112; doi:10.1186/1471-2229-13-4)
Supplement: Additional file 2 — Alignment for Figure 1. Alignment of Selaginella EXPA sequences with selected Arabidopsis, rice, and a Populus EXPA gene. [file 1471-2229-13-4-S2.pdf]

```
Majority     WTS-AHATFYGGSD--ASGTMG----------GACGYGNLYSQG-YGTNTAALSTALFNNGXSCGACYEIKCXXXP----WCLPG--SVVVTATNFCPPNXALPSD--NG
                      10        20        30        40        50        60        70        80        90       100       110

AtEXPA14.seq WVN-ARATFYGGAD--ASGTMG----------GACGYGNLYSQG-YGTNTAALSTALFNGGQSCGACFQIKCVDDP---KWCIGG--TITVTGTNFCPPNFAQANN--AG 89
AtEXPA15.seq WVN-AHATFYGGSD--ASGTMG----------GACGYGNLYSQG-YGTNTAALSTALFNNGLSCGACFEIKCQSDG---AWCLPG--AIIVTATNFCPPNNALPNN--AG 89
AtEXPA8.seq  WQG-GHATFYGGED--ASGTMG----------GACGYGNLYGQG-YGTNTAALSTALFNNGLTCGACYEMKCNDDP---RWCLGS--TITVTATNFCPPNPGLSND--NG 89
AtEXPA11.seq LTN-GHATFYGGSD--ASGTMG----------GACGYGDLYSAG-YGTMTAALSTALFNDGASCGECYRITCDHAA-DSRWCLKGA-SVVITATNFCPPNFALPNN--NG 92
AtEXPA12.seq WIR-AHATYYGVND--SPASLG----------GACGYDNPYHAG-FGAHTAALSGELFRSGESCGGCYQVRCDFPADP-KWCLRG-AAVTVTATNFCPTN-----N--NN 87
AtEXPA17.seq WLQ-AHATFYGGSD--ASGTMG----------GACGYGNLYTDG-YKTNTAALSTALFNDGKSCGGCYQILCDATK-VPQWCLKGK-SITITATNFCPPNFAQASD--NG 92
AtEXPA4.seq  WQN-AHATFYGGSD--ASGTMG----------GACGYGNLYSQG-YGTNTAALSTALFNNGMSCGACFELKCANDP---QWCHSGSPSILITATNFCPPNLAQPSD--NG 91
AtEXPA20.seq WKI-ATATLSRDRDGSSSVATG----------GACGYGDLRQSS-FAGYSAGLSGKLFNRGSSCGACLEVRCVNHIR---WCLQGSPSVVVTATDFCPPNSGLSSD--YG 93
AtEXPA13.seq WRP-ARATYYAATN--PRDAVG----------GACGYGDLVKSG-YGMATVGLSETLFERGQICGACFELRCVDDLR---WCIPGT-SIILTATNFCAPNYGFDPD--GG 90
AtEXPA22.seq WYD-ARATFYGDIH--GGDTQP------------CGYGNLFRQG-YGLATAALSTALFNDGYTCGACYEIMCTRDPQ---WCLPG--SVKITATNFCPANYSKTTD---- 85
AtEXPA7.seq  WRY-AHATFYGDET--GGETMG----------GACGYGNLFNSG-YGLSTAALSTTLFNDGYGCGQCFQITCSKSP----HCYSGK-STVVTATNLCPPNWYQDSN--AG 89
PtEXPA26.pro WKE-AHATFYEG----GSGTFG----------GACNYKDVAGQG-YGMNTAALSSVLFKNGQACGACFEIKCADNP---QWCKLGQPSLIVTATDHCPPNPSLPND--NG 150
OsEXPA4.seq  WQS-AHATFYGGGD--ASGTMG----------GACGYGNLYSQG-YGTNTAALSTALFNDGAACGSCYELRCDNAG--S-SCLPG--SITVTATNFCPPNYGLPSD--DG 89
OsEXPA5.seq  WSS-AHATFYGGGD--ASGTMG----------GACGYGNLYSQG-YGTNTAALSTALFNNGLSCGACFEVRCDAGGGGSHSCLPG--SVVVTATNFCPPNNALPSD--DG 92
OsEXPA7.seq  WQS-AHATFYGGSD--ASGTMG----------GACGYGNLYSQG-YGVNNAALSTALFNSGQSCGACFEIKCVNQPG-WEWCHPGSPSILITATNFCPPNYALPSD--NG 93
OsEXPA10.seq WRS-AKASYYAAD---PEDAIG----------GACGFGDLGKHG-YGMATVGLSTALFERGAACGGCYEVKCVDDLK---YCLPGT-SIVVTATNFCAPNFGLPAD--AG 89
OsEXPA11.seq WSS-GSATFYGGSD--ASGTMG----------GACGYGNLYSAG-YGTSTAALSTALFNNGQSCGACFEVRCGGGG----SCLAG--TVAVTATNLCPPNYALAGD--AG 88
OsEXPA12.seq -LS-GTATFYGGSD--ASGTMG----------GACGYGNLYSTG-YGTNTAALSSALFNDGAACGECYQITCDQS--NSKWCKAGT-SVTITATNLCPPDYSKPSN--DG 90
OsEXPA16.seq WRM-GSATYIKESLGHPLNDGG----------GACGYGDLDIFR-YGRYTAGVSGALFGRGSACGGCYEVRCVNHVL---WCLRGSPTVVVTATDFCAPNLGLSDD--YG 93
OsEXPA30.seq WTP-AHATFYGDET--ASETMG----------GACGYGNLYASG-YGTDTAALSTTLFKDGYGCGTCYQMRCVGTA----SCYRGSPAITVTATNLCPPNWAEDPDRGGG 92
OsEXPA32.seq WKQ-AHATFYGGRD--GSGTLD----------GACGYKDTSKEG-YGVQTVAVSTPLFGAGAGCGACYEVKCVDSP---DGCKVGAAPLVVTATNLCPPNPGQSND--NG 91
OsEXPA33.seq WTP-ATATFYGGSD--GAGTMG----------GACGYGNLYNAG-YGLNNAALSSALFNDGAMCGACYTIACDTS--QSTWCKPGT-SITITATNLCPPNYAKKSD--AG 91
AtEXPB3.seq  WLP-AVATWYGSPN--GDGSDG----------GACGYGTLVDVKPLHARVGAVNPILFKNGEGCGACYKVRCLDKS----ICSRR--AVTVIITDECP----------G 80
SmEXPA1      WTS-AHATFYGGSD--AAGTMG----------GACGYGNLYSQG-YGNNNAALSTALFNSGLSCGACFEIRCDSAA-DPRWCIAGT-SVVVTATNFCPPNYALANN--NG 92
SmEXPA2      WTG-AHATFYGGSD--ASGTMG----------GACGYGNLYSQG-YGTNTAALSTALFQSGLSCGACFEVKCNG---DPEWCLPGS-SVLVTATNFCPPNDALPNN--NG 90
SmEXPA3      WTD-AHATFYGGSD--ASGTMG----------GACGYGNLYSQG-YGTNTAALSTVLFNSGLSCGACFEIKCNAAK-DPQWCRAGA-SVTVTATNFCPPNYAQAND--NG 92
SmEXPA4      WTD-AHATFYGGSN--AAGTMG----------GACGYGNLVSAG-YGTNTAALSTALFQDGLSCGACFEVKCASGS-DPKWCLPG--SVVVTATNFCPPS-SQPSN--DG 90
SmEXPA5      WKQ-AFATFYGDET--ARETMG----------GACGYGNLYQSG-YGLMTAALSSTLFNSGYGCGQCYEITCTLSK----HCYFGK-SVVVTATNLCPPNWSKPSN--NG 89
SmEXPA6      WRY-AHATFYGEYD--ALETMGKSPDRTPPPRGACGYGNLYSQG-YGTDTTALSTVLFNSGYGCGGCYEISCTQSK----HCYPG--STIVTATNLCPPNWYKPSN--NG 98
SmEXPA7      WG--AHATYYGGSD--ASGTNN----------GACGYGNQLSAG-YGTITTALSTPLFRGGNVCGACYQVRC-WGD---PACLPGNPSVVVTATNLCPP--G---S--NG 84
SmEXPA8      WLD-AHATYYGGSD--ASGTNN----------GACGYGNQLSAG-YGYITTALSTPLFENGDICGACYEIRC-AG----TGCLPRNPSTVVTATNLCPP--G---S--NG 84
SmEXPA9      WQD-AHATFYGGSD--ASGTMG----------GACGYGNLYLQG-YGVSTAALSTALFNEGWSCGSCFELKCNAEA-DPEWCLPGNPSIVVTATNFCPPNFALPSD--NG 93
SmEXPA10     WTL-AHATYYGGSD--ASGTMG----------GACGYGNMYHEG-FGVETTALSTVLFQNGASCGACYELKCHQDP---KWCRPGNLSITVTATNFCPPNPARKSY--RG 91
SmEXPA11     WES-GHATFYGGSD--AAGTMG----------GACGYGNLYSQG-YGTNNAALSSALYNNGLSCGACFEVKCDAAA-DPQWCIPGR-SVTVTATNFCPP----------G 84
SmEXPA12     WSN-AHATFYGGSD--ASGTMG----------GACGYGNVLSAG-YGVNTAALSTALFNGGATCGACFQMQCVNS----RWCRPGK-SVTVTATNFCPPNNALPSD--NG 89
SmEXPA13     WTDGAHATYYGGSD--ASGTNN----------GACGYGNQLSAG-YGVLTTALSAPLFNDGHVCGACFEVKCSWGD---SGCLAGNPSIVVTATNLCPQ--G---S--NG 87
SmEXPA14     WLDGAHATYYGGSD--ASGTNN----------GACGYGNQLSAG-YGYITTALSTPLFENGDICGACFEIRC-AGG---AGCLPGNPSTVVTATNLCPP--G---S--NG 86
SmEXPA15     WG--AHATYYGGSD--ASGTNN----------GACGYGNQLSAG-YGTITTALSTPLFRGGNVCGACYQVRC-WGD---PACLPGNPSVVVTATNLCPP--G---S--NG 84
```

```
Majority       GWCNPPREHFDLSQPAFXRIAXYR-------AGIVPVQYRRVPCQRKG-GIRFTIN--GHSYFNLVLITNVGGAGDVXAVSXK-GSR-TGWQPMSRNWGQNWQS-NADLN

                         120       130       140       150       160       170       180       190       200       210       220

AtEXPA14.seq GWCNPPQHHFDLAQPIFLRIAQYK-------AGVVPVQYRRVACRRKG-GIRFTIN--GHSYFNLVLITNVAGAGDVISVSIK-GTN-TRWQSMSRNWGQNWQS-NAKLD 186
AtEXPA15.seq GWCNPPLHHFDLSQPVFQRIAQYK-------AGVVPVSYRRVPCMRRG-GIRFTIN--GHSYFNLVLVTNVGGAGDVHSVAVK-GSR-TRWQQMSRNWGQNWQS-NNLLN 186
AtEXPA8.seq  GWCNPPLQHFDLAEPAFLQIAQYR-------AGIVPVSFRRVPCMKKG-GIRFTIN--GHSYFNLVLISNVGGAGDVHAVSIK-GSKTQSWQAMSRNWGQNWQS-NSYMN 187
AtEXPA11.seq GWCNPPLKHFDMAQPAWEKIGIYR-------GGIVPVVFQRVSCYKKG-GVRFRIN--GRDYFELVNIQNVGGAGSIKSVSIK-GSKTG-WLAMSRNWGANWQS-NAYLD 189
AtEXPA12.seq GWCNLPRHHFDMSSPAFFRIARRGN------EGIVPVFYRRVGCKRRG-GVRFTMR--GQGNFNMVMISNVGGGGSVRSVAVR-GSKGKTWLQMTRNWGANWQS-SGDLR 186
AtEXPA17.seq GWCNPPRPHFDMAQPAFLTIAKYK-------AGIVPILYKKVGCRRSG-GMRFTIN--GRNYFELVLISNVAGGGEISKVWIK-GSKSNKWETMSRNWGANYQS-NTYLN 190
AtEXPA4.seq  GWCNPPREHFDLAMPVFLKIAQYR-------AGIVPVSYRRVPCRKRG-GIRFTIN--GHRYFNLVLITNVAGAGDIVRASVK-GSR-TGWMSLSRNWGQNWQS-NAVLV 188
AtEXPA20.seq GWCNFPKEHLELSHAAFTGIAETR-------AEMIPIQYRRVKCGRRG-GLRFSLS--GSSHFFQVLISNVGLDGEVVGVKVK-GHT-TAWIPMARNWGQNWHS-SLDLI 190
AtEXPA13.seq GHCNPPNKHFVLPIEAFEKIAIWK-------AGNMPVQYRRINCRKEG-SMRFTVD--GGGIFISVLITNVAGSGDIAAVKIK-GSR-TGWLPMGRNWGQNWHI-NADLR 187
AtEXPA22.seq LWCNPPQKHFDLSLAMFLKIAKYK-------AGVVPVRYRRIPCSKTG-GVKFETK--GNPYFLMVLIYNVGGAGDIKYVQVK-GNK-TGWITMKKNWGQNWTT-ITVLT 182
AtEXPA7.seq  GWCNPPRTHFDMAKPAFMKLAYWR-------AGIIPVAYRRVPCQRSG-GMRFQFQ--GNSYWLLIFVMNVGGAGDIKSMAVK-GSR-TNWISMSHNWGASYQA-FSSLY 186
PtEXPA26.pro GWCNVPREHFDVAKPVFSQLAEYE-------AGIIPIQYRRVPCQKQG-GIRFTIL--GNPWFYQVIVWNVGGAGDVVGVQVK-GDDKLKWTQMERDWGTTWKT-SAILL 248
OsEXPA4.seq  GWCNPPRPHFDMAEPAFLHIAQYR-------AGIVPVSFRRVPCVKKG-GVRFTVN--GHSYFNLVLVTNVAGAGDVRSVSIK-GSR-TGWQPMSRNWGQNWQS-NAFLD 186
OsEXPA5.seq  GWCNPPRAHFDMSQPVFQRIALFK-------AGIVPVSYRRVACQKKG-GIRFTIN--GHSYFNLVLVTNVGGAGDVHAVAVK-SERSAAWQALSRNWGQNWQS-AALLD 190
OsEXPA7.seq  GWCNPPRPHFDLAMPMFLHIAEYR-------AGIVPVSYRRVPCRKKG-GVRFTIN--GFRYFNLVLITNVAGAGDIVRASVK-GTS-TGWMPMSRNWGQNWQS-NSVLV 190
OsEXPA10.seq GVCNPPNHHFLLPIQSFEKIALWK-------AGVMPIQYRRVNCLRDG-GVRFAVA--GRSFFLTVLISNVGGAGDVRSVKIK-GTE-SGWLSMGRNWGQIWHI-NSDFR 186
OsEXPA11.seq GWCNPPRPHFDMAEPAFTRIAQAR-------AGVVPVQYRRVACAKQG-GIRFTIT--GHSYFNLVLVTNVGGAGDVTAVSVK-GSR-SGWQAMSHNWGANWQN-GANLD 185
OsEXPA12.seq GWCNPPRQHFDMAQPAWEQIGVYR-------GGIVPVNFQRVSCTRKG-GVRFTIN--GNSYFELVLITNVGGPGSIKSVQIK-GTKTG-WVTMSRNWGANWQA-NNYLN 187
OsEXPA16.seq GWCNFPKEHFEMSEAAFLRVAKAK-------ADIVPVQFRRVSCDRAG-GMRFTIT--GGASFLQVLITNVAADGEVAAVKVK-GSR-TGWIPMGRNWGQNWQC-DADLR 190
OsEXPA30.seq GWCNPPRAHFDLSKPAFMRMADWR-------AGIVPVMYRRVPCARAG-GLRFALQ--GNPYWLLAYVMNVAGAGDVGDMWVKAGGG-GGWVRMSHNWGASYQA-FAQLG 190
OsEXPA32.seq GWCNPPREHFDLSMPAFLQIAQEK-------AGIVPISYRRVPCVKVG-GIRYTIT--GNPYFNLVMVSNVGGAGDVAGLSVK-GNKRVKWTPLKRNWGQEWQT-SEVLT 189
OsEXPA33.seq GWCNPPRKHFDMSQPAWTSIAIYQ-------AGIVPVNFKRVPCQKSG-GIRFTIS--GRDYFELVTVFNVGGSGVVAQVSIK-GSKTD-WMAMSRNWGQNWQS-NAYLN 188
AtEXPB3.seq  --CSKTSTHFDLSGAVFGRLAIAGESGPLRNRGLIPVIYRRTACKYRGKNIAFHVNEGSTDFWLSLLVEFEDGEGDIGSMHIR-QAGAREWLEMKHVWGANWCIIGGPLK 187
SmEXPA1      GWCNPPLEHFDMAQPAWEQIGIYR-------GGIVPVQYRRVSCVKKG-GIHFTMN--GHTYFNLVLISNVGGAGDVHAVSIK-GSG-TGWQDMSRNWGQNWQS-NGQFQ 189
SmEXPA2      GWCNTPLQHFDMAQPAFEQIAKYR-------GGIVPVLYRRVPCQRKG-GIRFTMN--GHNYFNLVLVTNVGGAGDVHSVSIK-GSN-TDWLPMSRNWGQNWQS-NAILS 187
SmEXPA3      GWCNPPLEHFDMAQPAWEQIGIYR-------GGIVPVQYRRVSCVKKG-GIHFTLN--GNKYFMLVLVSNVGGAGDVRAVSIK-GPS-GDWQPMSRNWGQNWQS-DSRLI 189
SmEXPA4      GWCNSPLQHFDMAQPAFLKIAQYS-------AGIVPISYRRVSCSRSG-GIRFTMN--GHAYFNLVLITNVGGAGDVHAVSIK-GSG-TDWIPMSRNWGQNWQS-NALLG 187
SmEXPA5      GWCNPPRVHFDMSKPAFMKIAFWR-------AGIIPVSYRRVPCVRSG-GMNFKLG--GNRWWLMVFITNVGGSGDIKAVSVK-GSR-TGWIAMTRNWGVGFQV-FKQLQ 186
SmEXPA6      GWCNPPRIHFDMSKPAFSKIAYWR-------AGIVPVRYRRVPCRRKG-GIKFELK--GNRWWLIVFVSNVGGPGDIKRMAVK-GSK-TGWLPMSRNWGVGFQV-FKSLH 195
SmEXPA7      GWCDPPKPHFDLSQPAFSRIARIP-------NGHAQIQYRRVKCQRQG-GIRFTIN--GHTYFNLVLVTNVGGMGDVVGVSIK-GSS-SGWRSMSRNWGQNWEE-GSNLN 181
SmEXPA8      GWCDPPKQHFDLSQPAFSQIASIP-------YGHVLLQYRRVPCQRQG-AIHYTIN--GHTFFNLVLIENVGGSGDVVGVEIK-GSN-TNWMPMARNWGQNWMI-GGNLG 181
SmEXPA9      GWCNPPREHFDLSQPAFELIAKYR-------GGIVPVQYRRVPCEREG-GIHFSIN--GHAYFMLVLVWNVGGAGDVHAVAVM-GSRTRRWQPLVRNWGQNWQS-PDVLL 191
SmEXPA10     GWCNYPQQHFDLSMPAFVHLANRT-------AGIIPVIYTRVECKRQG-GIRFTMR--GNKWFILVMISNVGGAGDVRSVVVK-GSR--SWTPATRAWGQNWHISNRSML 188
SmEXPA11     SWCNEPLKHFDMSQPAWEEIGIYR-------GGIIPVYFRRVSCVRKG-GIHFTVN--GHAYFNLILITNVGGAGDVHAVSVK-GSG-TGWIPMSRNWGQNWQT-NAQLG 181
SmEXPA12     GWCNTPREHFDLSQPVWEQMAIYQ-------GGIVPVQYRRVKCYKQG-GIIFTMN--GNPNFNLVLIKNVAGWGDLRAVSIK-GSN-TGWLPMKRNWGSNWEY-HGVLV 186
SmEXPA13     GWCDSPKQHFDLAQPAFALIAVTL-------NGHVPIQYRRVSCKRDG-GLRFTIN--GHVYFNLVLIENVGGTGDVSAVSIK-GSK-TGWRPMTRNWGQNWQD-GGDLT 184
SmEXPA14     GWCDPPKPHFDLSQPAFSRIASIP-------NGHVQLQYRRVACDRQG-GIRFTVN--GHTFFNLVLVENVGGSGDVVAVEVK-GSA-TGWRQMQRNWGQNWQD-MGDLN 183
SmEXPA15     GWCDPPKPHFDLSQPAFSRIARIP-------NGHAQIQYRRVKCQRQG-GIRFTIN--GHTYFNLVLVTNVGGMGDVVGVSIK-GSS-SGWRSMSRNWGQNWEE-GSNLN 181
```

```
Majority     GQSLSFRVTT-SDGRTVTSYNVAPANWQFGQTFSG---KQF-

                   230       240       250       260

AtEXPA14.seq GQALSFKVTT-SDGRTVISNNATPRNWSFGQTYTG---KQF     223
AtEXPA15.seq GQALSFKVTA-SDGRTVVSNNIAPASWSFGQTFTG---RQF     223
AtEXPA8.seq  DQSLSFQVTT-SDGRTLVSNDVAPSNWQFGQTYQG---GQF     224
AtEXPA11.seq GQALSFSITT-TDGATRVFLNVVPSSWSFGQIYSSN--VQF     227
AtEXPA12.seq GQRLSFKVTL-TDSKTQTFLNVVPSSWWFGQTFSSRG-RQF     225
AtEXPA17.seq GQSLSFKVQL-SDGSIKAALNVVPSNWRFGQSFKSN--VNF     228
AtEXPA4.seq  GQALSFRVTG-SDRRTSTSWNMVPSNWQFGQTFVG---KNF     225
AtEXPA20.seq GQSLSFEVTL-KGGKTIASYDVAPPYWRFGMTYQG---KQF     227
AtEXPA13.seq NQALSFEVTS-SDRSTVTSYNVSPKNWNYGQTFEG---KQF     224
AtEXPA22.seq GQGLSFRVTT-SDGITKDFWNVMPKNWGFGQTFDG---RINF    220
AtEXPA7.seq  GQSLSFRVTSYTTGETIYAWNVAPANWSGGKTYKS---TANF    225
PtEXPA26.pro GESLSFRVSA-SDDRDSTSWHVTPKNWQFGQTYEG---KN      284
OsEXPA4.seq  GQSLSFQVTA-SDGRTVTSNNVAHPGWQFGQTFEG---GQF     223
OsEXPA5.seq  GQALSFRVTT-GDGRSVVSNNAVPRGWSFGQTFSG---AQF     227
OsEXPA7.seq  GQALSFRVTG-SDRRTSTSWNAAPAGWHFGQTFEG---KNF     227
OsEXPA10.seq GQPLSFELTS-SDGKTLTNYNVVPKEWDFGKTYTG---KQF     223
OsEXPA11.seq GQPLSFRVTA-SDGRTVTSDNVAPSGWSFGQTFSG---GQF     222
OsEXPA12.seq NQAISFSVTS-TAGKTLVFEDVAPSNWQFGQTFTSG--VQF     225
OsEXPA16.seq GQPLSFEVTG-GRGRTVVAYSVAPPDWMFAQTFEG---KQF     227
OsEXPA30.seq GQALSFKVTSYTTGQTILAAGVTPASWCFGLTYQA---RVNF    229
OsEXPA32.seq GESLTFRVMT-GDHRKATSWHVLPPDWQFGVTYQAT--KNF     227
OsEXPA33.seq TQSLSFKVKL-DDAREVTVWNIAPSNWNFGTTYTSN--INF     226
AtEXPB3.seq  G-PFSIKLTTLSAGKTLSATDVVPRNWAPKATYSSR--LNF     225
SmEXPA1      GQSLSFRVTT-SDGKSVVSMDVAPADWQYGQTFEG---SQFV    227
SmEXPA2      GQSLSFKVTT-SDGRTVVSYDAAPPNWQYGQTYSG---DQF     224
SmEXPA3      GQSLSFRVVT-SDNRAVTSLNVAPAGWSFGQTFSG---EQF     226
SmEXPA4      GQALSFKVTT-SDGKTTIAYNVAGANWAYGQTFEG---EQF     224
SmEXPA5      GQSLSFMVTCYSTGKTTVHNNVAPANWQLGSTYSA---KQL     224
SmEXPA6      GQSLSFMVTSFTTGKTVTAYDVVPANWRIGQAYSG---GQMV    234
SmEXPA7      GQALSFRVTT-SDGRTVTAYNVAPGDWQFGRTYTGNTASQYL    222
SmEXPA8      GQSLSFRVTG-SDGRKVTSLNVAPANWQFGRAYSG---GQFL    219
SmEXPA9      GQSLSFMVTT-SNGDTVTDYDVAPQDWKFGQTFVGN--KNL     229
SmEXPA10     EQGLSFVVST-SDGESRIALDVVPRNWKFGQTFTTG--AQFL    227
SmEXPA11     GQSLSFMVTD-SSGKTVISNNAAPSNWQYGQTFEG---EQFL    219
SmEXPA12     GQSLSFLLTP-SMGGSLISYDVFPRNWQFGQSYSG---RQFM    224
SmEXPA13     GQSLSFEVTT-SDGSKITAYDVAPDYWQFGQTFSG---GQFL    222
SmEXPA14     GQALSFRVTG-SDGKVVTSMNVAPADWQFGRTYSG---GQFL    221
SmEXPA15     GQALSFRVTT-SDGRTVTAYNVAPGDWQFV                210
```
