# Supplementary material for: Selaginella moellendorffii has a reduced and highly conserved expansin superfamily with genes more closely related to angiosperms than to bryophytes
Source: BMC Plant Biol. 2013 Jan 3;13:4. doi: 10.1186/1471-2229-13-4 (PMC3680112; doi:10.1186/1471-2229-13-4)
Supplement: Additional file 3 — Alignment for Figure 2. Alignment of Selaginella and Physcomitrella EXPA sequences with selected Arabidopsis, rice and a Populus EXPA gene. [file 1471-2229-13-4-S3.pdf]

```
Majority      WTD-AHATFYGGSD--ASGTM----G----------G-ACGYGNLYSQGYGTNTAALSTALFNGGLSCGACYELKCDP--GS-------KWCLPGS-SITVTATNFCPPN----SD---G
                        10        20        30        40        50        60        70        80        90       100       110       120

AtEXPA12.seq WIR-AHATYYGVND--SPASL----G----------G-ACGYDNPYHAGFGAHTAALSGELFRSGESCGGCYQVRCDFPADP-------KWCLRG-AAVTVTATNFCPTN-----N--NN 87
AtEXPA17.seq WLQ-AHATFYGGSD--ASGTM----G----------G-ACGYGNLYTDGYKTNTAALSTALFNDGKSCGGCYQILCDATKVP-------QWCLKG-KSITITATNFCPPNFAQASD--NG 92
AtEXPA11.seq LTN-GHATFYGGSD--ASGTM----G----------G-ACGYGDLYSAGYGTMTAALSTALFNDGASCGECYRITCDHAADS-------RWCLKG-ASVVITATNFCPPNFALPNN--NG 92
AtEXPA22.seq WYD-ARATFYGDIHG----GD----T----------Q-PCGYGNLFRQGYGLATAALSTALFNDGYTCGACYEIMCTR--DP-------QWCLPGS--VKITATNFCPANYSKTTD--L- 86
AtEXPA14.seq WVN-ARATFYGGAD--ASGTM----G----------G-ACGYGNLYSQGYGTNTAALSTALFNGGQSCGACFQIKCVD--DP-------KWCIGG--TITVTGTNFCPPNFAQANN--AG 89
AtEXPA15.seq WVN-AHATFYGGSD--ASGTM----G----------G-ACGYGNLYSQGYGTNTAALSTALFNNGLSCGACFEIKCQS--DG-------AWCLPG--AIIVTATNFCPPNNALPNN--AG 89
AtEXPA8.seq  WQG-GHATFYGGED--ASGTM----G----------G-ACGYGNLYGQGYGTNTAALSTALFNNGLTCGACYEMKCND--DP-------RWCLGS--TITVTATNFCPPNPGLSND--NG 89
AtEXPA22.seq WYD-ARATFYGDIHG----GD----T----------Q-PCGYGNLFRQGYGLATAALSTALFNDGYTCGACYEIMCTR--DP-------QWCLPGS--VKITATNFCPANYSKTTD--L- 86
AtEXPA4.seq  WQN-AHATFYGGSD--ASGTM----G----------G-ACGYGNLYSQGYGTNTAALSTALFNNGMSCGACFELKCAN--DP-------QWCHSGSPSILITATNFCPPNLAQPSD--NG 91
AtEXPA13.seq WRP-ARATYYAATN--PRDAV----G----------G-ACGYGDLVKSGYGMATVGLSETLFERGQICGACFELRCVD--DL-------RWCIPGT-SIILTATNFCAPNYGFDPD--GG 90
AtEXPA20.seq WKI-ATATLSRDRDGSSSVAT----G----------G-ACGYGDLRQSSFAGYSAGLSGKLFNRGSSCGACLEVRCVN--HI-------RWCLQGSPSVVVTATDFCPPNSGLSSD--YG 93
AtEXPA7.seq  WRY-AHATFYGDET--GGETM----G----------G-ACGYGNLFNSGYGLSTAALSTTLFNDGYGCGQCFQITCSK--S--------PHCYSGK-STVVTATNLCPPNWYQDSN--AG 89
OsEXPA12.seq -LS-GTATFYGGSD--ASGTM----G----------G-ACGYGNLYSTGYGTNTAALSSALFNDGAACGECYQITCDQS-NS-------KWCKAG-TSVTITATNLCPPDYSKPSN--DG 90
OsEXPA33.seq WTP-ATATFYGGSD--GAGTM----G----------G-ACGYGNLYNAGYGLNNAALSSALFNDGAMCGACYTIACDTS-QS-------TWCKPG-TSITITATNLCPPNYAKKSD--AG 91
OsEXPA4.seq  WQS-AHATFYGGGD--ASGTM----G----------G-ACGYGNLYSQGYGTNTAALSTALFNDGAACGSCYELRCDN--AG------S-SCLPG--SITVTATNFCPPNYGLPSD--DG 89
OsEXPA5.seq  WSS-AHATFYGGGD--ASGTM----G----------G-ACGYGNLYSQGYGTNTAALSTALFNNGLSCGACFEVRCDA--GGGG----SHSCLPG--SVVVTATNFCPPNNALPSD--DG 92
OsEXPA11.seq WSS-GSATFYGGSD--ASGTM----G----------G-ACGYGNLYSAGYGTSTAALSTALFNNGQSCGACFEVRCGG--GG--------SCLAG--TVAVTATNLCPPNYALAGD--AG 88
OsEXPA7.seq  WQS-AHATFYGGSD--ASGTM----G----------G-ACGYGNLYSQGYGVNNAALSTALFNSGQSCGACFEIKCVN--QPG-----WEWCHPGSPSILITATNFCPPNYALPSD--NG 93
OsEXPA32.seq WKQ-AHATFYGGRD--GSGTL----D----------G-ACGYKDTSKEGYGVQTVAVSTPLFGAGAGCGACYEVKCVD--SP-------DGCKVGAAPLVVTATNLCPPNPGQSND--NG 91
OsEXPA16.seq WRM-GSATYIKESLGHPLNDG----G----------G-ACGYGDLDIFRYGRYTAGVSGALFGRGSACGGCYEVRCVN--HV-------LWCLRGSPTVVVTATDFCAPNLGLSDD--YG 93
OsEXPA10.seq WRS-AKASYYAAD---PEDAI----G----------G-ACGFGDLGKHGYGMATVGLSTALFERGAACGGCYEVKCVD--DL-------KYCLPGT-SIVVTATNFCAPNFGLPAD--AG 89
OsEXPA30.seq WTP-AHATFYGDET--ASETM----G----------G-ACGYGNLYASGYGTDTAALSTTLFKDGYGCGTCYQMRCVG--T--------ASCYRGSPAITVTATNLCPPNWAEDPDRGGG 92
PpEXPA9.seq  WTD-AHATFYGGAD--ASGTQGGCAG----------G-ACGFGNLYSTGYGTNTAALSQALFNSGLTCGACFELVCDSS-GS-------RYCVTSS-SVVVTATNFCPTG----ST---G 90
PpEXPA8.seq  WNE-AHATFYGGSD--AGGTTGTR-G----------G-ACGYGDLYSTGYGTSTVAISSALFDRGLACGACYQVKCAG--SS-------SECRSDSPAIQVTVTNFCPPNPSLPED--NG 94
PpEXPA7.seq  WGY-AHATYYXGAD--ASGTQ----G----------GGACGFGNLYSTGYGTNTAALSAALFNSGLSCGSCYELACDPN-GS-------KYCLPGGPTVTVTATNFCPHG----SL---G 88
PpEXPA6.seq  WRN-ARSTFYGGMD--AAGTM----S----------G-ACGYGNLYASGYGVHTTALSSALFKNGMACGACFEVQCGG--KG-------KPCKPGS--VVVTATNFCPPNPGQSAN--NG 89
PpEXPA5.seq  WGR-AHATFYGGAD--ASGTQ----G----------G-ACGYGNLYSTGYGTNTAALSSSLFNSGLSCGACYELTCDPS-CS-------QYCLPGG-SAIITATNFCPTG----SN---G 86
PpEXPA4.seq  WKD-AHITYYGSPN--GGGTQ----G----------G-ACGYQNTYALGYGSFTAALSAPLFQGGAACGGCYQLKCAPVRETRT---VHNWCWSYSRSIVVTATNLCPPG----SH---G 92
PpEXPA3.seq  SGV-AHATFYGGVD--AQGTQ----G----------G-ACGYGNLYSTGYGTSTTALSSALFNAGLSCGACFELKCDSA-NS-------KYCLPGDKSITVTATNYCPQG----SD---G 87
PpEXPA27.seq WAT-ATATFYGGAD--AG-------G----------G-ACGYGNLYSTGYGASTTALSAPLXNGGSACGACYQLQCAR---S-------NHCYAGR-SITVTATNFCPTG----SE---G 81
PpEXPA26.seq WNF-GRITYYGSPN--GGGTQ----G----------G-ACGYQNTFALGYGTNTAALSSRLFQGGAACGACYQLRCIAPKWG------KNWCWNYARSIVVTATNLCPSG----SN---G 89
PpEXPA25.seq WRD-AHITYYGSPN--GGGTQ----G----------G-ACAYQNTFSLGYGAMTAALSSPLFEGGAACGACYQLQCKRVQETRT---VKNWCWSYSRTITITATNLCPPG----SA---G 92
PpEXPA24.seq WSS-GRITYYGSPN--GGGTQ----G----------G-ACGYQNTVSLGYGFMTAALSTPLFNGGAACGACYQLQCAPVHETPKNLLQRNWCWKVGRRITITATNLCPPG----SE---G 95
PpEXPA23.seq WIN-GRITYYGSPN--GGGTQ----G----------G-ACGYQNTVSLGYGFMTAALSTTLFKGGAACGACYQLQCAPVSETPSGLLKRNWCWQVGRSILVTATNLCPPG----SS---G 95
PpEXPA22.seq WQD-AHITYYGSPN--GGGTQ----G----------G-ACGYQNTVSLGYGFMTAALSSPLFQGGKACGACFQLQCARVQETRT---VKNWCHDYSKAITVTATNLCPPG----SE---G 92
PpEXPA21.seq .RD-AHITYYGSPN--GGGTE----G----------G-ACGYQNTYSLGYGFMTAALSSSLFQGGSSCGACYQIRCEPIRVTRT---VKNWCWSYSRTITVTAINLCPPR----SS---G 92
PpEXPA20.seq WKD-AHITYYGSPN--GGGTQ----G----------G-A--YQNTYALGY.PLTAALSSPLFQGGAACESCYQLKCALVRAS.I---ARNWCWNYFRTIVVTATNLCPRG----SH---G 90
PpEXPA2.seq  WKE-AHITYYGTAN--GGGTQ----G----------G-ACGYPNTFAMGYGVMTAALSYPLFQGGKSCGACYQLKCKWLAPTRT---VHNWCWSYSRTITITATNSCPPG----SH---G 92
PpEXPA19.seq WGD-AHITYYGSPN--GAGTE----G----------G-ACGYQNTYKLGYGSMTAALSSRLFQGGKACGGCYQLRCAPNRG-------RNWCWSYARAIVVTATNLCPQG----SH---G 88
PpEXPA18.seq WNE-AHITYYGTAN--GGGTQ----G----------G-ACGYPNTFAMGYGAMTAALSYPLFQGGKACGGCYQLRCKWVTPTRT---VHNWCWSYSRTITVTATNSCPPG----SH---G 92
PpEXPA17.seq WGR-AHATYYGGAD--ASGTQ----G----------G-ACGFGNLYSSGYGTSTAALSSSLFNSGLSCGACYELTCDPS-GS-------QYCLPGG-SAIITVTNFCPTG----SN---G 86
```

```
PpEXPA16.seq WDT-AHATYYGGRD--AGGTM----G----------G-ACGYGNLYNTGYGVKTAALSAPLFKGGATCGACYELTCILS-QS-------KYCYQNK-KILITATNFCPTG----ST---G 86
PpEXPA15.seq WGY-AHATYYGGAD--ASGTQ----G----------GGACGFGNLYSTGYGTNTAALSAALFNSGLSCGSCYELACDPN-GS-------KYCLPGGRTVTVTATNFCPHG----SL---G 88
PpEXPA14.seq WDK-GHATYYGEGD--ARGTM----G----------G-ACGYSNLYSTGYGVNTAALSGPLFNGGATCGACYELTCILN-ES-------KWCYRGK-NIIVTATNFCPSG----ST---G 86
PpEXPA13.seq WNE-ARATFYGGSD--AGGTT----G----------G-ACGYGDLYSTGYGTNTVATSSAIFDRGLACGACYQVKCAG--SA-------SECQPGTPAIQVTVTNFCPPNPSLPEG--NG 91
PpEXPA12.seq WND-AHATYYGGAD--AS-------G----------G-ACGFGNLYSTGYGTSTAALSQALFNSGLTCGACFELACDPS-GS-------KYCYKGS-SIVVTATNFCPSG----SE---G 83
PpEXPA11.seq WGR-AHATYYGGAD--ASGTQ----G----------G-ACGFGNLYSSGYGTDTAALSSALFNSGLSCGACYELTCDPS-GS-------KFCIPGG-SAIITVTNFCPTG----SN---G 86
PpEXPA10.seq SGR-AHATFYGGAD--ASGTQ----G----------G-ACGYGNLYSTGYGTSTAALSSALFNSGLSCGACYELTCDTS-GS-------KYCLPGNPSIILTATNYCPQN----SN---G 87
PpEXPA1.seq  WRK-AHATFYGGAD--ASGTM----G----------G-ACGYGNLYSTGYGVDSTALSTALFNNGAKCGACFAIQCY---RS-------QYCVPGSPVITVTATNFCPPN-HKGDG--TP 89
PtEXPA26.pro WKE-AHATFYEG----GSGTF----G----------G-ACNYKDVAGQGYGMNTAALSSVLFKNGQACGACFEIKCAD--NP-------QWCKLGQPSLIVTATDHCPPNPSLPND--NG 150
SmEXPA1      WTS-AHATFYGGSD--AAGTM----G----------G-ACGYGNLYSQGYGNNNAALSTALFNSGLSCGACFEIRCDS--AA-D----PRWCIAGT-SVVVTATNFCPPNYALANN--NG 92
SmEXPA2      WTG-AHATFYGGSD--ASGTM----G----------G-ACGYGNLYSQGYGTNTAALSTALFQSGLSCGACFEVKCNG-----D----PEWCLPGS-SVLVTATNFCPPNDALPNN--NG 90
SmEXPA3      WTD-AHATFYGGSD--ASGTM----G----------G-ACGYGNLYSQGYGTNTAALSTVLFNSGLSCGACFEIKCNA--AK-D----PQWCRAGA-SVTVTATNFCPPNYAQAND--NG 92
SmEXPA4      WTD-AHATFYGGSN--AAGTM----G----------G-ACGYGNLVSAGYGTNTAALSTALFQDGLSCGACFEVKCAS--GS-D----PKWCLPG--SVVVTATNFCPPS-SQPSN--DG 90
SmEXPA5      WKQ-AFATFYGDET--ARETM----G----------G-ACGYGNLYQSGYGLMTAALSSTLFNSGYGCGQCYEITCTL--S--------KHCYFGK-SVVVTATNLCPPNWSKPSN--NG 89
SmEXPA6      WRY-AHATFYGEYD--ALETM----GKSPDRTPPPRG-ACGYGNLYSQGYGTDTTALSTVLFNSGYGCGGCYEISCTQ--S--------KHCYPG--STIVTATNLCPPNWYKPSN--NG 98
SmEXPA7      WG--AHATYYGGSD--ASGTN----N----------G-ACGYGNQLSAGYGTITTALSTPLFRGGNVCGACYQVRC-W--GD-------PACLPGNPSVVVTATNLCPP-----GS--NG 84
SmEXPA8      WLD-AHATYYGGSD--ASGTN----N----------G-ACGYGNQLSAGYGYITTALSTPLFENGDICGACYEIRC-A--G--------TGCLPRNPSTVVTATNLCPP-----GS--NG 84
SmEXPA9      WQD-AHATFYGGSD--ASGTM----G----------G-ACGYGNLYLQGYGVSTAALSTALFNEGWSCGSCFELKCNA--EA-D----PEWCLPGNPSIVVTATNFCPPNFALPSD--NG 93
SmEXPA10     WTL-AHATYYGGSD--ASGTM----G----------G-ACGYGNMYHEGFGVETTALSTVLFQNGASCGACYELKCHQ--DP-------KWCRPGNLSITVTATNFCPPNPARKSY--RG 91
SmEXPA11     WES-GHATFYGGSD--AAGTM----G----------G-ACGYGNLYSQGYGTNNAALSSALYNNGLSCGACFEVKCDA--AA-D----PQWCIPGR-SVTVTATNFCPP---------G 84
SmEXPA12     WSN-AHATFYGGSD--ASGTM----G----------G-ACGYGNVLSAGYGVNTAALSTALFNGGATCGACFQMQCVN--S--------RWCRPGK-SVTVTATNFCPPNNALPSD--NG 89
SmEXPA13     WTDGAHATYYGGSD--ASGTN----N----------G-ACGYGNQLSAGYGVLTTALSAPLFNDGHVCGACFEVKCSW--GD-------SGCLAGNPSIVVTATNLCPQ-----GS--NG 87
SmEXPA14     WLDGAHATYYGGSD--ASGTN----N----------G-ACGYGNQLSAGYGYITTALSTPLFENGDICGACFEIRC-A--GG-------AGCLPGNPSTVVTATNLCPP-----GS--NG 86

Majority     GWCNPPRQHFDLSQPAFLKIARY-RGGVVPVQYRRVPCVKKGGIRFTINGNPYFLLVLITNVGGAGDVQAVSIK-GSK-TG-WXPMSRNWGQNWQSNA---XLXGQALSFRVTT-SDGRT
                       130       140       150       160       170       180       190       200       210       220       230       240

AtEXPA12.seq GWCNLPRHHFDMSSPAFFRIARRGNEGIVPVFYRRVGCKRRGGVRFTMRGQGNFNMVMISNVGGGGSVRSVAVR-GSK-GKTWLQMTRNWGANWQSSG---DLRGQRLSFKVTL-TDSKT 201
AtEXPA17.seq GWCNPPRPHFDMAQPAFLTIAKY-KAGIVPILYKKVGCRRSGGMRFTINGRNYFELVLISNVAGGGEISKVWIK-GSK-SNKWETMSRNWGANYQSNT---YLNGQSLSFKVQL-SDGSI 205
AtEXPA11.seq GWCNPPLKHFDMAQPAWEKIGIY-RGGIVPVVFQRVSCYKKGGVRFRINGRDYFELVNIQNVGGAGSIKSVSIK-GSK-TG-WLAMSRNWGANWQSNA---YLDGQALSFSITT-TDGAT 204
AtEXPA22.seq -WCNPPQKHFDLSLAMFLKIAKY-KAGVVPVRYRRIPCSKTGGVKFETKGNPYFLMVLIYNVGGAGDIKYVQVK-GNK-TG-WITMKKNWGQNWTTIT---VLTGQGLSFRVTT-SDGIT 197
AtEXPA14.seq GWCNPPQHHFDLAQPIFLRIAQY-KAGVVPVQYRRVACRRKGGIRFTINGHSYFNLVLITNVAGAGDVISVSIK-GTNTR--WQSMSRNWGQNWQSNA---KLDGQALSFKVTT-SDGRT 201
AtEXPA15.seq GWCNPPLHHFDLSQPVFQRIAQY-KAGVVPVSYRRVPCMRRGGIRFTINGHSYFNLVLVTNVGGAGDVHSVAVK-GSRTR--WQQMSRNWGQNWQSNN---LLNGQALSFKVTA-SDGRT 201
AtEXPA8.seq  GWCNPPLQHFDLAEPAFLQIAQY-RAGIVPVSFRRVPCMKKGGIRFTINGHSYFNLVLISNVGGAGDVHAVSIK-GSKTQS-WQAMSRNWGQNWQSNS---YMNDQSLSFQVTT-SDGRT 202
AtEXPA22.seq -WCNPPQKHFDLSLAMFLKIAKY-KAGVVPVRYRRIPCSKTGGVKFETKGNPYFLMVLIYNVGGAGDIKYVQVK-GNK-TG-WITMKKNWGQNWTTIT---VLTGQGLSFRVTT-SDGIT 197
AtEXPA4.seq  GWCNPPREHFDLAMPVFLKIAQY-RAGIVPVSYRRVPCRKRGGIRFTINGHRYFNLVLITNVAGAGDIVRASVK-GSR-TG-WMSLSRNWGQNWQSNA---VLVGQALSFRVTG-SDRRT 203
AtEXPA13.seq GHCNPPNKHFVLPIEAFEKIAIW-KAGNMPVQYRRINCRKEGSMRFTVDGGGIFISVLITNVAGSGDIAAVKIK-GSR-TG-WLPMGRNWGQNWHINA---DLRNQALSFEVTS-SDRST 202
AtEXPA20.seq GWCNFPKEHLELSHAAFTGIAET-RAEMIPIQYRRVKCGRRGGLRFSLSGSSHFFQVLISNVGLDGEVVGVKVK-GHT-TA-WIPMARNWGQNWHSSL---DLIGQSLSFEVTL-KGGKT 205
AtEXPA7.seq  GWCNPPRTHFDMAKPAFMKLAYW-RAGIIPVAYRRVPCQRSGGMRFQFQGNSYWLLIFVMNVGGAGDIKSMAVK-GSR-TN-WISMSHNWGASYQAFS---SLYGQSLSFRVTSYTTGET 202
OsEXPA12.seq GWCNPPRQHFDMAQPAWEQIGVY-RGGIVPVNFQRVSCTRKGGVRFTINGNSYFELVLITNVGGPGSIKSVQIK-GTK-TG-WVTMSRNWGANWQANN---YLNNQAISFSVTS-TAGKT 202
OsEXPA33.seq GWCNPPRKHFDMSQPAWTSIAIY-QAGIVPVNFKRVPCQKSGGIRFTISGRDYFELVTVFNVGGSGVVAQVSIK-GSK-TD-WMAMSRNWGQNWQSNA---YLNTQSLSFKVKL-DDARE 203
OsEXPA4.seq  GWCNPPRPHFDMAEPAFLHIAQY-RAGIVPVSFRRVPCVKKGGVRFTVNGHSYFNLVLVTNVAGAGDVRSVSIK-GSR-TG-WQPMSRNWGQNWQSNA---FLDGQSLSFQVTA-SDGRT 201
OsEXPA5.seq  GWCNPPRAHFDMSQPVFQRIALF-KAGIVPVSYRRVACQKKGGIRFTINGHSYFNLVLVTNVGGAGDVHAVAVK-SERSAA-WQALSRNWGQNWQSAA---LLDGQALSFRVTT-GDGRS 205
OsEXPA11.seq GWCNPPRPHFDMAEPAFTRIAQA-RAGVVPVQYRRVACAKQGGIRFTITGHSYFNLVLVTNVGGAGDVTAVSVK-GSR-SG-WQAMSHNWGANWQNGA---NLDGQPLSFRVTA-SDGRT 200
```

```
OsEXPA7.seq  GWCNPPRPHFDLAMPMFLHIAEY-RAGIVPVSYRRVPCRKKGGVRFTINGFRYFNLVLITNVAGAGDIVRASVK-GTS-TG-WMPMSRNWGQNWQSNS---VLVGQALSFRVTG-SDRRT 205
OsEXPA32.seq GWCNPPREHFDLSMPAFLQIAQE-KAGIVPISYRRVPCVKVGGIRYTITGNPYFNLVMVSNVGGAGDVAGLSVK-GNKRVK-WTPLKRNWGQEWQTSE---VLTGESLTFRVMT-GDHRK 204
OsEXPA16.seq GWCNFPKEHFEMSEAAFLRVAKA-KADIVPVQFRRVSCDRAGGMRFTITGGASFLQVLITNVAADGEVAAVKVK-GSR-TG-WIPMGRNWGQNWQCDA---DLRGQPLSFEVTG-GRGRT 205
OsEXPA10.seq GVCNPPNHHFLLPIQSFEKIALW-KAGVMPIQYRRVNCLRDGGVRFAVAGRSFFLTVLISNVGGAGDVRSVKIK-GTE-SG-WLSMGRNWGQIWHINS---DFRGQPLSFELTS-SDGKT 201
OsEXPA30.seq GWCNPPRAHFDLSKPAFMRMADW-RAGIVPVMYRRVPCARAGGLRFALQGNPYWLLAYVMNVAGAGDVGDMWVKAGGG-GG-WVRMSHNWGASYQAFA---QLGGQALSFKVTSYTTGQT 206
PpEXPA9.seq  GWCDYPRQHFDLSQPVFTRIAQP-VGGVVTLKYRRVRCQKSGGIRFTITGNPYFILVLVTNVGGAGDVQQLYIK-GSS-TG-WNAMSRNWGQLWEIR-NAA-LMGQALSFRVVT-SDRAE 203
PpEXPA8.seq  GWCNLPLHHFDMSMPAFEQIATY-KAGIVPVMYRRTSCVRTGGIHFTMSGHNFMNLVLVTNVGGMGDVQSVSIR-GS-KTS-WVTMTRNFGQIWQSTV---NMSGQSLSFMVTT-SDGKT 206
PpEXPA7.seq  GWCDAPKQHFDLAHPMFVSLARE-VGGVIPIKYRRVPCVKSGGMRFTINGNPWFLLVLVTNVAGAGDVQHMYIK-GSN-TP-WEPMSRNWGSMWQFTGDSK-MKGQALSFKAVT-SDGSV 202
PpEXPA6.seq  GWCNPPNEHFDLSYPAFVKIADP-KAGAVPLQYRRVPCQKQGGIRFTINGNCNFILVTITNVGGSGVVTAAYLK-GDK-TE-WSPLSRNWGANWQCRR---NYCGQGISIKIVT-SDNKV 201
PpEXPA5.seq  GWCNPPKQHFDLAQPVFSKIART-VGGVIPINYRRVPCSKSGGMRFTVNGNPYFLLVLVTNVGGAGDVQQLYIK-GAS-TG-WLPLKRNWGQMWQFTGNSG-MHGQAISFKAVT-SDGAE 200
PpEXPA4.seq  GWCAW-RPHFDLPMPAFTSLAKQ-VGGVAPVFYRRVRCAKRGGVRFTIGGNPYFLMVLIHNVGGAGDIRSVRIK-GQY-SG-WVTMFRNWGSLWTCRT---KLSG-PLSFMITT-SDGRT 202
PpEXPA3.seq  GWCDSPKQHFDLSHPMFTSLAQE-VGGVIPVTYRRAPCAKKGGMRFTINGNPWFVMILVTNCGGAGDVQQLQIR-GSD-TP-WYPCVRNWGQMWQMTSDPN-LPGKALSFRATL-SDGSV 201
PpEXPA27.seq GWCNPPRKHFDLSMPMFTTLARQ-VAGVVPVDYRRVACNKKGGQRFLMTGNPYFIMVLVYNVAGAGDVQRFFVK-GSM-TG-WYELRRNWGQIWTCTADSR-LKGQALSFRTQT-SDGRQ 195
PpEXPA26.seq GWCPS-QAHFDXPMPAFTSLARK-EGGVTPIMYRRVRCARRGGIRFTIGGNPFFLMVLIHNVGGAGDVRAVSIK-GQY-TG-WVGMYRNWGSLWTCTT---KIDG-ALTFRITT-GDGKT 199
PpEXPA25.seq AWCDPPRHHFDLTMPAFLTLARR-EGGVAPVLYRRVKCVKRGGIRFTIGGNPWFLMILIHNVAGAGDVRAVRIK-TPS-TD-WIPMYRNWGALWTVQR---KLSG-PLSFQITA-GDRRQ 203
PpEXPA24.seq GWCDPPQHHFDLPMPAFTALAKR-EGGVVPIYYRRVRCAKQGGIRFTMGGNPWFLMILIHNVAGAGDVVAVKIK-CPT-SD-WCDMYRNWGAFWTVQK---KMEG-PLSFQITT-XDRRK 206
PpEXPA23.seq GWCNPPQHHFDLPMPAFLALARR-EGGVVPVYYRKVNCARKGGIRFTVGGNPWFLMILIHNVGGAGDVVAVKVK-CPT-SG-WYDMYRNWGALWTVQK---KMNG-PLSFAITT-SDGRT 206
PpEXPA22.seq TWCDPPRHHFDLPMPAFLSLARQ-EGGVAPVYYRRVQCLKKGGIRFTMGGNPWFLMVLVHNVXGAGDVVXVKVK-CPS-SG-WYDMYRNWGALWTVQK---KMVG-PLSFLLTT-GDGRR 203
PpEXPA21.seq GCCNPPLQHFDLPMPAFLSLARR-EGGVAPV.YTKVRSEKRGGIRFTMGGNLWFLTILIHNVGGAGDVRSVRIK-SPH-SG-LISMYRNWGSLWTVRA---RMSG-ALFFMITT-SHGRV 203
PpEXPA20.seq GWCDY-RPHFDLPMPAFTALARR-EGGVAPVFYRRVKCVKRGGVRFTIGGNPYFTMILIDNVGGAGDIRSMRVK-GQY-GG-WVNIFRNWGSIRTCRT---KVAG-ALSFMITT-TDGRS 200
PpEXPA2.seq  GWCD-WKPHFDLPMPAFMTLARR-EGGVAPVYYRKVRCAKRGGIRFTLGGNPYFMMILIHNVGGAGDLRAVKIK-GRN-GY-WVPMWQNWGALWTCKT---KLSG-ALSFQITT-GDGRT 202
PpEXPA19.seq GWCDYPKSHFDLPMPAFTSLARR-EGGVAPVWYRKVRCAKRGGVRFTIGGNPWFLMVLIHNVGGAGDVVSVKVK-CPY-TG-WVSAYRNWGCLWTVRT---KMTG-PLSFTLVT-SDGRT 199
PpEXPA18.seq GWCN-WRPHFDLPMPAFLTLARR-EGGVAPVYYRKVRCAKRGGIRFTIGGNPYFLMILIHNVGGAGDLKAVKVR-GGN-GY-WVPMWRNWGALWTCKT---RMSG-ALSFQITT-GDGRT 202
PpEXPA17.seq GWCNPPRQHFDLAQPVFSKIART-VGGVIPINYRRVSCLKSGGMRFTVNGNPYFLLVLVTNVGGAGDVQQLYIK-GSS-TA-WLPLKRNWGQMWQFTGNSG-MHGQAISFKAVT-SDGAE 200
PpEXPA16.seq GWCNPPRKHFDLSEPMFTTLANR-VGGVIPVNFRRVHCYKKGGMRFTINGNPYHMMVLVYNVAGAGDVQQMFIK-SPT-TG-WLQMSRNWGQIWTYKGGPRNIVGFALSFRVYT-SDRRQ 201
PpEXPA15.seq GWCDSPKQHFDLAHPMFVTLAKE-VGGVIPIKFRRVPCVKSGGMHFTINGNPWFLLVLVTNVAGAGDLQQVYIK-GSN-TP-WEPMSRNWGSMWQFTGNSK-MKGQALSFKTIT-SDGAV 202
PpEXPA14.seq GWCNPPQKHFDLSEPMFTTLANR-VGGVIPVNFRRVACYKQGGMRFTINGNPYFFIVLVYNVAGAGDVQQVYIK-GPK-TQ-WLQMYRNWGSQWTFNGGPNNIVGSALSFRVHT-SDGRQ 201
PpEXPA13.seq GWCNLPLHHFDMAMPAFQQIASY-RVGIVPILYRRASCVRTGGIRFTMSGHKFMNLVLVTNVGGMGDVQTVFIQ-GS-KTK-LVAMIRNFGQIWQSSV---NVSGQRLSFMVMT-SDGES 203
PpEXPA12.seq GWCDSPKQHFDLSQPVFNKIAQQ-AGGVIPVKYRRVPCRKSGGMRFTINGNPYFILVLVTNVGGAGDVQQLSLK-GSS-TG-WYTMSRNWGQQWEFRGNSA-LVGQALSFRVVT-SDGAE 197
PpEXPA11.seq GWCNPPKQHFDLAQPVFRKIART-VGGVVPINYRRVSCLKDGGMRFTVNGNPYFLLVLVTNVGGAGDVQQLYMK-GSS-TN-WQPLKRNWGQMWQFTGNSR-MHGQAISFKAVT-SDGSV 200
PpEXPA10.seq GWCDAPKQHFDLAHPMFVSLAEE-RGGVIPVNYRRVPCAKKGGMRFQMNGNPWFLLVLVTNVGGAGDVQQLSIK-GSN-SG-WYQMKRNWGQMWQLTGNSN-MPGQALSFRAVL-SDGTT 201
PpEXPA1.seq  GWCNPPMRHFDLAQPSFTKIAKY-RAGIVPVLFRRVPCEKKGGVRFTINGNKYFNLVLVHNVGGKGDVHAVDIK-GSN-TE-WIPMKRNWGMNWQTDA---VMTGQALSFRVTT-SDGKT 201
PtEXPA26.pro GWCNVPREHFDVAKPVFSQLAEY-EAGIIPIQYRRVPCQKQGGIRFTILGNPWFYQVIVWNVGGAGDVVGVQVK-GDDKLK-WTQMERDWGTTWKTSA---ILLGESLSFRVSA-SDDRD 263
SmEXPA1      GWCNPPLEHFDMAQPAWEQIGIY-RGGIVPVQYRRVSCVKKGGIHFTMNGHTYFNLVLISNVGGAGDVHAVSIK-GSG-TG-WQDMSRNWGQNWQSNG---QFQGQSLSFRVTT-SDGKS 204
SmEXPA2      GWCNTPLQHFDMAQPAFEQIAKY-RGGIVPVLYRRVPCQRKGGIRFTMNGHNYFNLVLVTNVGGAGDVHSVSIK-GSN-TD-WLPMSRNWGQNWQSNA---ILSGQSLSFKVTT-SDGRT 202
SmEXPA3      GWCNPPLEHFDMAQPAWEQIGIY-RGGIVPVQYRRVSCVKKGGIHFTLNGNKYFMLVLVSNVGGAGDVRAVSIK-GPS-GD-WQPMSRNWGQNWQSDS---RLIGQSLSFRVVT-SDNRA 204
SmEXPA4      GWCNSPLQHFDMAQPAFLKIAQY-SAGIVPISYRRVSCSRSGGIRFTMNGHAYFNLVLITNVGGAGDVHAVSIK-GSG-TD-WIPMSRNWGQNWQSNA---LLGGQALSFKVTT-SDGKT 202
SmEXPA5      GWCNPPRVHFDMSKPAFMKIAFW-RAGIIPVSYRRVPCVRSGGMNFKLGGNRWWLMVFITNVGGSGDIKAVSVK-GSR-TG-WIAMTRNWGVGFQVFK---QLQGQSLSFMVTCYSTGKT 202
SmEXPA6      GWCNPPRIHFDMSKPAFSKIAYW-RAGIVPVRYRRVPCRRKGGIKFELKGNRWWLIVFVSNVGGPGDIKRMAVK-GSK-TG-WLPMSRNWGVGFQVFK---SLHGQSLSFMVTSFTTGKT 211
SmEXPA7      GWCDPPKPHFDLSQPAFSRIARI-PNGHAQIQYRRVKCQRQGGIRFTINGHTYFNLVLVTNVGGMGDVVGVSIK-GSS-SG-WRSMSRNWGQNWEEGS---NLNGQALSFRVTT-SDGRT 196
SmEXPA8      GWCDPPKQHFDLSQPAFSQIASI-PYGHVLLQYRRVPCQRQGAIHYTINGHTFFNLVLIENVGGSGDVVGVEIK-GSN-TN-WMPMARNWGQNWMIGG---NLGGQSLSFRVTG-SDGRK 196
SmEXPA9      GWCNPPREHFDLSQPAFELIAKY-RGGIVPVQYRRVPCEREGGIHFSINGHAYFMLVLVWNVGGAGDVHAVAVM-GSRTRR-WQPLVRNWGQNWQSPD---VLLGQSLSFMVTT-SNGDT 206
SmEXPA10     GWCNYPQQHFDLSMPAFVHLANR-TAGIIPVIYTRVECKRQGGIRFTMRGNKWFILVMISNVGGAGDVRSVVVK-GS---RSWTPATRAWGQNWHISNR--SMLEQGLSFVVST-SDGES 203
SmEXPA11     SWCNEPLKHFDMSQPAWEEIGIY-RGGIIPVYFRRVSCVRKGGIHFTVNGHAYFNLILITNVGGAGDVHAVSVK-GSG-TG-WIPMSRNWGQNWQTNA---QLGGQSLSFMVTD-SSGKT 196
```

```
SmEXPA12      GWCNTPREHFDLSQPVWEQMAIY-QGGIVPVQYRRVKCYKQGGIIFTMNGNPNFNLVLIKNVAGWGDLRAVSIK-GSN-TG-WLPMKRNWGSNWEYHG---VLVGQSLSFLLTP-SMGGS 201
SmEXPA13      GWCDSPKQHFDLAQPAFALIAVT-LNGHVPIQYRRVSCKRDGGLRFTINGHVYFNLVLIENVGGTGDVSAVSIK-GSK-TG-WRPMTRNWGQNWQDGG---DLTGQSLSFEVTT-SDGSK 199
SmEXPA14      GWCDPPKPHFDLSQPAFSRIASI-PNGHVQLQYRRVACDRQGGIRFTVNGHTFFNLVLVENVGGSGDVVAVEVK-GSA-TG-WRQMQRNWGQNWQDMG---DLNGQALSFRVTG-SDGKV 198

Majority      VTSYNVAPAN-----------------WQFG----------------QTFEG-------
              ____________________________________________________________
                       |         |         |         |         |
                      250       260       270       280       290
              ____________________________________________________________
AtEXPA12.seq  QTFLNVVPSS-----------------WWFG----------------QTFSSRV     222
AtEXPA17.seq  KAALNVVPSN-----------------WRFG----------------QSFKSNV     226
AtEXPA11.seq  RVFLNVVPSS-----------------WSFG----------------QIYSSNV     225
AtEXPA22.seq  KDFWNVMPKN-----------------WGFG----------------QTFDGRM     218
AtEXPA14.seq  VISNNATPRN-----------------WSFG----------------QTYTG---K  221
AtEXPA15.seq  VVSNNIAPAS-----------------WSFG----------------QTFTG---R  221
AtEXPA8.seq   LVSNDVAPSN-----------------WQFG----------------QTYQG---G  222
AtEXPA22.seq  KDFWNVMPKN-----------------WGFG----------------QTFDGRM     218
AtEXPA4.seq   STSWNMVPSN-----------------WQFG----------------QTFVG---K  223
AtEXPA13.seq  VTSYNVSPKN-----------------WNYG----------------QTFEGK      222
AtEXPA20.seq  IASYDVAPPY-----------------WRFG----------------MTYQGK      225
AtEXPA7.seq   IYAWNVAPAN-----------------WSGG----------------KTYKSTV     223
OsEXPA12.seq  LVFEDVAPSN-----------------WQFG----------------QTFTSGA     223
OsEXPA33.seq  VTVWNIAPSN-----------------WNFG----------------TTYTSNM     224
OsEXPA4.seq   VTSNNVAHPG-----------------WQFG----------------QTFEG---G  221
OsEXPA5.seq   VVSNNAVPRG-----------------WSFG----------------QTFSG---A  225
OsEXPA11.seq  VTSDNVAPSG-----------------WSFG----------------QTFSG---G  220
OsEXPA7.seq   STSWNAAPAG-----------------WHFG----------------QTFEG---K  225
OsEXPA32.seq  ATSWHVLPPD-----------------WQFG----------------VTYQATM     225
OsEXPA16.seq  VVAYSVAPPD-----------------WMFA----------------QTFEGK      225
OsEXPA10.seq  LTNYNVVPKE-----------------WDFG----------------KTYTGK      221
OsEXPA30.seq  ILAAGVTPAS-----------------WCFG----------------LTYQARV     227
PpEXPA9.seq   VASFDAVPAN-----------------WAFS----------------QTFEGS      223
PpEXPA8.seq   VVSNNVAPPD-----------------WAFG----------------QTYEGS      226
PpEXPA7.seq   AVSMDAAPGN-----------------WQFG----------------QTFEGV      222
PpEXPA6.seq   SVTKLAK-SD-----------------WCFG----------------KTFIG---K  220
PpEXPA5.seq   AISPNVAPAN-----------------WGFG----------------QTFEGS      220
PpEXPA4.seq   LVSNRA--VG---------------SWWKFG----------------QTWEGS      222
PpEXPA3.seq   AESLNAAPSN-----------------WGWG----------------QTFEGV      221
PpEXPA27.seq  VVSIDAAPAN-----------------WNFG----------------QTFSSG      215
PpEXPA26.seq  LILYNAVRKG-----------------WRFG----------------QTWEGS      219
PpEXPA25.seq  ITINSAVGNA-----------------WKFG----------------QTWEGH      223
PpEXPA24.seq  VTTHNAVGHG-----------------WQFG----------------QTWEGA      226
PpEXPA23.seq  VTTYNAVGNG-----------------WKFG----------------QTWEGA      226
PpEXPA22.seq  LTAYNAVGNG-----------------WTFG----------------QTWEGA      223
PpEXPA21.seq  LITRNAVGSG-----------------WRFG----------------QTWEG       222
PpEXPA20.seq  IVSNRAANVGGLPVQIVPLGPIAILSLSRKGPFRSKLNDAPRTLMCLQEWPVRP     254
```

```
PpEXPA2.seq  VTVNRAVGDY-----------------WKFG----------------QTWEGS          222
PpEXPA19.seq LYSMNAVRNG-----------------WKFG----------------QTWEGS          219
PpEXPA18.seq LTTYKAVGGY-----------------WRFG----------------QTWEGS          222
PpEXPA17.seq AISNNVASSN-----------------WGFG----------------QTFEGS          220
PpEXPA16.seq VVSYNAAPAN-----------------WRFG----------------QTFSSG          221
PpEXPA15.seq AISYDAAPNN-----------------WQFG----------------QTFEGV          222
PpEXPA14.seq VISYNAAPAN-----------------WWFG----------------XTFSSG          221
PpEXPA13.seq VVSRNVAPSD-----------------WAYG----------------QTYEGS          223
PpEXPA12.seq AVSYDAASEN-----------------WSFS----------------QTFEGI          217
PpEXPA11.seq AVSNNVAPPN-----------------WGFG----------------QTFEGX          220
PpEXPA10.seq VESLDAAPAN-----------------WHFG----------------QMFEGS          221
PpEXPA1.seq  IVSMNATPSH-----------------WSFG----------------QTFEG---G       221
PtEXPA26.pro STSWHVTPKN-----------------WQFG----------------QTYEG           282
SmEXPA1      VVSMDVAPAD-----------------WQYG----------------QTFEG---SQFV    227
SmEXPA2      VVSYDAAPPN-----------------WQYG----------------QTYSG---DQF     224
SmEXPA3      VTSLNVAPAG-----------------WSFG----------------QTFSG---EQF     226
SmEXPA4      TIAYNVAGAN-----------------WAYG----------------QTFEG---EQF     224
SmEXPA5      TVHNNVAPAN-----------------WQLG----------------STYSAKQL       224
SmEXPA6      VTAYDVVPAN-----------------WRIG----------------QAYSGGQMV      234
SmEXPA7      VTAYNVAPGD-----------------WQFG----------------RTYTGNTASQYL   222
SmEXPA8      VTSLNVAPAN-----------------WQFG----------------RAYSG---GQFL   219
SmEXPA9      VTDYDVAPQD-----------------WKFG----------------QTFVG---NKNL   229
SmEXPA10     RIALDVVPRN-----------------WKFG----------------QTFTTGAQFL     227
SmEXPA11     VISNNAAPSN-----------------WQYG----------------QTFEG---EQFL   219
SmEXPA12     LISYDVFPRN-----------------WQFG----------------QSYSG---RQFM   224
SmEXPA13     ITAYDVAPDY-----------------WQFG----------------QTFSG---GQFL   222
SmEXPA14     VTSMNVAPAD-----------------WQFG----------------RTYSG---GQFL   221
```
