# Supplementary figures and images for: Selaginella moellendorffii has a reduced and highly conserved expansin superfamily with genes more closely related to angiosperms than to bryophytes
Source: BMC Plant Biol. 2013 Jan 3;13:4. doi: 10.1186/1471-2229-13-4 (PMC3680112; doi:10.1186/1471-2229-13-4)

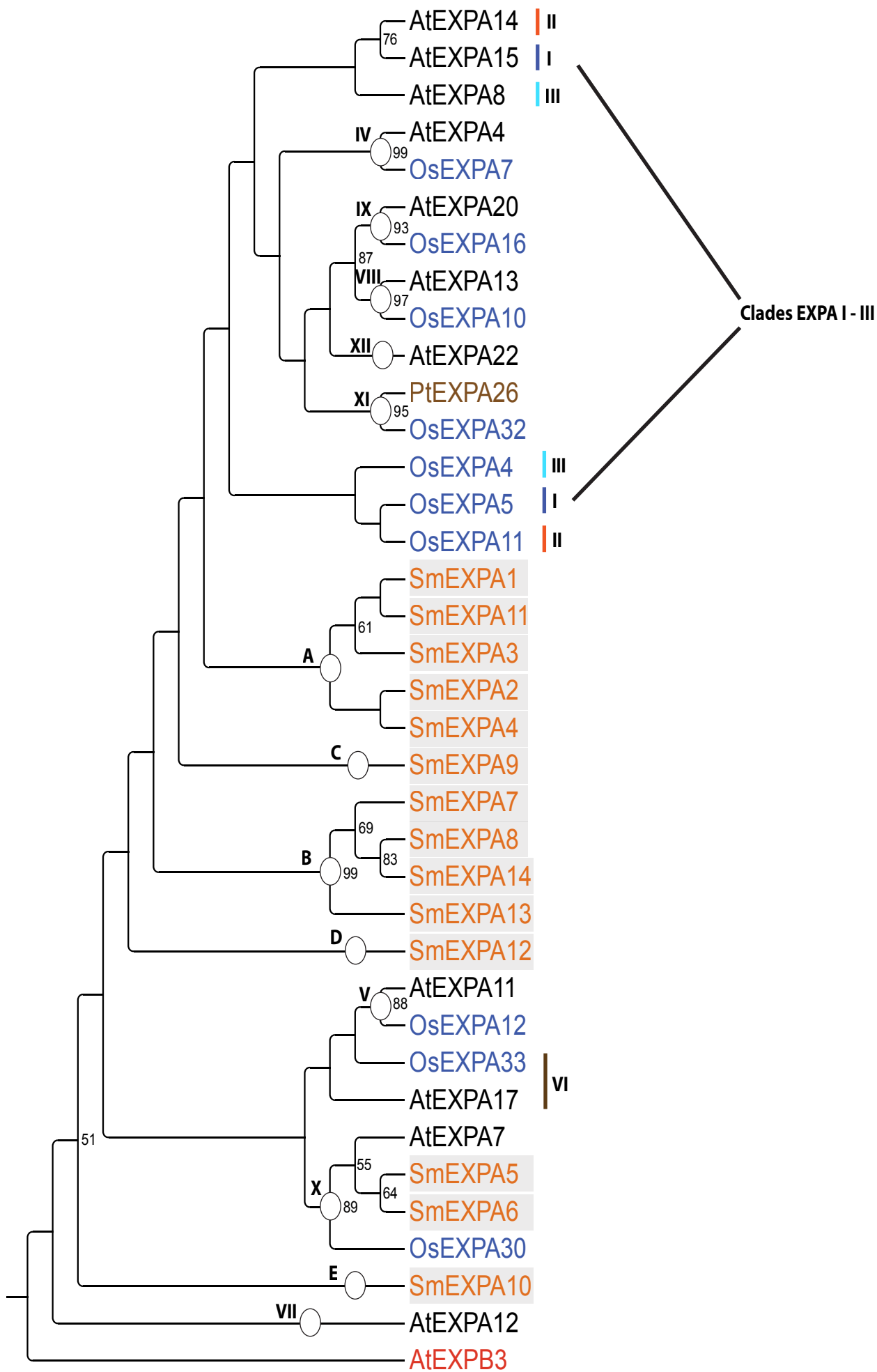

Supplement: Additional file 4 — One of four maximum parsimony Selaginella EXPA trees of length 1901. Significant bootstrap values from bootstrap consensus tree are indicated. Tree was rooted manually at AtEXPB3. Clade and groupings are marked with circles (or bars when they are poorly resolved as in the case of clades EXPA – I, EXPA – II, EXPA – III, and EXPA – VI). Selaginella sequences are labeled in orange and boxed, rice sequences in blue, Arabidopsis in black, and a Populus sequence in brown. [file 1471-2229-13-4-S4.pdf]

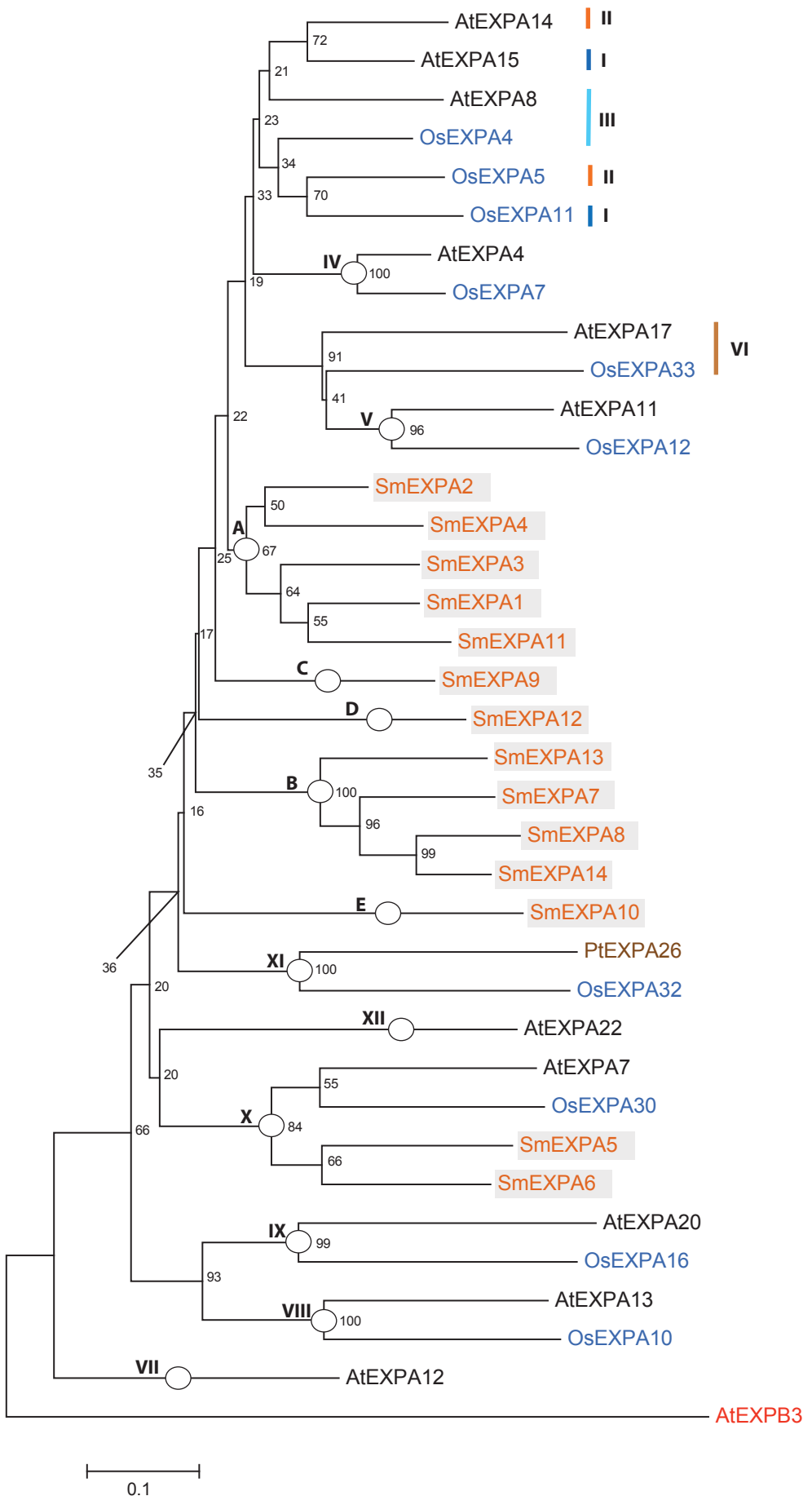

Supplement: Additional file 5 — Neighbor joining tree for the Selaginella EXPA family. Tree was rooted manually at AtEXPB3. Clade and groupings are marked with circles (or bars when they are poorly resolved as in the case of clades EXPA – I, EXPA – II, EXPA – III, and EXPA – VI). Selaginella sequences are labeled in orange and boxed, rice sequences in blue, Arabidopsis in black, and a Populus sequence in brown. [file 1471-2229-13-4-S5.pdf]

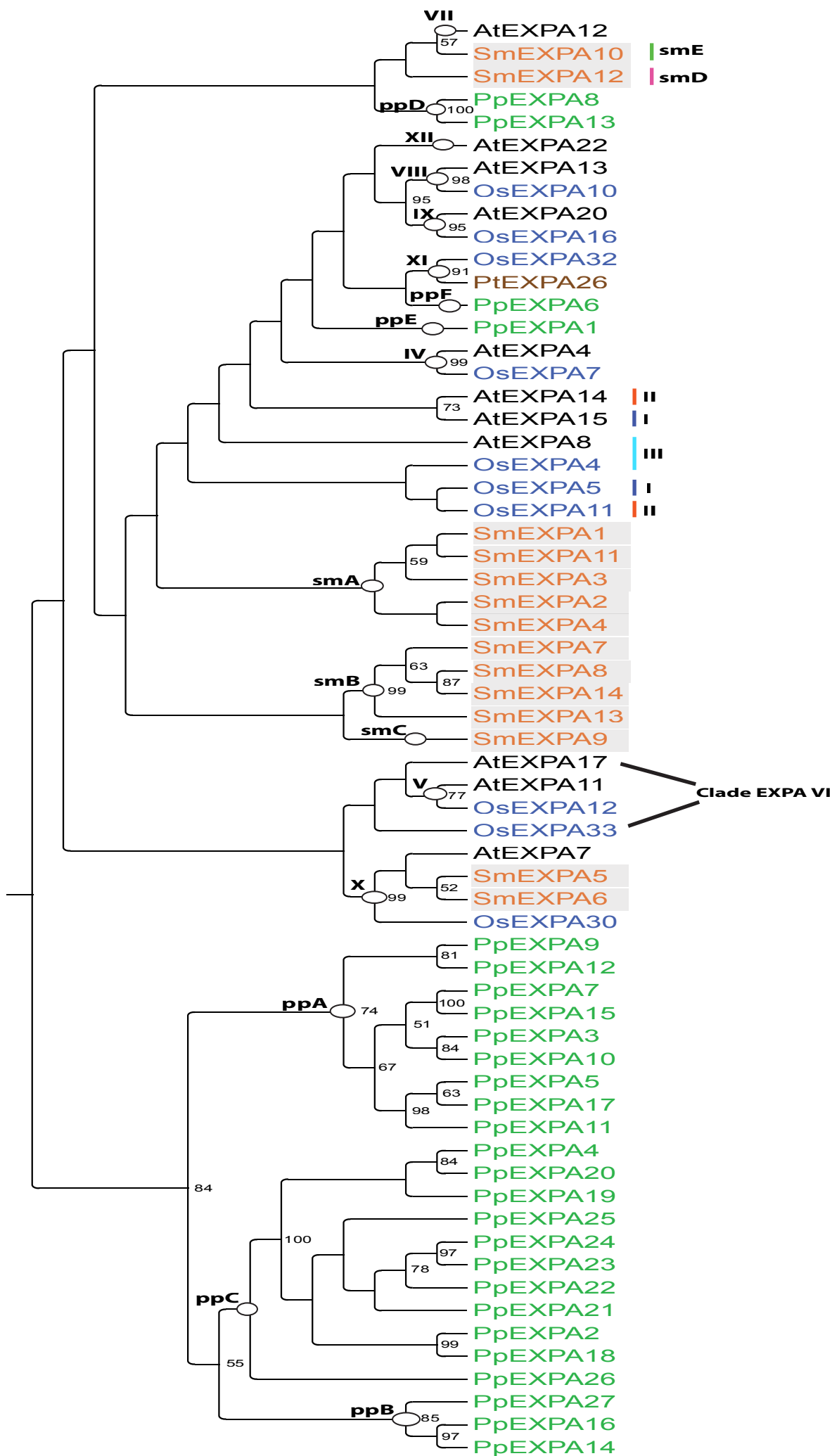

Supplement: Additional file 7 — One of thirty-four maximum parsimony Selaginella and Physcomitrella EXPA trees of length 2871. Significant bootstrap values from bootstrap consensus tree are indicated. Tree was rooted manually at Physcomitrella patens groups A-C. Clades and groupings are marked with circles (or bars when they are poorly resolved as in the case of clades EXPA – I, EXPA – II, EXPA – III and EXPA – VI or the tree has become cluttered as in the case of clades smD and smE). Selaginella sequences are labeled in orange and boxed, Physcomitrella patens in green, rice sequences in blue, Arabidopsis in black, and a Populus sequence in brown. [file 1471-2229-13-4-S7.pdf]

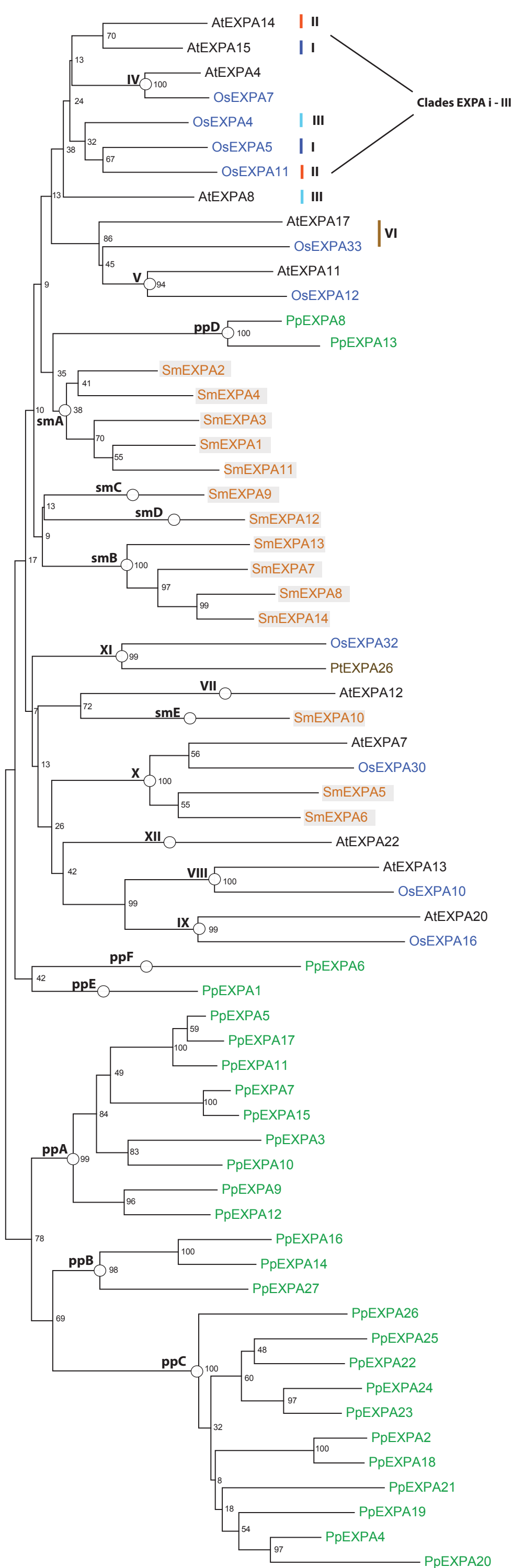

0.05

Supplement: Additional file 8 — Neighbor joining tree for Selaginella and Physcomitrella EXPA family. Tree was rooted manually at Physcomitrella patens groups A-C. Clade and groupings are marked with circles (or bars when they are poorly resolved as in the case of clades EXPA – I, EXPA – II, EXPA – III, and EXPA – VI). Selaginella sequences are labeled in orange and boxed, Physcomitrella patens in green, rice sequences in blue, Arabidopsis in black, and a Populus sequence in brown. [file 1471-2229-13-4-S8.pdf]

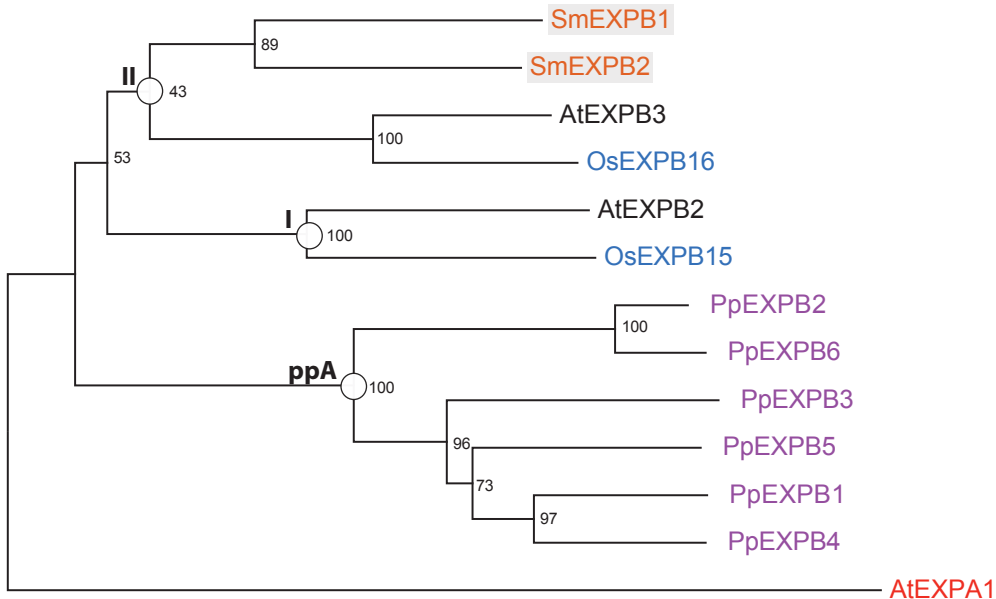

0.1

Supplement: Additional file 10 — Neighbor joining tree for EXPB family. Tree was rooted manually at AtEXPA1. Clade and groupings are marked with circles. Selaginella sequences are labeled in orange and boxed, Physcomitrella patens in purple, rice in blue, and Arabidopsis in black. [file 1471-2229-13-4-S10.pdf]

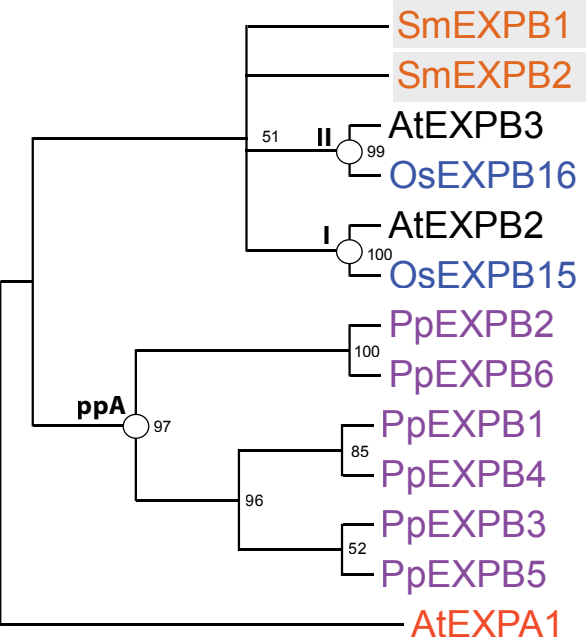

Supplement: Additional file 11 — Bootstrap consensus parsimony tree obtained for the EXPB family. Tree was rooted manually at AtEXPA1. The original analysis recovered one most parsimonious tree of length 796. Clade and groupings are marked with circles. Selaginella sequences are labeled in orange and boxed, Physcomitrella patens in purple, rice in blue, and Arabidopsis in black. [file 1471-2229-13-4-S11.pdf]

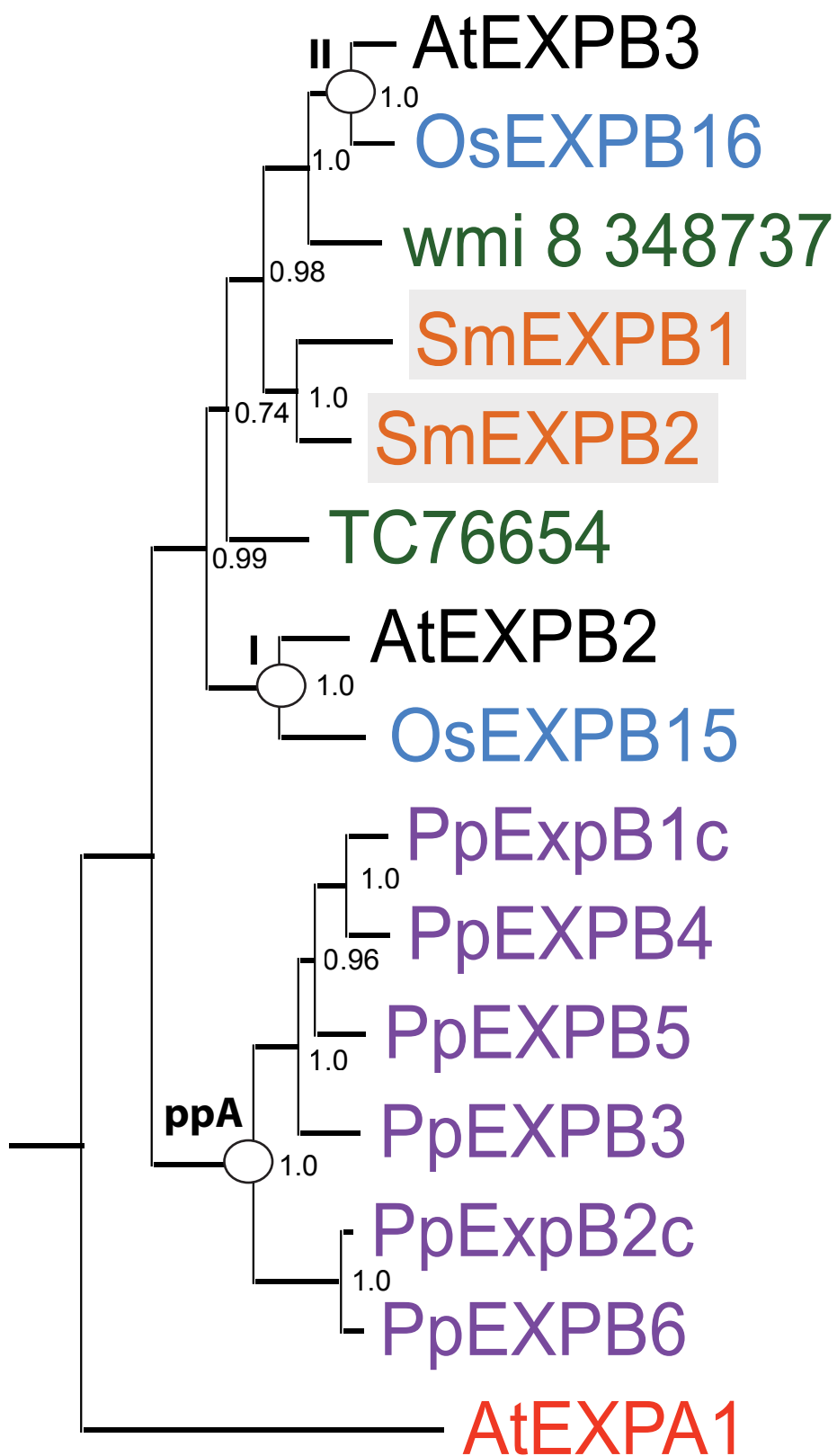

Supplement: Additional file 12 — Bayesian likelihood tree for Selaginella and Physcomitrella EXPB genes with selected rice, Arabidopsis, Populus, and gymnosperm sequences. 40,000 trees were collected. Burnin was set to 10,000. Clade and groupings are marked with circles. Selaginella sequences are orange and boxed, Physcomitrella in purple, rice in blue, and Arabidopsis in black. Gymnosperm sequences are green. ‘TC’ numbers are TIGR numbers for Pinus ESTs. Wmi is Welwitschia mirabilis from the Floral Genome Project (http://www.floralgenome.org/). [file 1471-2229-13-4-S12.pdf]

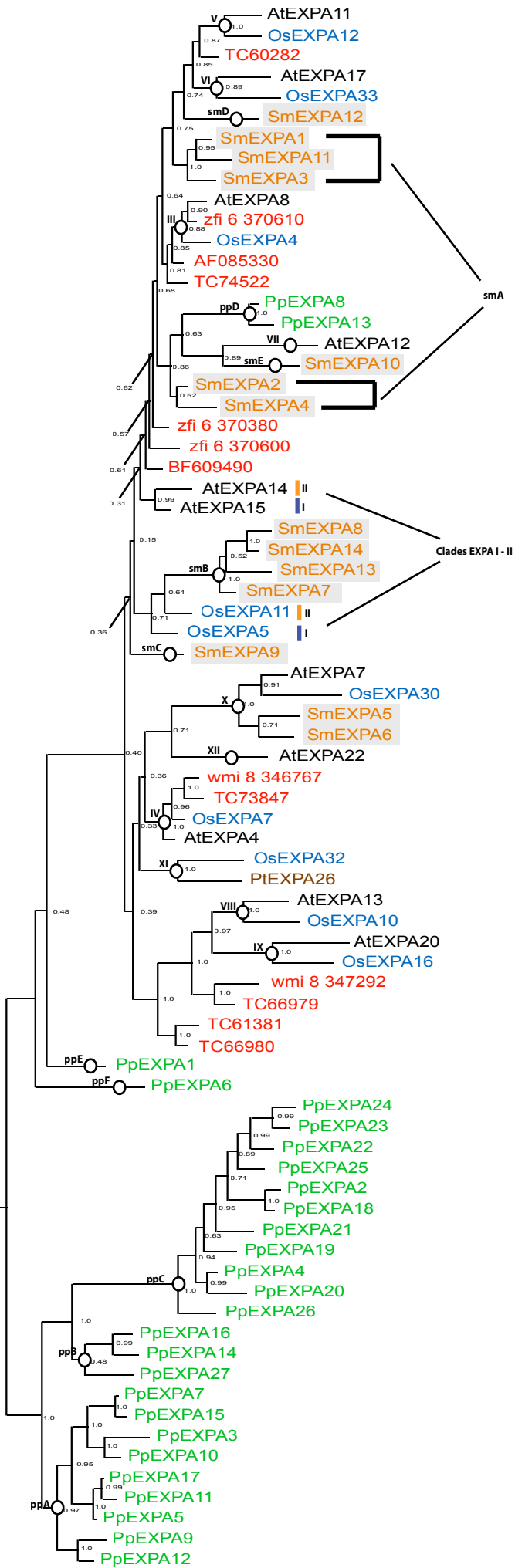

Supplement: Additional file 13 — Bayesian likelihood tree for Selaginella and PhyscomitrellaEXPA genes with selected rice, Arabidopsis, Populus, and gymnosperm sequences. Clade and groupings are marked with circles. Selaginella sequences are orange and boxed, Physcomitrella in green, rice in blue, and Arabidopsis in black and Populus in brown. Gymnosperm sequences are in red. ‘TC’ numbers are TIGR numbers for Pinus ESTs. Wmi and zfi are Welwitschia mirabilis and Zamia fisheri cDNA from the Floral Genome Project (http://www.floralgenome.org/). [file 1471-2229-13-4-S13.pdf]

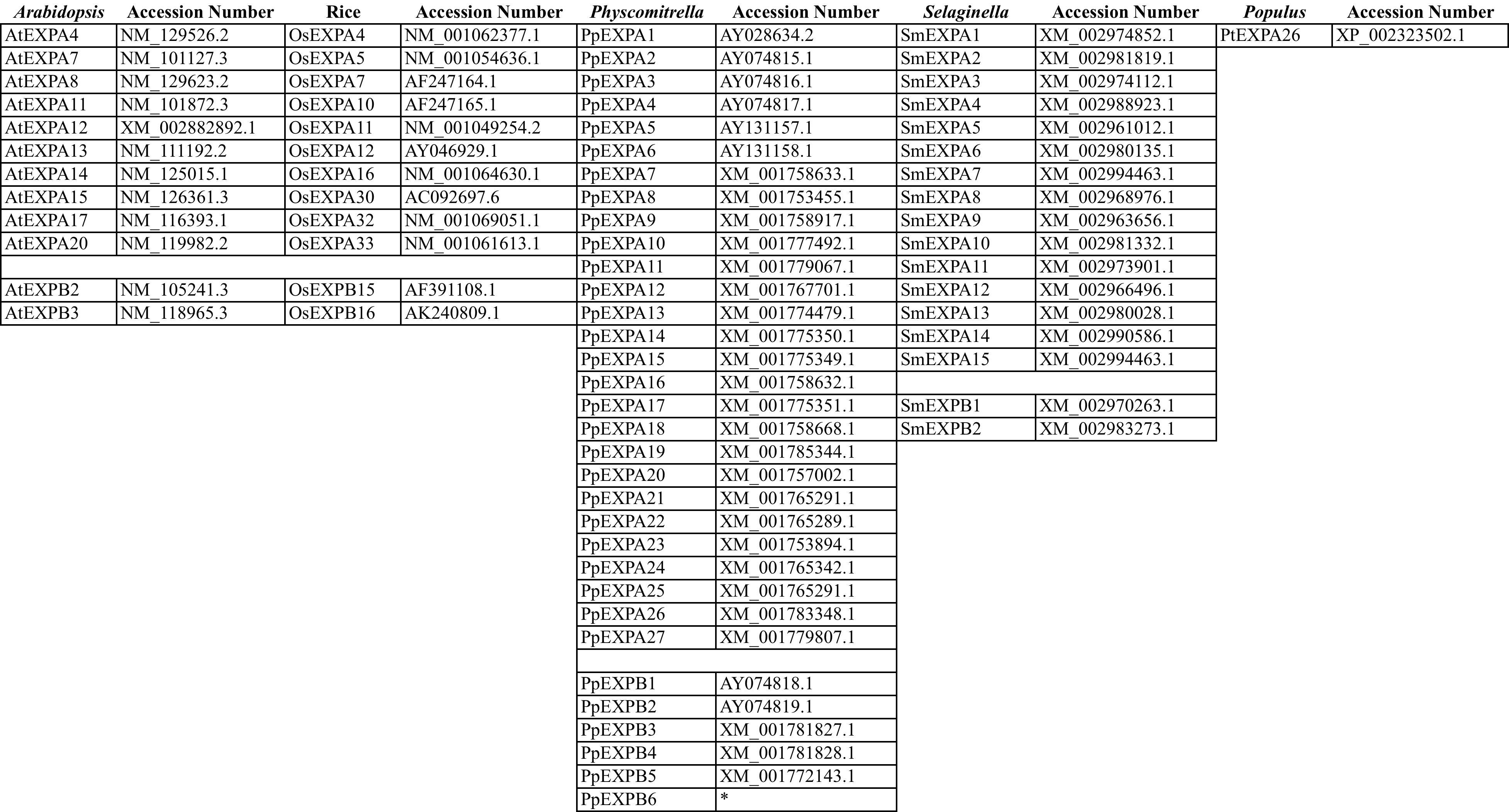

Supplement: Additional file 16 — GenBank Accession Numbers. Accession numbers for Arabidopsis, rice, Selaginella, Physcomitrella, and Populus sequences used for alignments and phylogeny building. Note that not all Physcomitrella sequences have GenBank entries. Please consult the Physcomitrella genome v1.1 (http://genome.jgi-psf.org/physcomitrella/physcomitrella.info.html) for missing sequences. [file 1471-2229-13-4-S16.docx]
