# Supplementary material for: Selaginella moellendorffii has a reduced and highly conserved expansin superfamily with genes more closely related to angiosperms than to bryophytes
Source: BMC Plant Biol. 2013 Jan 3;13:4. doi: 10.1186/1471-2229-13-4 (PMC3680112; doi:10.1186/1471-2229-13-4)
Supplement: Additional file 6 — The lowest Poisson-corrected amino acid distance of each Selaginella EXPA gene to an Arabidopsis and rice expansin. The clades from which each of these Arabidopsis and rice genes come from is also given. Clades highlighted in yellow are those other than EXPA I-IV. Note that SmEXPA5 and SmEXPA6 have their lowest distance to members of clade EXPA – X. [file 1471-2229-13-4-S6.docx]

| **SmA** | **AtEXPA** | **Distance** | **Clade** | **OsEXPA** | **Distance** | **Clade** |
| --- | --- | --- | --- | --- | --- | --- |
| SmEXPA1 | AtEXPA10 | 0.285 | I | OsEXPA2 | 0.309 | III |
| SmEXPA2 | AtEXPA10 | 0.249 | I | OsEXPA2/OsEXPA4 | 0.303 | III |
| SmEXPA3 | AtEXPA3 | 0.313 | IV | OsEXPA2 | 0.285 | III |
| SmEXPA4 | AtEXPA1 | 0.311 | I | OsEXPA4 | 0.342 | III |
| SmEXPA11 | AtEXPA1 | 0.342 | I | OsEXPA4 | 0.342 | III |
|  |  |  |  |  |  |  |
| **SmB** |  |  |  |  |  |  |
| SmEXPA7 | AtEXPA10 | 0.425 | I | OsEXPA11 | 0.377 | II |
| SmEXPA8 | AtEXPA1/AtEXPA10 | 0.465 | I | OsEXPA11 | 0.377 | II |
| SmEXPA13 | AtEXPA1 | 0.453 | I | OsEXPA11 | 0.441 | II |
| SmEXPA14 | AtEXPA1 | 0.419 | I | OsEXPA11 | 0.394 | II |
| SmEXPA15 | AtEXPA10 | 0.42 | I | OsEXPA11 | 0.372 | II |
|  |  |  |  |  |  |  |
| **SmC** |  |  |  |  |  |  |
| SmEXPA9 | AtEXPA10/AtEXPA15 | 0.328 | I | OsEXPA4 | 0.359 | III |
|  |  |  |  |  |  |  |
| **SmD** |  |  |  |  |  |  |
| SmEXPA12 | AtEXPA3 | 0.419 | IV | OsEXPA2 | 0.449 | III |
|  |  |  |  |  |  |  |
| **SmE** |  |  |  |  |  |  |
| SmEXPA10 | AtEXPA1 | 0.482 | I | OsEXPA2 | 0.544 | III |
|  |  |  |  |  |  |  |
| **X** |  |  |  |  |  |  |
| SmEXPA5 | AtEXPA7 | 0.353 | X | OsEXPA30 | 0.497 | X |
| SmEXPA6 | AtEXPA7 | 0.397 | X | OsEXPA2 | 0.487 | III |

**Supplemental Tables**

| **Supplemental Table 1 – The lowest Poisson – corrected amino acid distance of each *Selaginella* EXPA gene to an *Arabidopsis* and rice expansin** |
| --- |
