# Supplementary material for: Selaginella moellendorffii has a reduced and highly conserved expansin superfamily with genes more closely related to angiosperms than to bryophytes
Source: BMC Plant Biol. 2013 Jan 3;13:4. doi: 10.1186/1471-2229-13-4 (PMC3680112; doi:10.1186/1471-2229-13-4)
Supplement: Additional file 9 — Alignment for Figure 3. Alignment of Selaginella and Physcomitrella EXPB sequences with selected Arabidopsis and rice EXPB genes. [file 1471-2229-13-4-S9.pdf]

```
Majority      WVPAHATWYGDPYGEGSXGGACGYGXLVG-TPYGXKXXAGSPSIFKNGXGCGACYEVKCXDPS----XCSPQPVTVVITDECP--------GG-YCSTGXXHFDLSGAAF
                        10        20        30        40        50        60        70        80        90       100       110

AtEXPA1.seq   WVNAHATFYGGGDASGTMGGACGYGNLYS-QGYGTNTAALSTALFNNGLSCGACFEIRCQNDG---KWCLPGSIVVTATNFCPPNNALPNNAGGWCNPPQQHFDLSQPVF 106
AtEXPB2.seq   WSIAGSTWYGNPTGYGSDGGACGYGNAVAQPPFSKMVSAGGPSLFKSGKGCGACYQVKCTSKS----ACSKNPVTVVITDECP--------G---CVKESVHFDLSGTAF 95
AtEXPB3.seq   WLPAVATWYGSPNGDGSDGGACGYGTLVDVKPLHARVGAVNPILFKNGEGCGACYKVRCLDKS----ICSRRAVTVIITDECP--------G---CSKTSTHFDLSGAVF 95
OsEXPB15.seq  WSNGGATWYGAANGAGSDGGACGYQGAVFQAPFSSMIAAGSPSIYKSGLGCGSCYQVKCTGNS----ACSGNPVTVVLTDECP--------GG-PCLSEPVHFDLSGTAF 97
OsEXPB16.seq  WHPATATWYGSADGDGSDGGACGYGTLVDVVPMKTRVGAVSPVLFKGGEGCGACYKVRCLDAS----ICSRRAVTVIVTDECP--------GG-VCAFGRTHFDLSGAAF 97
PpExpB1c.seq  WLGGHATWYGDPYGEGSSGGACGYTQLTG-TPIGSKIAAGNAPIFQEGKGCGQCYEVKCNYPS-----CSPQGTRIVITDLCP--------GGQYCSTDQPAFDFSGAAI 96
PpExpB2c.seq  WVRARGTWYGDPYGEGSSGGNCGYTKLWG-TPIGPKIVAGSRSIYANGQGCGQCYQIRCVDPNGGPRLCNPQGTNVVVTDFCP--------GGTYCSTGENAFDMSGAAI 101
PpEXPB3.seq   WLPGHATWYGDPYGEGSSGGACGYTELAG-TPYGLSVGAGSAVIYQNGQGCGECYEVKCTYPS-----CKPTPSRIVITDFCP--------GGTFCSTGEPAFDLSGMAM 96
PpEXPB4.seq   WVPGHATWYGDPYGEGSSGGXCGYLQLTG-TPIGNKIAAGNEAIFQKGKGXXQCYEVKCNYPV-----CRPEGTRIVITDLCP--------GXQFCSGGNPAFDLSGAAI 96
PpEXPB5.seq   WMAGHATWYGEPLGEGSSGGACGYTKLAD-TPYGPKIAAGNDPIFQGGSGCGACFEVKCNYPS-----CKSEPTRIIITDQCP--------GGTYCSTSQPAFDLSGAAI 96
PpEXPB6.seq   WVRARGTWYGDPYGEGSSGGNCGYTRL.G-TPIGPKIVAGSRSIYANGLGCGQCYQNQCVDPXXGXKLXNSFXTQXXXTDFCP--------GGTYCSTGENAFDMSGAAI 101
SmEXPB1       WEPATATWYGSPNGAGTDGGACGYGSLPN-TPYGSDVGAGSPILFMNGIGCGTCFEVKCVDGQ----LCSPQPVNVVITDECP--------GG-YCSGGRTHFDLSGTAF 96
SmEXPB2       WQLATATWYGSPSGAGTDGGACGYGELPN-TPYGLDVGAGSPVLFKNGRGCGACYKVRCLQQQ----LCSGKAVTVVITDECP--------GG-YCAFGRTHFDLSGTAF 96

Majority      GAMALPGR-DGQLRNRGLYXILYKRVPCXYXGQNIAFQVDXGSXPFWLSXLVKYVGGPGDIGSVEIKQAG-SSAWQPMXHSWGANWMLINY-G-PXXGPFSIXITXLLNG
                       120       130       140       150       160       170       180       190       200       210       220

AtEXPA1.seq   QRIAQYRA--------GIVPVAYRRVPCVRRG-GIRFTIN--GHSYFNLVLITNVGGAGDVHSAMVKGSR--TGWQAMSRNWGQNWQSNSYLN---GQSLSFKVTTS-DG 199
AtEXPB2.seq   GAMAISGQ-DSQLRNVGELQILYKKVECNYIGKTVTFQVDKGSNANSFAVLVAYVNGDGEIGRIELKQALDSDKWLSMSQSWGAVWKLDVS--SPLRAPLSLRVTSLESG 202
AtEXPB3.seq   GRLAIAGE-SGPLRNRGLIPVIYRRTACKYRGKNIAFHVNEGSTDFWLSLLVEFEDGEGDIGSMHIRQAG-AREWLEMKHVWGANWCIIGG---PLKGPFSIKLTTLSAG 200
OsEXPB15.seq  GAMANPGQ-ADQLRAAGVLQIQYNRVPCNWGGVKLTFVVDVGSNPNYFAVLVKYENGDGDLSGVELMQTGAGAAWTQMQQSWGAVWKLNAG--SALQAPFSIRLTSS-SG 203
OsEXPB16.seq  ARLAVAGH-GGQLQNRGEISVVYRRTACKYGGKNIAFHVNEGSTTFWLSLLVEFEDGDGDIGSMQLKQAN-SAQWQDMKHIWGATWSLTPG---PLVGPFSVRLTTLTTR 202
PpExpB1c.seq  TAMALPGR-DGELRNIGLYDIQYKRVPCEYPNQNIAFKVDAGSSKYWFSFTVKYLGGPGDINTVEVKCGK-NGYFQYAQHSWGANWMLINYSGVPFQFPLTIKITTKLND 204
PpExpB2c.seq  NAMALRGR-EGQLRNRGLYNLLYKRVPCRYRGTNIEFRVDNGSSPFWLSILIKYVGGPGDIGQVYIRMAN-WYKFQPMRHAWGANWMIPNYDGKPFRGPMDIRIVSRLNR 209
PpEXPB3.seq   TNMALPGR-DQELRNLGLYEIQYRRVPCYYPNQNVAFKVDPGSTPFWLSFTIEYQGGPGDIESVAIRQAG-SSEFQQMQHNWGANYMLICYSGKPFKGPYDVMITAKLNG 204
PpEXPB4.seq   SAMAKDGQ-DGALRNIGLYDIQYKRVPCEYPGQNIVFKVDAGSSPFWLSFTVKYMGGPGDIESVSISQ-R-DGSFIPAQHSWGANWMLINYSGAPFQGPYSVKINCMLNG 203
PpEXPB5.seq   SDMAVSGK-DGELRNIGLYDILYKRVPCEYPNQNIAFQVDAGSSAFWLSLLVKYMGGPGDIESVEIRTTG-SSSFQPAKHNWGASWMLINTSGQPFKGPYDVKIVSKLNG 204
PpEXPB6.seq   NAMALPSR-EGQLRNRGLYNLLYKRVPCRYRGTXIXXRVDXGSSAYWLSILIKYVGGPGDIGQVYIKMAN-WFAFQPMKHAWGANWMMPSYDGKPFKGPMDIKIVSKLNR 209
SmEXPB1       GKMASGSANIQHLLAAGVLNVLYRRAPCIYKSQGVVFQVADGSTPFWFETVIRYLDGPGDLATVELQQFG-SSAWQPMSQVWGANWCLNAGGGTPLRAPFSIRLTALQTG 205
SmEXPB2       GRMAMAGR-TNQLLGSGVTQVLYKRVDCNYGSRPMEFQVNEGSTPFWLSILVRYAAGPGDLGHVELMQAG-SRVWQPMTQVWGATWCFNGG---PLRGPFSFRVTTLSTS 201
```

```
Majority      HTVVARXVIPANWAPGXTYESXVXF-

                      230       240

AtEXPA1.seq   QTIVSNNVANAGWSFGQTFTGAQLR     224
AtEXPB2.seq   KTVVASNVIPANWQPGAIYKSNVNF     227
AtEXPB3.seq   KTLSATDVVPRNWAPKATYSSRLNF     225
OsEXPB15.seq  KTLVASNVIPSGWKPGMSYISTVNF     228
OsEXPB16.seq  QTLSAQDVIPKNWTPKATYTSRLNF     227
PpExpB1c.seq  HTVVAEDVIPDWFGPGVQYESNVQI     229
PpExpB2c.seq  HTVLARGVIPAYFRPGTSYRSRVQM     234
PpEXPB3.seq   HVLIAQDAIPEYFQPGGIYESNVQL     229
PpEXPB4.seq   HTVVAKDVIPAGFAPGQEYESNVQI     228
PpEXPB5.seq   HTVIAEKAIPEFFEPGKLYESNVQM     229
PpEXPB6.seq   HSVVARGVIPGYFRPGTTYNSRVQM     234
SmEXPB1       EKIIAHNVIPANWAPQHSYSTGVNFV    231
SmEXPB2       ETVVARNVIPRNWASNTCYRSRVNFM    227
```
