# Supplementary material for: Selaginella moellendorffii has a reduced and highly conserved expansin superfamily with genes more closely related to angiosperms than to bryophytes
Source: BMC Plant Biol. 2013 Jan 3;13:4. doi: 10.1186/1471-2229-13-4 (PMC3680112; doi:10.1186/1471-2229-13-4)
Supplement: Additional file 14 — Average nucleotide composition of Arabidopsis, rice, Selaginella, and Physcomitrella EXPA genes. Values given are a percentage of all nucleotides in a dataset trimmed as was done for the phylogenies presented here. [file 1471-2229-13-4-S14.docx]

| **Species** | **Ave % T** | **Ave % C** | **Ave % A** | **Ave % G** |
| --- | --- | --- | --- | --- |
| *Selaginella* | 19.8 | 28.7 | 21.9 | 29.7 |
| *Arabidopsis* | 25.3 | 22.8 | 26.1 | 25.9 |
| Rice | 15.4 | 32.9 | 17.3 | 34.4 |
| *Populus* | 21.9 | 16.9 | 27.5 | 33.8 |
| *Physcomitrella* | 22.2 | 26.4 | 22.1 | 29.4 |

| **Supplemental Table 2 – Average nucleotide composition of *Selaginella*, *Arabidopsis*, rice, *Populus*, and *Physcomitrella* EXPA genes.** |
| --- |
