# Supplementary material for: Selaginella moellendorffii has a reduced and highly conserved expansin superfamily with genes more closely related to angiosperms than to bryophytes
Source: BMC Plant Biol. 2013 Jan 3;13:4. doi: 10.1186/1471-2229-13-4 (PMC3680112; doi:10.1186/1471-2229-13-4)
Supplement: Supplementary file 8 [file 1471-2229-13-4-S15.docx]

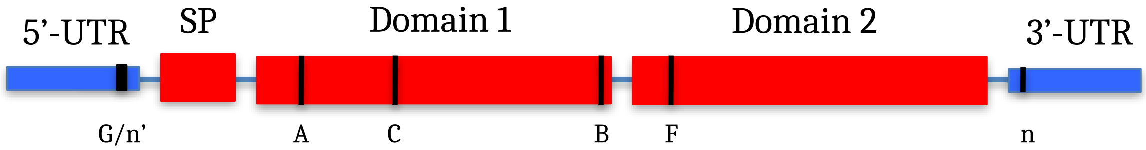
5'-UTR
SP
Domain 1
Domain 2
3'-UTR
G/n'
A
C
B
F
n
